# bayesReact: expression-coupled regulatory motif analysis detects microRNA activity across cancers, tissues, and at the single-cell level

**DOI:** 10.1093/nar/gkag072

**Published:** 2026-02-09

**Authors:** Asta Mannstaedt Rasmussen, Alexandre Bouchard-Côté, Jakob Skou Pedersen

**Affiliations:** Department of Clinical Medicine, Aarhus University, Palle Juul-Jensens Boulevard 11, 8200 Aarhus N, Denmark; Department of Molecular Medicine, Aarhus University Hospital, Palle Juul-Jensens Boulevard 11, 8200 Aarhus N, Denmark; Department of Statistics, University of British Columbia, 2207 Main Mall, V6T 1Z4 British Columbia, Canada; Department of Clinical Medicine, Aarhus University, Palle Juul-Jensens Boulevard 11, 8200 Aarhus N, Denmark; Department of Molecular Medicine, Aarhus University Hospital, Palle Juul-Jensens Boulevard 11, 8200 Aarhus N, Denmark; Section for Bioinformatics and Computational Biology, Aarhus University, Universitetsbyen 81, 8000 Aarhus C, Denmark

## Abstract

Gene regulatory mechanisms control cell differentiation and homeostasis but are often undetectable, particularly at the single-cell level. We introduce bayesReact, which quantifies regulatory activities from bulk or single-cell omics data. It is based on an unsupervised generative model, exploiting the fact that each regulator typically targets many genes sharing a sequence motif. Using mRNA expression data, we illustrate and evaluate bayesReact on microRNAs (miRNAs). It outperforms existing methods on sparse bulk data and improves activity inference on single-cell data. Inferred miRNA activities correlate with miRNA expression across pan-cancer TCGA and healthy GTEx tissue samples. The activities capture cancer-type-specific miRNA patterns, e.g., for miR-122-5p and miR-124-3p, which also correlate more strongly with their target genes than their measured expression. This includes a strong negative correlation between miR-124-3p and the anti-neuronal REST transcription factor in nervous system cancers. Analyzing single-cell data, bayesReact detects prominent miRNAs during murine stem cell differentiation, including miR-298-5p, miR-92-2-5p, and the Sfmbt2 cluster (miR-297-669). Furthermore, spatio-temporal inference shows increasing miR-124-3p activity in differentiating neurons during embryonic spinal cord development in mice. bayesReact enables large-scale hypothesis-generating screens for novel regulatory factors and the discovery of condition-specific activities. It is implemented as a user-friendly R package (https://github.com/JakobSkouPedersenLab/bayesReact).

## Graphical Abstract

**Graphical Abstract:**
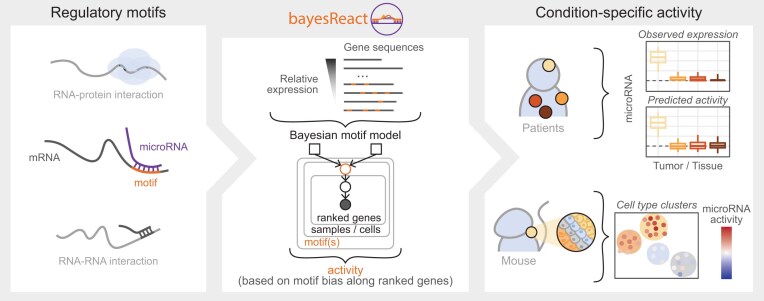

## Introduction

Regulatory mechanisms are vital for maintaining cellular homeostasis, facilitating proper cell proliferation and differentiation, while preserving tissue integrity and preventing carcinogenesis [1, 2]. However, much remains unknown regarding the spatio-temporal complexity of regulatory cell constraints, with new regulators still being discovered and characterized [3–5]. Regulatory mechanisms are frequently facilitated through motif recognition [6, 7], where a motif is a distinct biological pattern, e.g., a nucleotide (nt) or peptide sequence. Regulatory motif representations range from short strings to complex regular expressions (REs) and position weight matrices (PWMs), with examples including binding sites for transcription factors (TFs), RNA-binding proteins (RBPs), and microRNAs (miRNAs) [6, 8–11].

**Figure 1.**
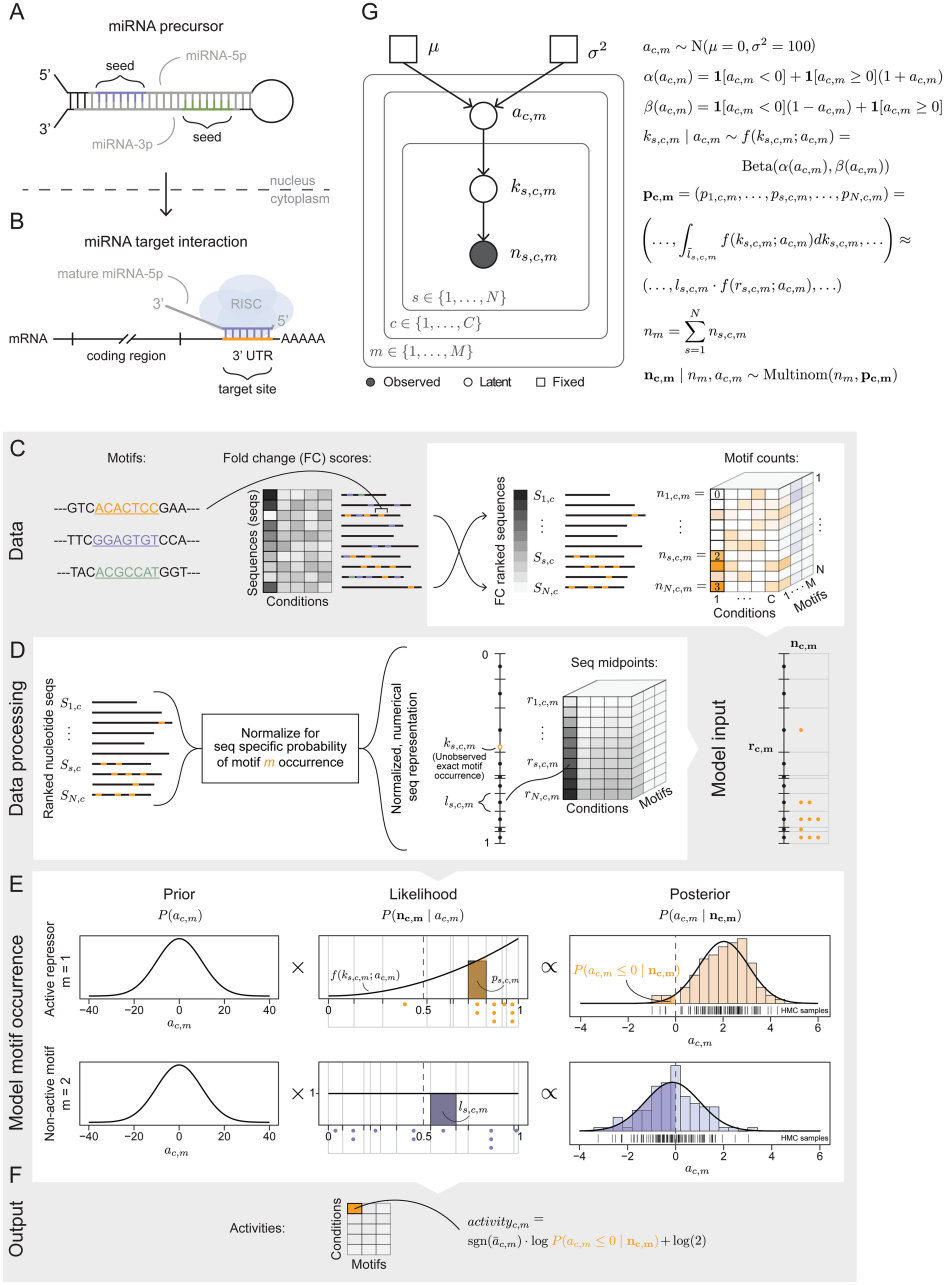
microRNA motif model and bayesReact framework. (**A**) The microRNA (miRNA) precursor with core sequence elements is highlighted. (**B**) Mature miRNA functions through RNA-induced silencing complex (RISC) association and miRNA seed- and mRNA target-site interaction. UTR = untranslated region. (**C-F**) All necessary input data (grey background), data processing, and motif modeling (white background) are depicted. (**C**) On the left is an overview of bayesReact input data, with an example including 7-mer motifs, a small simulated data set of fold-change (FC) scores across multiple conditions, and sequences with annotated motifs. To the right is depicted FC-based sequence ranking and motif distribution for a single annotated motif. $S_{s,c}$ annotates the sequence with rank $s$ in condition $c$, while $n_{s,c,m}$ the corresponding number of motif occurrences is motif $m$. (**D**) Sequence normalization is performed to adjust for sequence length and nucleotide composition bias, and is scaled such that the combined sequence length sums to one (left). $S_{s,c}$ is then represented by a numerical motif-dependent length $l_{s,c,m}$ and its rank $s$, which specifies the sequence location along the combined sequence interval [0,1]. Exact motif occurrence on the combined normalized and rescaled sequence interval $[0, 1]$ is a latent variable $k_{s,c,m}$. The position for each of the $n_{s,c,m}$ motif occurrences is approximated by the sequence midpoint $r_{s,c,m}$ (right). (**E**) Set up for inferring motif activities based on modeling motif occurrences across the ranked sequences. The motif distributions are parameterized by an underlying activity parameter $a_{c,m}$. The examples shown include an active repressor (top) and an inactive or non-functional motif (bottom). Under the likelihood, the probability of motif occurrence $p_{s,c,m}$ can be defined as an exact integral (colored) or step-function approximation (dark grey). HMC = Hamiltonian Monte Carlo. (**F**) The inferred motif activities for each motif $m$ in each condition $c$ are output. The activity (score) is the signed log posterior probability of having parameter values with the opposite sign of the posterior mean. (**G**) Graphical plate representation of the bayesReact model and its dependency structure. $a_{c,m}$ is the underlying activity parameter for motif $m$ in condition $c$; $k_{s,c,m}$ is the latent motif occurrence on the normalized ranked sequence sub-interval $\tilde{l}_{s,c,m}$, which has the length $l_{s,c,m}$; $p_{s,c,m}$ is the probability of motif $m$ occurrence on $S_{s,c}$ under $f(k_{s,c,m}; a_{c,m})$; $n_m$ the total number of motif occurrences; $\mathbf {n_{c,m}}$ the motif count distribution across all sequences. Edges indicate dependencies, squares are fixed parameters, white circles are free parameters, and the grey circle is the observed count data.

miRNA represents an intensely studied class of small non-coding RNA (ncRNA) with a length of 20-24 nts, which post-transcriptionally regulates target mRNAs by binding to their 3’ untranslated regions (UTRs) [10, 12]. Mature miRNAs originate from the 5p or 3p arm of a stem-loop precursor (Fig. 1A). In cases where both arms produce viable miRNAs, these usually differ in their seed sites and targets [13]. The miRNA-mRNA interaction primarily occurs between the miRNA seed site, usually at nucleotide positions 2-8, and a reverse-complementary target site on the mRNA (Fig. 1B) [10, 14, 15]. Mature miRNA associates with the RNA-induced silencing complex (RISC) and mainly represses target mRNA translation through transcript destabilization and degradation [10, 16]. miRNAs modulate the abundance of their target transcripts, affecting cell differentiation, proliferation, and apoptotic processes [11]. Consequently, miRNAs are also shown to be perturbed in cancer [2, 17, 18], including miR-122-5p and miR-124-3p, which are found to be down-regulated in hepatocellular carcinoma (HCC) [19–21] and glioblastomas [22, 23], respectively. Both miRNAs are highly expressed in fully differentiated cell stages, and their down-regulation may thus promote stem-like features and subsequent cancer progression [20, 23]. miRNAs can possess both oncogenic and tumor-suppressive capabilities [17], with similar trends observed for other classes of regulators [2, 24, 25].

Despite considerable progress in understanding regulatory mechanisms, elucidating their cell-level and condition-specific activities remains restricted. For instance, commonly utilized high-throughput single-cell RNA-Sequencing (scRNA-Seq) platforms use poly(dT) primers for transcript capture and amplification, subsequently excluding most circular RNAs and small non-polyadenylated transcripts [26, 27]. Meanwhile, emerging whole-transcriptome and small ncRNA-centric scRNA-Seq methods are currently limited in their throughput, sensitivity, and ability to successfully capture both long and short transcripts [28–33].

Several computational methods have been developed to indirectly predict the presence of miRNAs and the level of target depletion (Supplementary Fig. S1) [34–53]. Some methods leverage available paired miRNA-mRNA bulk expression data [34–38], including BIRTA and ActMiR. BIRTA jointly models TFs and miRNAs using a Bayesian regression framework to evaluate their switch-like behavior, and the updated biRte allows for joint regulatory network inference based on pre-defined regulator-mRNA target interactions [34, 35]. Meanwhile, ActMiR leverages the degree of negative association between miRNA and mRNA expression profiles to infer how strongly miRNAs are depleting their targets [36].

**Figure 2.**
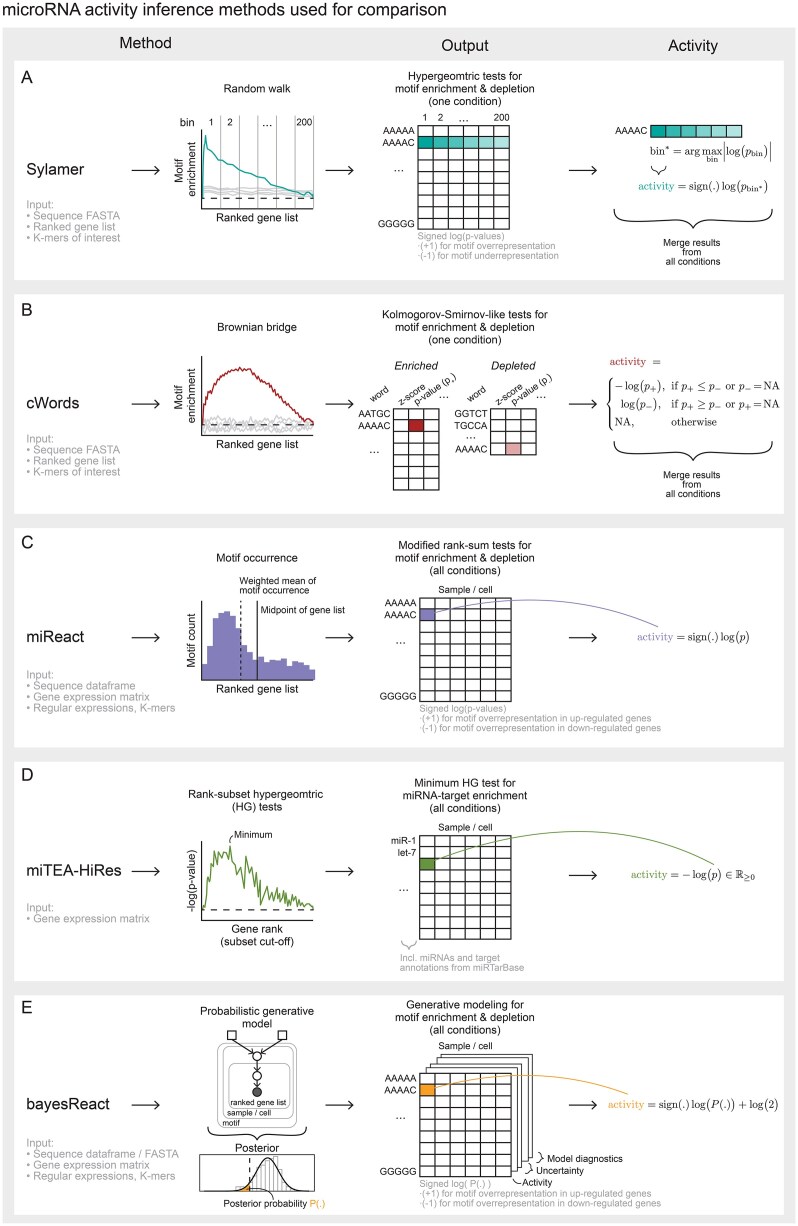
Activity inference using select methods. Simplified overviews of the methods included for comparison, highlighting their input types, general framework, output type, and how we compute the activity. $p$ = *P*-value. (**A**) Sylamer uses a hypergeometric test to simultaneously evaluate motif over- and under-representation in parts of a ranked gene list. The activity is defined as the maximal signed $\log (\mathrm{p-value)}$ for a given motif across all bins (set of adjacent sequences). The number of bins is user-defined, and we used 200 for this study. (**B**) cWords uses a Brownian bridge null model for the running-sum motif enrichment and evaluates the observed motif deviations through a Kolmogorov-Smirnov-like test. Two tests are performed for motif enrichment ($p_+$) and depletion ($p_-$). NAs represent non-significant motifs, which are omitted in the cWords output. (**C**) miReact performs a modified Wilcoxon rank-sum test to evaluate the deviation of the weighted mean of observed motif occurrences from the mid-point (expected value under a uniform null model). The activity is defined as the $\log (\mathrm{p-value)}$, with the sign depending on the mean motif occurrence being located in the up- or down-regulated portion of the ranked gene list. (**D**) miTEA-HiRes performs a minimum hypergeometric test using each gene rank as a cut-off, instead of bins, and evaluates motif enrichment at each cut-off using a one-sided hypergeometric test. The activity is then defined by the minimum *P*-value, with miTEA-HiRes only considering motif enrichment and not depletion. (**E**) bayesReact is a fully Bayesian model that leverages MCMC sampling to obtain posterior probabilities and uncertainty estimates for motif activities.

While expression measures transcript abundance, the activity provides a relative measure of the degree to which a regulator acts on its targets. Unsupervised methods, which generalize beyond miRNAs, leverage the known relationships between regulators and their targets to estimate activities. Motif-based and gene-set enrichment analysis-inspired methods offer a continuous measure of activity based on motif occurrences in experimentally ranked gene lists (Fig. 2) [39–48]. For miRNAs, a shift in target-site-containing genes towards the lowly abundant end of the list indicates active miRNA presence. This approach was first implemented in Sylamer for RNA-Seq data, which uses a hypergeometric statistic to evaluate over- and underrepresentation of simple nucleotide strings across ranked gene lists (Fig. 2A) [41, 42]. Meanwhile, cWords defines a Brownian bridge over a ranked gene list and evaluates the significance of its maximal value (Fig. 2B) [44, 54]. Inspired by and extending these methods, we developed miReact [45, 46], which consists of two steps: First, biases from the gene-specific nucleotide composition and sequence length are adjusted for. Second, the given motif’s correlation with gene ranks is evaluated using a modified Wilcoxon-Rank Sum test (Fig. 2C), which performed better compared to previous methods [46]. Notably, miReact also enables the evaluation of complex regular expressions, and the method has been shown to capture expected miRNA activities at the single-cell level. For spatial transcriptomics data, miTEA-HiRes recently showed potential for capturing miRNA activities, where miRNA target genes are defined using miRTarBase and their distribution evaluated using a minimum hypergeometric test (Fig. 2D) [47, 48]. All the methods share that their activity scores are based on p-values, comparing the observed data with a null expectation. Current methods do not explicitly model the underlying generative process driving expression-ranked motif distribution, preventing them from modeling uncertainty. Consequently, the methods do not easily extend to more complex settings, e.g., accounting for additional features such as target efficiency and integrating multiple data layers relevant to multi-omics analysis.

Here, we propose a generative process for motif occurrence across ranked gene lists, which we use to model motif activities and uncertainties by implementing a scalable probabilistic model in a user-friendly R package named bayesReact (BAYESian modeling of Regular Expression ACTivity; Figs 1 and 2E). The method is demonstrated by estimating the miRNA activities from both bulk and single-cell expression data, and is found to perform better on sparse data than previous methods. bayesReact permits general regulatory motif activity inference and evaluation of any regular expression; null model comparison using Bayes factors (BFs); computation of credible intervals (CIs); data simulation; and further model extensions, e.g., accounting for sequence rank uncertainty, target efficiency, or pseudo-time. The model is implemented in STAN, and Markov chain Monte Carlo (MCMC) sampling, or, optionally, Laplace’s approximation, is used for posterior approximation.

## Materials and methods

### Data collection and pre-processing

#### Data collection

Bulk expression data with matched mRNA and miRNA samples were obtained from The Cancer Genome Atlas (TCGA; *n* = 9640) [55]. The mRNA data were retrieved from the Recount3 project [56], and the miRNA isoform data were extracted from the GDC data portal [57]. The mature miRNA read counts were evaluated separately for their 5p or 3p origins, and precursors were omitted. The miRNA expression was normalized for library size using transcripts per million (TPM) values. The final data constitute 18 559 protein-coding genes and 2450 miRNAs, sharing 1941 unique seed sites, expressed in primary tumor samples divided into 32 cancer types from 27 distinct tissues (Supplementary Table S1). To mimic scRNA-Seq read sparsity, we generated ten semi-synthetic read count matrices with different levels of down-sampling. The matrices were created by sampling mRNAs with probabilities proportional to their observed reads per kilobase of transcript per million mapped reads (RPKM) values. The RPKM values adjust for transcript length and library size. The mRNA expression was then defined as its sampling count.

Paired mRNA and miRNA expression was also obtained from healthy samples (*n* = 15 398) covering 31 different tissue types available through the Genotype-Tissue Expression (GTEx) project (portal accessed 24/04/2025) [58, 59]. We extracted 438 miRNAs, sharing 366 target sites, by matching the RNAcentral (URS) v. 19 identifiers from the small RNA-Seq GTEx v. 10 data with miRBase v. 22 [13, 60]. We retained all miRNAs (*n* = 438) and protein-coding genes (*n* = 18 630) expressed in at least one sample. The mature miRNA expression was considered separately for 5p and 3p origins, and normalized TPM values were used, similar to the TCGA data.

We obtained a unique whole-transcriptome scRNA-Seq dataset from Isakova et al. [28], generated using the Smart-seq-total protocol. The dataset consists of 913 cells differentiated from primed mouse embryonic stem cells (mESCs).

The cells were extracted at four different time points, with 18 900 genes expressed. We processed and annotated the data using the workflow provided by Isakova et al. [28]. miRNA entries were extracted from the whole transcriptome expression matrix, which contains the combined expression of the 5p and 3p arms according to the genomic origin of the stem-loop precursor. The miRNAs were annotated using miRBase [13] and assigned the 5p or 3p target site based on the mean correlation between expression and inferred activities from all methods considered.

Similarly, we obtained paired expression profiles from Li et al., who developed the PSCSR-seq V2 protocol to perform parallel mRNA and small RNA-Seq from individual cells [61]. Here, we obtained two datasets comprising 9403 cells from four mouse lung tissue biopsies (mice aged 2, 3, 5, and 30 months) and 2310 cells from four human cell lines (HeLa, A549, K562, and 293T). The mouse lung data comprise 16 878 protein-coding genes and 443 miRNAs (separated by 5p and 3p origin) expressed in at least one cell, whereas the human cell lines contain 17 300 genes and 731 miRNAs. The miRNAs share 357 and 606 unique target sites, respectively.

Finally, we acquired a single-cell atlas of the developing mouse spinal cord created by Delile et al. [62]. They produced scRNA-Seq data using the droplet-based 10x Genomics Chromium system, performing high-throughput sequencing of poly-adenylated transcripts. Cells were extracted from mice spinal cords at five time points from embryonic day (E) 9.5 - 13.5. We retrieved the normalized unique molecular identifier (UMI) expression count matrix, which has been normalized by subsampling the cell libraries to ensure they have the same size [62]. We also retrieved cell annotations, including cell type classifications and t-SNE coordinates. Genes expressed in fewer than three cells and cells with less than 500 genes expressed or $> 6\%$ mitochondrial gene count were removed. This resulted in 38 976 cells with 19 686 expressed genes retained for analysis.

When normalized miRNA expression or read data (BAM files) were not readily available, we used log2 pseudo-TPM normalization (defined in the section below), which was the case for all single-cell datasets. $\mathrm{Log}_2$ pseudo-TPM and $\mathrm{log}_2$ TPM are used interchangeably for simplicity.

We extracted all human 3’ UTR sequences as provided by GENCODE v. 32 (TCGA and human cell lines), v. 39 (GTEx), and mouse sequences from GENCODE M23 [63]. The sequences were filtered to include instances with a length between 20 and 10 000 nts, and only the longest 3’ UTR isoform for each protein-coding gene was retained. We represent RNA sequences, transcripts, and motifs by their complementary DNA (cDNA), corresponding to replacing uracil (U) with thymine (T).

miRBase v. 22 was used for all miRNA annotations and to extract seed sites [13]. However, results from the miRBase annotations were also compared with the MirGeneDB reference database, which annotates high-confidence miRNAs using manual curation [64]. We matched miRNAs from MirGeneDB 3.0 with miRBase v. 22 using their MIMAT identifiers. Seed sites were defined as positions 2–8 for mature miRNA sequences provided by both databases. If multiple matches were present between the two databases for a given miRNA based on the MIMAT identifier, we prioritized instances where the databases agreed on the seed site, resulting in the miRBase entries being matched to at most one entry from MirGeneDB.

#### Data pre-processing

The input data for bayesReact consists of three data types: first, a numerical experimental readout such as read counts from an RNA-Seq experiment; second, a list of sequences; third, a set of motifs present in a subset of the sequences and that are expected to regulate the readout when present. Here, we use miRNA-guided depletion of target transcripts to evaluate the performance of bayesReact. The readouts then represent gene expression, the sequences are 3’ UTRs, and the motifs are miRNA target sites (Supplementary Fig. S2).

##### Expression data

The initial gene expression matrix can be provided with normalized or raw counts, which are subsequently re-scaled and $\log _2$-transformed using the function bayesReact::norm_scale_seq() (Supplementary Fig. S3). For raw data, the function normalizes the expression data using a pseudo-library size of summed read counts in each condition (cell, sample, or other experimental condition), thereby scaling the expression to sum to one. Each entry is then multiplied by the median expression of the condition and $\log _2$-transformed. This normalization procedure, termed $\log _2$ pseudo-TPM, was used on all mRNA count data.

##### Sequence ranking

The sequences were matched to the gene identifiers of the expression matrix, and expression levels were used as proxies for sequence abundances. Fold-change (FC) scores were calculated as the log-transformed expression of a gene $i$ under condition, $c$ subtracted the gene expression from a control setting. As a default, we represent the control setting by a pseudo-normal condition due to the frequent lack of control samples, e.g., lack of healthy tissue samples matching tumor biopsies. The pseudo-normal condition is defined by the median gene expressions across all conditions in a dataset; however, a user-defined control setting can also be provided. The FC-score corresponds to a measure of relative up- and down-regulation of a sequence in a given condition relative to the control. Consequently, tissue-specific expression patterns are assumed to drive deviations from the pseudo-normal for the TCGA and GTEx data, while temporal patterns are also expected to produce deviations for the developmental mouse data. The FC-scores were used to perform condition-specific sequence ranking, and sequences are ranked in decreasing order (Fig. 1C). The use of FC-scores has previously been demonstrated [39, 41, 44, 46] and ensures the extreme ends of the ranked sequence lists are not populated with housekeeping genes and genes minimally expressed in a given dataset.

##### Motif probabilities

A motif refers to any regular expression (RE) on the alphabet $\lbrace A, T, G, C\rbrace$. Here, we focus on the set of all 7-mers ($M$ = 16 384), of which a subset are miRNA targets.

The sequence-specific probability (SSP) of observing motif $m$ at least once in a random sequence with length and nucleotide composition given by sequence $i$ (from gene $i$) was computed using the Regmex package [46]. Briefly, stochastic motif generation is modeled by an absorbing Markov chain, where nucleotides are drawn with probability equal to their frequency in sequence $i$ upon transition across the state-space induced by the motif RE.

### Modeling motif activity

We developed a Bayesian method, bayesReact, to evaluate the association of a motif with the gene expression pattern and, hence, the ranking of sequences for a given condition. bayesReact models the activity of functional motifs based on motif occurrence across FC-ranked sequences. Using a generative, probabilistic approach facilitates model extensions and allows the uncertainty of activity estimates to be quantified.

#### Data representation and null expectation

Let $\lbrace S_{1,c}, S_{2,c}, \ldots , S_{s,c}, \ldots , S_{N,c}\rbrace$ be a set of FC-score ranked sequences, where index $s \in \lbrace 1, \ldots ,N\rbrace$ defines the rank, $c \in \lbrace 1, \ldots ,C\rbrace$ the condition, and let $m \in \lbrace 1, \ldots , M\rbrace$ index the set of $M$ independent motifs (Fig. 1). The ranked sequences are arranged consecutively to represent non-overlapping intervals, $\tilde{l}_{s,c,m}$, in a given condition $c$, and normalized and rescaled to have a length $|\tilde{l}_{s,c,m}| = l_{s,c,m}$ such that their joint length sums to one (Fig. 1D; Supplementary Methods). The normalization accounts for the probability of observing $m$ given the sequence length and nucleotide composition of $S_{s,c}$, and $l_{s,c,m}$ is proportional to the expected number of motif occurrences in each sequence. Consequently, the distribution of exact motif occurrence $k_{s,c,m}$ across the ranked sequences, represented as an interval [0,1], is expected to be uniform if driven solely by the sequence contexts. In addition, the number of motif occurrences $\mathbf {n_{c,m}} = (n_{1,c,m},\ldots , n_{s,c,m}, \ldots , n_{N,c,m})$ in each sequence is expected to depend only on the total number of motif occurrences $n_{m} = \sum \limits _{s=1}^{N} n_{s,c,m}$, constant across all conditions, and the length of the normalized sequence interval. Specifically, the null model assumes no association between relative sequence abundance (rank) and motif occurrence:


(1) \begin{eqnarray*}
k_{s,c,m} &\sim & \mathrm{Unif}(0, 1) \\\mathbf {n_{c,m}} \mid n_{m} &\sim & \mathrm{Multinom}(n_m, \mathbf {l_{c,m}}).
\end{eqnarray*}

A non-functional motif or inactive motif-based regulatory mechanism is expected to produce evenly distributed motif occurrences, and the motif count in a sequence will only depend on the length $l_{s,c,m}$ of its sub-interval.

#### Modeling motif occurrence and the number of motifs across ranked sequences

Deviations from the null model described above can, for example, arise when a microRNA acts on a set of target transcripts. The depleted target sequences will be systematically skewed toward the end of the ranked sequence list (Fig. 1E). This signal can be captured by letting the motif occurrence be distributed according to a flexible beta distribution with support on $[0, 1]$, accounting for sequence ranking (position along $[0, 1]$). The uniform null model then becomes a special case, $\mathrm{Unif}(0, 1) \overset{d}{=} \mathrm{Beta}(1,1)$, and we have that:


(2) \begin{eqnarray*}
k_{s,c,m} \mid a_{c,m} &\sim & \mathrm{Beta}(\alpha (a_{c,m}), \beta (a_{c,m})) \\\alpha (a_{c,m}) &=& \mathbf {1}[a_{c,m} < 0] + \mathbf {1}[a_{c,m} \ge 0](1 + a_{c,m}) \\\beta (a_{c,m}) &=& \mathbf {1}[a_{c,m} < 0](1 - a_{c,m}) + \mathbf {1}[a_{c,m} \ge 0],
\end{eqnarray*}

where $\mathbf {1}[.]$ is an indicator function. The beta shape parameters are transformations of an underlying activity parameter $a_{c,m} \in \mathbb {R}$. The activity parameter has a clean interpretation concerning motif distribution; $a_{c,m} < 0$ entails motif clustering at the beginning of the combined sequence interval constituting sequences with high relative gene expression (FC-scores); $a_{c,m} = 0$ corresponds to the null model; and $a_{c,m} > 0$ implies motif over-representation at the end of $[0, 1]$ for sequences with low relative abundance (Fig. 1E).

Under the beta distribution, which has the probability density function (PDF) $f(k_{s,c,m}; a_{c,m})$, we can describe the probability of motif occurrence $\mathbf {p_{c,m}} = (p_{1,c,m}, \ldots , p_{s,c,m}, \ldots , p_{N,c,m})$ in each sequence as the area under $f(k_{s,c,m}; a_{c,m})$ for its sub-interval $\tilde{l}_{s,c,m}$ (Fig. 1E; Supplementary methods). The number of motif occurrences on each sequence can subsequently be described by a multinomial distribution:


(3) \begin{eqnarray*}
\mathbf {n_{c,m}} \mid n_{m}, a_{c,m} &\sim \mathrm{Multinom}(n_m, \mathbf {p_{c,m}}).
\end{eqnarray*}

Although $p_{s,c,m}$ can be found by evaluating an integral for each sequence sub-interval, this is computationally intensive for large data sizes. Instead, we approximate the beta distribution with a step-function, where $p_{s,c,m} \approx l_{s,c,m} \cdot f(r_{s,c,m}; a_{c,m})$ and $r_{s,c,m}$ is the mid-point of the normalized sequence interval for $S_{s,c,m}$ (Fig. 1D-E; Supplementary methods). Increasing the total number of sequences $N$, leads to a finer partitioning of $[0, 1]$, e.g., using all human 3’ UTRs divides the combined sequence interval into $\sim$20K sub-intervals.

Subsequently, the joint probability of the motif counts across all sequences, conditional on the underlying activity parameter, can be approximated by the following parameterization of the multinomial probability mass function (PMF):


(4) \begin{eqnarray*}
&& P(\mathbf {n_{c,m}} \mid a_{c,m}) \propto \prod _{s = 1}^{N} {p_{s,c,m}}^{n_{s,c,m}} \\&& \approx \prod _{s = 1}^{N} \left(l_{s,c,m} \cdot f(r_{s,c,m}; a_{c,m})\right)^{n_{s,c,m}}.
\end{eqnarray*}

An additional benefit of approximating $\mathbf {p_{c,m}}$ is the ability to pre-compute part of the log-likelihood ($\log P(\mathbf {n_{c,m}} \mid a_{c,m})$), allowing for further computational speed-up (see Supplementary Methods).

Finally, we place an uninformative prior on the activity parameter centered at zero: $a_{c,m} \sim \mathrm{N}(\mu = 0, \sigma ^2 = 100)$. By establishing the log-likelihood and prior distribution of $a_{c,m}$, it is possible to explore the marginal posterior density of interest after marginalizing the latent variable $k_{s,c,m}$:


(5) \begin{eqnarray*}
\log P(a_{c,m} \mid \mathbf {n_{c,m}}) &\propto \log P(\mathbf {n_{c,m}} \mid a_{c,m}) + \log P(a_{c,m}).
\end{eqnarray*}

MCMC sampling is used to sample from the unnormalized posterior (the stationary target distribution of interest, referred to interchangeably as the posterior). After an approximation of the posterior is obtained, we find the activity score of motif $m$ in condition $c$ based on the signed posterior tail probabilities (corresponding to the probability of no motif activity after observing the data; Fig. 1F):


(6) \begin{eqnarray*}
activity_{c,m} = \left\lbrace \begin{array}{@{}l@{\quad }l@{}}\mathrm{sgn}(\bar{a}_{c,m}) \cdot \\\log P(a_{c,m} \le 0 \mid \mathbf {n_{c,m}}) + \log (2), & \bar{a}_{c,m} \ge 0 \\\mathrm{sgn}(\bar{a}_{c,m}) \cdot \\\log P(a_{c,m} \ge 0 \mid \mathbf {n_{c,m}}) + \log (2), & \bar{a}_{c,m} < 0, \end{array}\right.
\end{eqnarray*}

where $\bar{a}_{c,m}$ is the posterior mean of the density $P(a_{c,m} \mid \mathbf {n_{c,m}})$. The activity score represents the two posterior tail-probabilities and to avoid discontinuity, all values have been added a constant $\log (2)$.

The use of MCMC for activity inference permits modeling flexibility. However, even for a large number of MCMC samples, the tails of the posterior are not efficiently characterized, and normal approximations of the MCMC samples are used instead, motivated by the Bernstein-von Mises theorem (asymptotic normality of Bayesian posteriors for large $n_m$, see, e.g., [65]). The approximation avoids issues with $P(a_{c,m} \le 0 \mid \mathbf {n_{c,m}})$ evaluating to zero, and we maintain resolution for highly active motifs.

Establishing a generative model enables exploration of the underlying processes driving motif occurrence and clustering across experimentally ranked sequences of interest (Fig. 1G). Here, motif occurrence is described under a beta distribution and controlled by an underlying activity parameter whose prior and posterior densities are both approximately normal. The activity measure captures how skewed the motif distribution is along the ranked sequences and, equivalently, the effect of a regulator on its target motifs.

#### Bayes factor for null model comparison

A motif occurrence can either be non-functional (generated under a uniform distribution) or functional (generated under a beta distribution). In general, we expect occurrences of $m$ on the set of sequences to be a combination of true functional motif occurrences (a subset of which is targeted in a given condition $c$) and non-functional false positives. The null ($M_0$; $a_{c,m} = 0$) and alternative ($M_1$) models can be compared based on their marginal log-likelihoods using the Bayes factor (BF):


(7) \begin{eqnarray*}
&& \log BF_{10} \\&=& \log \int _{a_{c,m}} P(\mathbf {n_{c,m}} \mid M_1, a_{c,m})P(a_{c,m} \mid M_1) da_{c,m} \\&-& \log P(\mathbf {n_{c,m}} \mid M_0) \\&=& \log P(\mathbf {n_{c,m}} \mid M_1) - \log P(\mathbf {n_{c,m}} \mid M_0).
\end{eqnarray*}

Due to the direct comparison of marginal log-likelihoods, the normalizing constants cannot be disregarded and are instead approximated using bridge sampling [66]. The BF is useful to evaluate how well $M_1$describes the motif observations compared to $M_0$, particularly for conditions with activities deviating slightly from zero.

#### Flexible two-parameter beta model

A more flexible model was also implemented and evaluated, where the two beta parameters $\alpha _{c,m}$ and $\beta _{c,m}$ are freely variable instead of transformations of $a_{c,m}$. Here, the activity is based on the posterior probability of the mean value $\tau _{c,m}$ of the motif occurrence density being larger or smaller than the midpoint of the combined sequence interval:


(8) \begin{eqnarray*}
activity_{c,m} = \left\lbrace \begin{array}{@{}l@{\quad }l@{}}\mathrm{sgn}(\bar{\tau }_{c,m} - 0.5) \cdot \\\log P(\tau _{c,m} \le 0.5 \mid \mathbf {n_{c,m}}) + \log (2), & \bar{\tau }_{c,m} \ge 0.5 \\\mathrm{sgn}(\bar{\tau }_{c,m} - 0.5) \cdot \\\log P(\tau _{c,m} \ge 0.5 \mid \mathbf {n_{c,m}}) + \log (2), & \bar{\tau }_{c,m} < 0.5, \end{array}\right.
\end{eqnarray*}

where $\bar{\tau }_{c,m}$ is the mean value of the marginal posterior density $P(\tau _{c,m} \mid \mathbf {n_{c,m}})$ (Supplementary Methods). We refer to the two models as bayesReact and bayesReact_2p_, respectively.

### bayesReact implementation

bayesReact is an R package implemented in R, Bash, and STAN (through RSTAN [67]), allowing for user-defined evaluation of motif activities across experimentally ranked sequences. The package constitutes three primary modules: Data pre-processing, modeling motif activities and posterior approximation, and parallelization (Supplementary Fig. S3). STAN’s Hamiltonian Monte Carlo (HMC) algorithm is used for MCMC sampling [68], and bayesReact can return the complete set of posterior samples, summary statistics such as the posterior mean of $a_{c,m}$, credible intervals (CIs) and model diagnostics, or simply return the activity scores directly. To avoid large influences of individual sequences on the distribution of motif occurrences, a user-defined threshold can be placed on $l_{s,c,m}$ and $n_{s,c,m}$, with a default of $10^{-6}$ and 2, respectively. The user can also specify the number of MCMC chains, the number of MCMC samples, and whether to compute BFs. In addition to MCMC sampling (default), Laplace approximation of the posterior is also possible, providing faster activity inference at the expense of less accurate uncertainty estimates and less comprehensive model diagnostics.

### Method comparisons

The bayesReact method was compared against the existing tools miReact, Sylamer, cWords, and miTEA-HiRes primarily on the TCGA and single-cell mESCs data. An overview of the methods and how the activity is defined can be found in Fig. 2 and Supplementary Fig. S1. All motif-based methods (bayesReact, miReact, Sylamer, cWords) were run for all 7-mers on the same gene rankings, using the same-order Markov model to account for random motif occurrence as a product of nucleotide frequencies and sequence length. miTEA-HiRes was provided with the gene expression counts directly, and it performs inference for the subset of miRNAs with target information in miRTarBase. The method produces strictly positive-valued activity scores because it only evaluates the overrepresentation of miRNA targets at one end of a ranked gene list.

The methods were parallelized on 20 sample data partitions to reduce overall running time, except miTEA-HiRes, which has an internal cross-sample (and within-sample) normalization step. Furthermore, Sylamer and cWords were initially designed to evaluate a single case-control condition at a time, and we subsequently designed wrapper scripts to scale the methods to multiple conditions simultaneously.

The resource consumption of all methods was evaluated in terms of overall running time (from job submission until completion) and maximum memory usage (summed across data partitions). Additionally, the parallelized methods were evaluated based on total elapsed time and maximum memory usage per partition.

### Permutation test on miRNA motif assignment

We assessed whether the observed correlation between miRNA target motif activity and miRNA expression could arise by random chance through permutation tests on the TCGA and Smart-seq-total data. This was possible since we ran bayesReact for all 7-mer motifs. Specifically, we generated 10 000 random miRNA motif subsets by randomly assigning a 7-mer activity profile to a miRNA without replacement, using the set of 7-mers not defined as miRNA targets in miRBase or MirGeneDB (*n*_HSA_ = 14 405, *n*_MMU_ = 14 789). Summary statistics were then computed for each random motif subset, including the mean and 90th percentile of the Pearson and Spearman correlations. The true miRNA target motif subset was compared to the resulting empirical null distributions and considered relative to the 99th percentile (extreme tail). Values larger than the 99th percentile are considered extremely surprising (significant).

## Results

### Model performance on bulk pan-cancer data

Initial model evaluation on the TCGA data showed a rapid convergence rate, with the MCMC sampler usually converging on the posterior within a hundred iterations (Supplementary Fig. S4). We subsequently set the default number of MCMC iterations to 3000 and the warm-up period of 500 iterations to be discarded. Running three independent MCMC chains for all 7-mer motifs across the pan-cancer primary tumor samples shows reliable convergence ($\hat{R} < 1.05$) for $99.99\%$ of the activity parameters $a_{c,m}$ ( $11\cdot 10^3$ of $158\cdot 10^6$ parameters have $\hat{R} > 1.05$; Supplementary Fig. S4A). Additionally, the effective sample sizes (ESSs) tend to be large, indicating low autocorrelation and efficient exploration of the posterior ($99.85\%$ of the activity parameters have an ESS of more than 1000; Supplementary Fig. S4B). Rerunning bayesReact and increasing the number of iterations showed improved diagnostics for individual cases.

Comparable diagnostics are also found for the more flexible bayesReact_2p_ model ($100\%$ of $\hat{R} < 1.05$ and $ESS > 1,000$; Supplementary Fig. S4C).

Comprehensive model evaluation was performed for individual $a_{c,m}$ (Supplementary Fig. S4D-G), with prior and posterior predictive checks showing vastly improved agreement between observed cumulative motif distributions and simulated motif data under the posterior predictive distribution compared to the prior predictive distribution (Supplementary Fig. S4E, G). We also evaluated the variability in activity estimation, a product of stochastic MCMC sampling. This was done by conducting 1000 bayesReact repetitions for the two motifs ‘ACACTCC’ (miR-122-5p target) and ‘GTGCCTT’ (miR-124-3p target) across all TCGA samples. Each repetition was summarized by the activity correlation with the observed miRNA expression data. Encouragingly, the Pearson correlation showed low variability, and the coefficients range from $0.824 - 0.828$ and $0.456 - 0.464$, respectively, and the median and mean values coincide in both instances (Supplementary Fig. S4H). Equivalent results are observed for bayesReact_2p_.

Notably, a significant speed-up of the bayesReact model is achieved through approximation of the likelihood with a step-function (eq. 4). We observe $\mathtt {>>}278$-fold reduction in run time for evaluating a motif across the full TCGA data, while retaining agreement in activity estimates (Supplementary Fig. S5A–C). Due to the approx. Gaussian posterior, additional computational speed-up is also possible through Laplace approximation instead of exhaustive MCMC sampling (mean and median Pearson correlation $> 0.97$ between resulting activities; Supplementary Fig. S5D, E). bayesReact and bayesReact_2p_ also produce similar activity estimates and correlation to the observed miRNA expression data (Supplementary Fig. S5E, F). Overall, bayesReact obtains a mean activity score of 0.03 and standard deviation (sd) of 3.12 across all 7-mer motifs and tumor samples. Similarly, bayesReact_2p_ provides a mean of 0.02 (sd = 3.29). The low mean activity is consistent with the majority of 7-mers expected to be non-functional, and that many functional regulatory motifs are tissue and cancer-type-specific. Due to the comparable results between the two models, we proceed using the model with the fewest free parameters as the core of bayesReact.

Finally, we compared the resource consumption of bayesReact with the existing methods miReact, Sylamer, cWords, and miTEA-HiRes. With the exception of the highly efficient Sylamer, bayesReact had comparable running times and max memory usage as that of the other methods, even though it is a fully probabilistic generative model (Supplementary Fig. S5G–J).

#### microRNA activity in cancer

**Figure 3.**
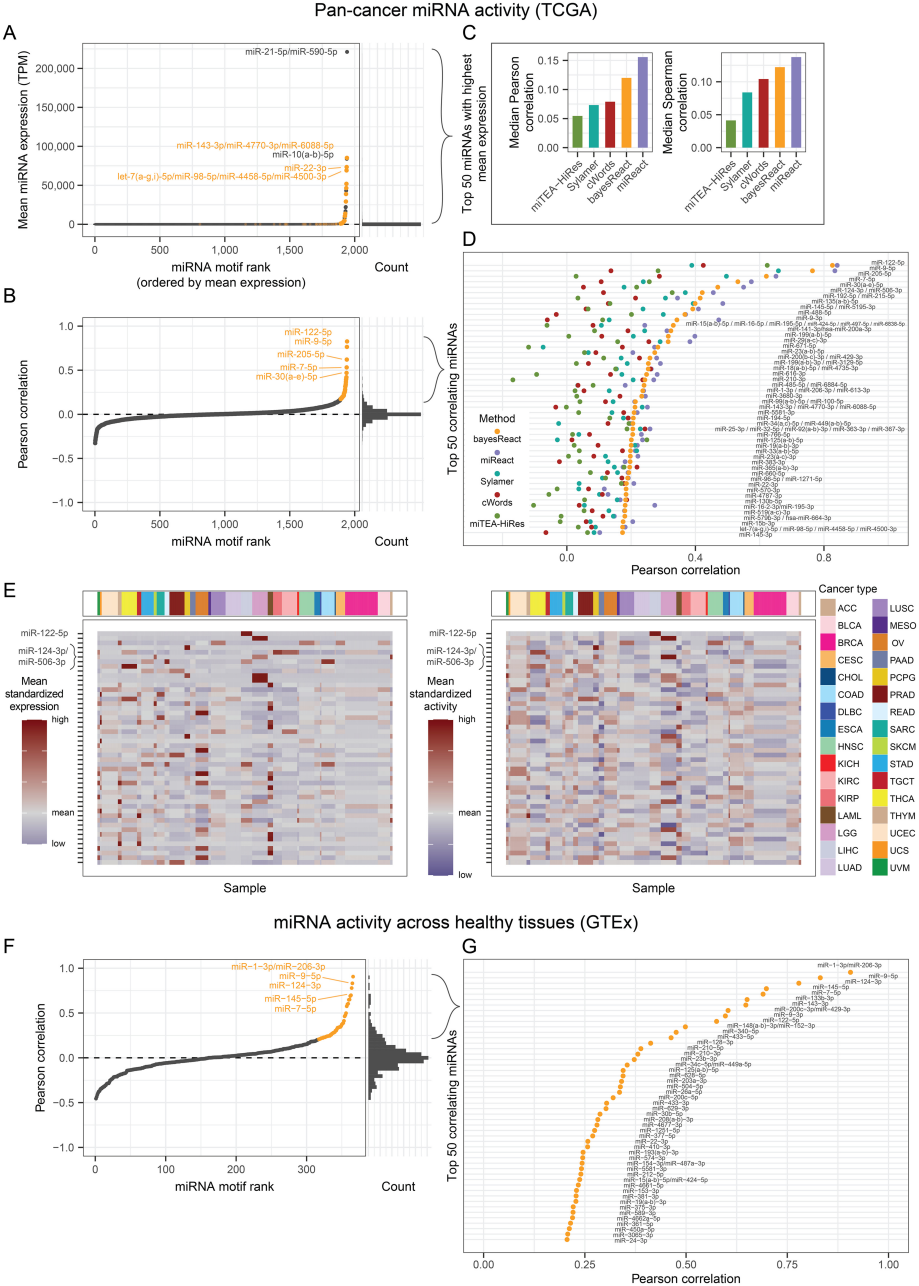
Pan-cancer microRNA activity and expression overview. (**A**) miRNAs ranked by average expression across all TCGA samples (left). Depicted are 2450 miRNAs with expression collapsed by their shared target sites (*n* = 1941). The top 50 correlating miRNAs from panel B are highlighted in orange, and the top five are annotated. On the right is depicted the corresponding histogram with 50 bins. TPM = transcripts per million. (**B**) Pearson correlation between miRNA expression and activity across all primary tumor samples (left). The miRNAs and their target motifs are subsequently ranked by the correlation. The top 50 miRNAs with the highest correlation coefficients are highlighted (orange), and the top five are annotated. On the right is depicted the corresponding histogram containing 50 bins. (**C**) Median correlation between expression and activity for miRNAs with the largest mean expression (*n* = 50). The miRNA activities across all TCGA samples are inferred using five different methods. (**D**) Top 50 miRNAs based on activity and expression correlation across all samples. The Pearson correlation is shown for all activity inference methods. (**E**) Heatmaps for the top 50 correlating miRNAs ordered by correlation coefficient and clustered by cancer type. The mean expression for each cancer type (left) and bayesReact activity (right) is depicted. Values are standardized for visualization purposes. (**F**) Expression (TPM values) and activity correlation for collapsed miRNAs sharing their target site (*n* = 366) across healthy tissue samples (GTEx; *n* = 15 398). The miRNAs are ranked by Pearson correlation, and the corresponding histogram containing 50 bins is depicted to the right. (**G**) The top 50 correlating miRNAs (panel F) are shown in detail.

The miRNA expression is generally small across the pan-cancer TCGA data, with an overall mean expression of 504.73 TPM (sd = 9277.21 and median = 0 TPM), and the majority of miRNAs have a mean expression across the tumor samples close to zero (Fig. 3A). Performing hierarchical clustering, with the number of clusters predefined as the number of cancer types (*n* = 32), we find that the mRNAs and miRNAs with the highest mean expression (*n* = 100) recover the cancer-type clusters to the same degree. The clustering yields adjusted rand indexes (ARIs; [69]) of 0.20 and 0.19, respectively (Supplementary Fig. S6A and B). Interestingly, the corresponding inferred miRNA activities recover the cancer clusters to the same degree, indicating a similar level of information content present to differentiate between cancer types (ARI = 0.22; Supplementary Fig. S6C; Supplementary Table S2).

While the activity of a miRNA does not directly correspond to its expression level, the two variables are still expected to be associated. An elevated cytoplasmic miRNA content can increase the degradation of its mRNA targets, while non-transcribed miRNAs are inactive [10]. We exploit this relationship to evaluate the performance of bayesReact using the correlation between the observed miRNA expression and inferred activity. The mean Pearson correlation ($\bar{\rho _{p}}$; sensitive to tissue-specific outliers) is small for the set of 2450 expressed miRNAs collapsed by their 1941 shared target motifs ($\bar{\rho _{p}} = 0.01$; Fig. 3B). This is unsurprising given the miRNAs’ overall limited expression and activity across the primary tumors, combined with the two measures differing distributions (Supplementary Fig. S7A). Similar results are also obtained using existing methods to estimate the miRNA activities ($\bar{\rho _{p}} \le 0.01$; Supplementary Fig. S7B), with the methods applied to the same input data as bayesReact for comparative analysis. Considering miRNAs with the top mean expression (n = 50; Fig. 3A), we find that miReact and bayesReact have the highest average correlation between expression and inferred activity (Fig. 3C). Unlike Sylamer, cWords, and miTEA-HiRes, miReact obtains higher correlations with bulk TCGA miRNA expression than bayesReact (Fig. 3C). However, both bayesReact and miReact tend to recover the same top-ranking miRNAs (Fig. 3B,D, Supplementary Fig. S7B) and generate similar pan-cancer activity profiles (Supplementary Fig. S8).

We generally observe the top correlating miRNAs to have a cancer-type- and tissue-specific expression and activity pattern (Fig. 3E; Supplementary Table S3). Prominent examples include miR-122-5p ($\rho _{p} = 0.83$), primarily expressed in the liver-derived tumors, as well as miR-9-5p ($\rho _{p} = 0.76$) and miR-124-3p ($\rho _{p} = 0.46$) expressed in brain tumors (Fig. 3D-E). Similar results are also observed across healthy GTEx tissue samples (n = 15 398; $\bar{\rho _{p}} = 0.03$; Fig. 3F-G, Supplementary Fig. S9). Here, bayesReact recovers activity profiles for the muscle- and heart-specific miR-1-3p/miR-206-3p ($\rho _{p} = 0.91$) as well as miR-9-5p ($\rho _{p} = 0.83$), miR-124-3p ($\rho _{p} = 0.78$), and miR-122-5p among others ($\rho _{p} = 0.58$; Supplementary Fig. S9).

##### miR-122-5p activity in liver hepatocellular carcinoma

**Figure 4.**
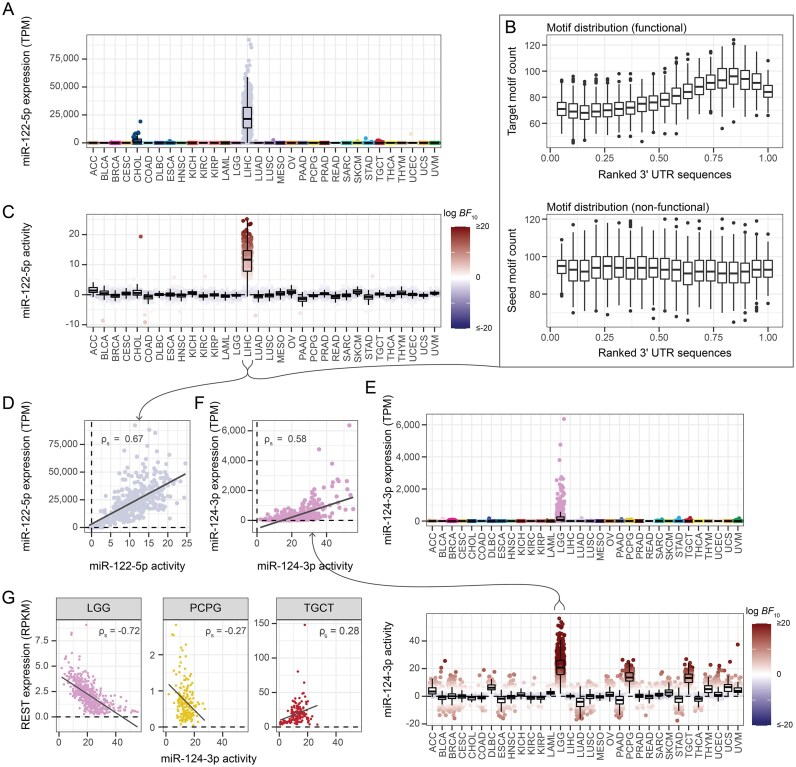
Cancer-type-specific miR-122-5p and miR-124-3p activities. (**A**) miR-122-5p expression for each primary tumor sample grouped by cancer type. TPM = transcripts per million. (**B**) miR-122-5p target site (top) and its complementary seed site (bottom) distribution across the normalized 3’ untranslated region (UTR) sequences. The combined 3’ UTR sequence interval is divided into 20 bins, and each boxplot depicts the motif count within a bin for each LIHC sample (*n* = 367). The target motif occurs 1692 times in 1526 3’ UTRs, while the seed motif occurs 1978 times in 1768 3’ UTRs. (**C**) miR-122-5p activities grouped by cancer type. Each sample is annotated with the $\log BF_{10}$ value. BF = Bayes factor. (**D**) Scatterplot of miR-122-5p activity against expression, with the Spearman correlation ($\rho _s$) annotated and linear regression line shown. (**E**) miR-124-3p expression (top) and activity (bottom) across all TCGA samples. Top: Samples are annotated by cancer type. Bottom: Samples are annotated by their $\log BF_{10}$ value. (**F**) miR-124-3p activity plotted against the observed expression, with the Spearman correlation annotated and linear regression line shown. (**G**) Association between the miR-124-3p activity and the expression of its downstream target REST in low-grade gliomas (LGG; *n* = 509; left), pheochromocytoma and paragangliomas (PCPG; *n* = 182; middle), and testicular germ cell tumors (TGCT; *n* = 139; right). Expression values are provided as reads per kilobase of transcript per million mapped reads (RPKM). Spearman correlations ($\rho _s$) and linear regression lines are visualized.

miR-122-5p, which has the highest association between pan-cancer expression and activity, is known to be highly expressed in liver tissue [70] and is predominantly expressed in the liver hepatocellular carcinoma samples (LIHC; *n* = 367; Fig. 4A), making it a good candidate for case-specific evaluation of bayesReact. The expressed miR-122-5p is expected to deplete its target transcripts actively, and concordantly, we observe a clear association between the occurrence of the miR-122-5p target motif in the 3’ UTR of a gene and its relative expression (position along the FC-ranked 3’ UTR sequence interval; Fig. 4B). In contrast, the complementary miR-122-5p seed site distribution across the FC-ranked sequences is uniform, as it does not represent a regulatory motif in the 3’ UTRs (Fig. 4B). Providing the motif data ($\mathbf {n_{c,m}}$) to bayesReact, the marginal posteriors for the miR-122-5p activity parameters are obtained, which we summarized by their mean and 99% CI. We find that the credible intervals mostly overlap zero for LIHC samples with low miRNA expression (Supplementary Fig. S10A). Subsequently, the posterior tail probabilities (see eq. 6) are larger in liver cancer compared to other cancer types (Supplementary Fig. S10B), resulting in a LIHC-specific miR-122-5p activity. Furthermore, TCGA samples with activity scores close to zero also tend to have $\log BF_{10} \le 0$ (Fig. 4C). Concludingly, bayesReact efficiently recovers miR-122-5p activities, which agree with the observed miRNA expression and known liver-specific function. The results are consistent with previous miReact findings [46] and showcase bayesReact’s extended capabilities in quantifying uncertainties for the underlying activity parameters and subsequent regulatory motifs of interest.

Considering the inferred LIHC activities, a positive Spearman correlation ($\rho _s$; robust to outliers) is observed between the miR-122-5p activity and expression ($\rho _s = 0.67$; Fig. 4D). In comparison, $\rho _s = 0.64$ for the miReact activities. In addition, the activity negatively correlates with the expression levels of the known target genes Rac1 and RhoA ($\rho _s \le -0.33$; Supplementary Fig. S10C; [71]), and a positive association is observed between the miRNA activity and the TF promoting its host gene transcription ($\rho _s = 0.42$; Supplementary Fig. S10D; [72]). The observed miR-122-5p expression showed similar associations with the expression of the target genes ($\rho _s \le -0.38$) and TF ($\rho _s = 0.39$).

##### The miR-124-3p activity negatively associates with the expression of the anti-neuronal RE1-silencing transcription factor

miR-124-3p promotes a neuronal cell fate for differentiating neuroectodermal progenitors [73, 74], and is expressed in low-grade gliomas (LGG) as well as moderately expressed in pheochromocytomas and paragangliomas (PCPG) and testicular germ-cell tumors (TGCT; Fig. 4E, Supplementary Fig. S10E). We see a positive correlation between the miR-124-3p expression and activity across all TCGA samples (Fig. 3D) and within cancer types with prominent miR-124-3p expression ($\rho _s \ge 0.26$; Fig. 4F, Supplementary Fig. S10F-G). The LGG samples showed the largest expression, activity, and subsequent association between the two ($\rho _s = 0.58$ using bayesReact and $\rho _s = 0.57$ with miReact).

miR-124-3p partake in a double-negative feedback loop by antagonizing the RE1-silencing transcription factor (REST)/Scp1 pathway through Scp1 transcript degradation [75–78]. Consistently, a negative association between miR-124-3p activity and Scp1 expression is observed in the LGG samples ($\rho _s = -0.51$, Supplementary Fig. S10H). Depletion of Scp1 is expected to destabilize the REST protein [79], which otherwise enables the suppression of neuron-specific gene transcription and promotes non-neuronal cell states [75, 79]. We observe a strong negative correlation between the inferred miR-124-3p activity and REST expression in LGG samples ($\rho _s = -0.72$; Fig. 4G), which, intriguingly, is larger than for the corresponding miRNA expression ($\rho _s = -0.32$; Supplementary Fig. S10H). A negative correlation is only observed in tumors originating from the nervous system (Fig. 4G). Interestingly, miR-9-5p, known to target the REST transcript directly [77, 80, 81], showed a smaller correlation with the REST expression ($\rho _s = -0.33$ for its activity and $\rho _s = -0.31$ based on the expression).

In addition, PTBP1 and BAF53a are also part of the regulatory REST circuit and have previously been shown to be depleted by miR-124-3p [73, 77, 78, 82]. The expression of both PTBP1 and BAF53a correlates negatively with the miR-124-3p activity in low-grade gliomas ($\rho _s = -0.44$ and $\rho _s = -0.55$, respectively) and, to a lesser degree, with the miRNA expression ($\rho _s = -0.42$ and $\rho _s = -0.40$, respectively).

#### microRNA activity inference on sparse expression data

**Figure 5.**
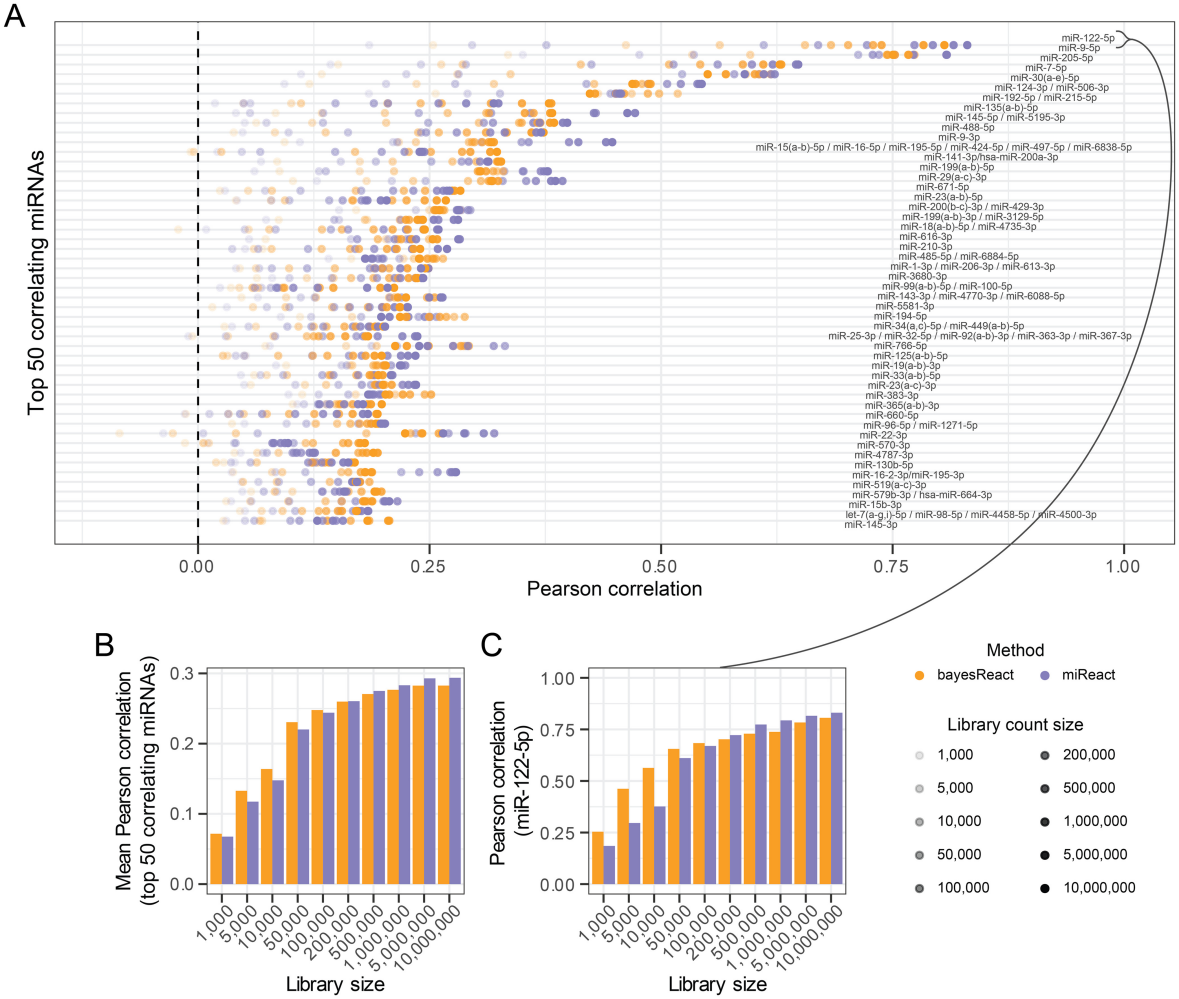
Pan-cancer miRNA activity and expression correlation for varying library sizes, created through downsampling. (**A**) Pan-cancer Pearson correlation between miRNA activity and expression for library sizes of different count sparsity. Activities were inferred through bayesReact (orange) and miReact (purple). miRNAs match Fig. 3 panel D. (**B**) Mean Pearson correlation for the top 50 correlating miRNAs at differing count sparsity. (**C**) miR-122-5p correlation for varying library size.

While miRNA expression can be recovered from small RNA-Seq protocols, it is not recovered when performing high-throughput scRNA-Seq. Activity inference thus presents a unique opportunity to indirectly investigate the cell-level miRNA activity. To evaluate the performance of bayesReact on data with similar read sparsity as scRNA-Seq data, we generated ten semi-synthetic TCGA expression matrices with varying library sizes, which were used as input for miRNA activity inference by bayesReact and miReact. In general, increased read count sparsity led to decreased activity scores and subsequent lower correlation between the miRNA activity and its expression (Fig. 5A). Focusing on the top 50 correlating miRNAs (Supplementary Table S3), bayesReact tends to retain a higher correlation score with increasing count sparsity than miReact (Fig. 5A and B). miReact recovers the activity slightly better for large library sizes ($> 500,000$ gene counts), while bayesReact outperforms miReact at count sparsities resembling single-cell levels ($< 200,000$ counts; [83–85]). For example, bayesReact tends to better differentiate between samples with and without miR-122-5p expression even for extremely sparse count data (Fig. 5C, Supplementary Fig. S11). Particularly, the medians of the miR-122-5p activity in non-LIHC cancer types are closer to zero for bayesReact compared to miReact (Supplementary Fig. S11). These results indicate improved performance over miReact on sequencing data with high zero-count content and may subsequently indicate improved performance on sparse scRNA-Seq data.

### Recovering microRNA activities at the single-cell level

#### microRNA expression and activity during stem cell differentiation

**Figure 6.**
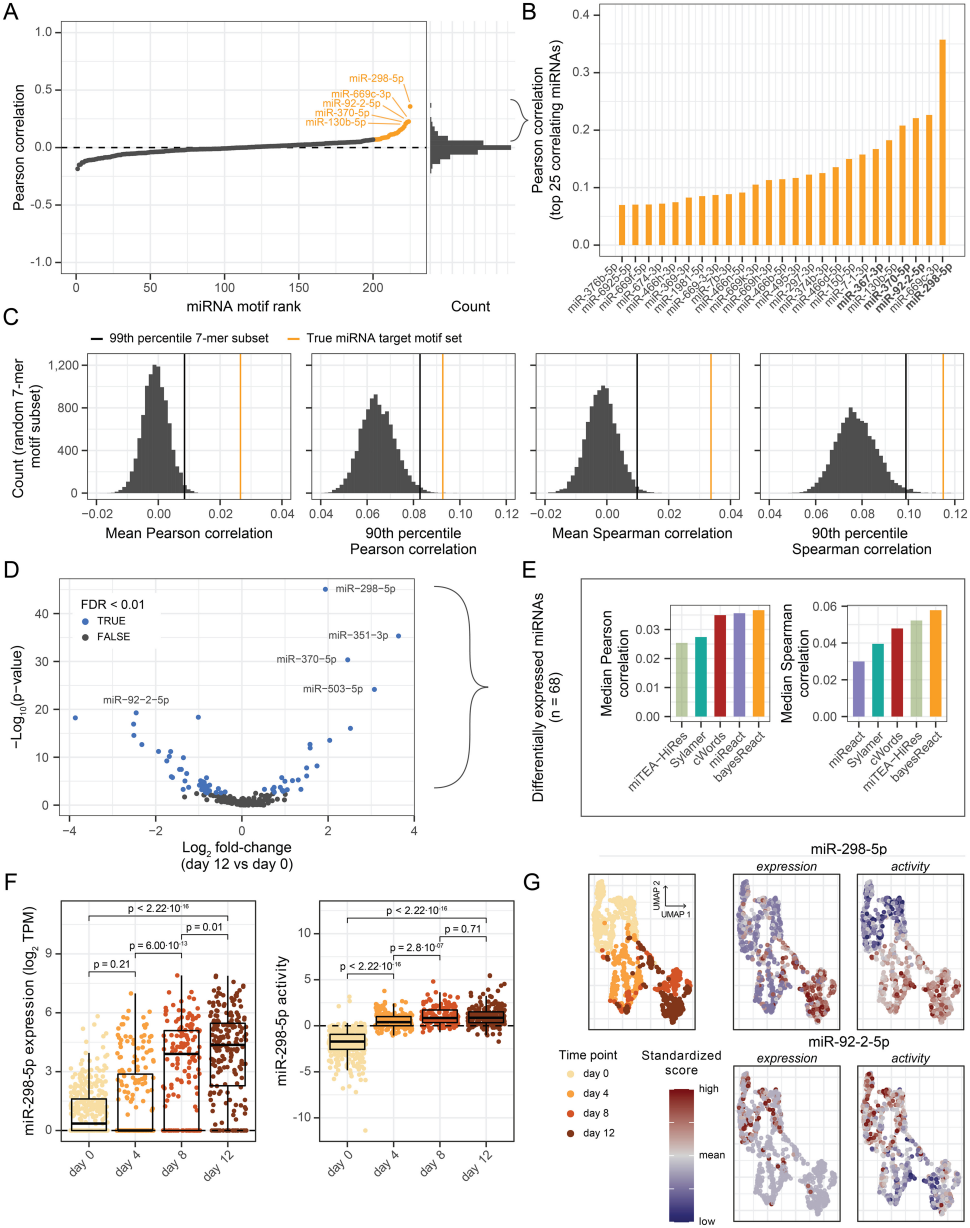
Recovering microRNA activities at the single-cell level from mouse embryonic stem cells. (**A**) Pearson correlation between miRNA activity and expression across all cells (*n* = 913), ranked by their correlation coefficient. miRNA activities were obtained using bayesReact. The corresponding histogram, containing 50 bins, is shown on the right. The top 25 correlating miRNAs are highlighted in orange, and the top five are annotated. (**B**) Overview of correlation coefficients of the top 25 miRNAs from panel A. miRNAs highlighted by Isakova et al. are shown in bold. (**C**) Empirical null distributions (grey) of correlation summary statistics based on 10 000 permutations of random assignments of non-target 7-mers to miRNAs. Only miRNAs expressed in $> 1\%$ of cells (*n* = 209) were included. Observed miRNA target motif statistics are highlighted in orange and the 99th percentile in black. (**D**) Differential miRNA expression analysis using a two-sided Wilcoxon rank-sum test between expression at days 0 and 12. Benjamini-Hochberg (BH) correction is performed to control the false discovery rate (FDR), and FDR $< 0.01$ are considered significant. The $\mathrm{log}_2$-transformed fold-change is the ratio between the mean miRNA expression at day 12 against day 0, with a small pseudocount of 0.1 added. (**E**) Median Pearson (left) and Spearman (right) correlation between expression and activity for significantly differentially expressed miRNAs (*n* = 68; panel D). Correlation is based on extracted cells from all time points (*n* = 913). Opacity indicates a differing number of miRNAs included (*n* = 43 for low opacity; miTEA-HiRes only recovers activities for a subset of miRNAs). (**F**) Boxplot with underlying data points depicting miR-298-5p expression (left) and activity (right) across four time points, measured in days. P-values are obtained from two-sided Wilcoxon rank-sum tests. TPM = transcripts per million. (**G**) Cell clustering based on UMAP coordinates. Cells are annotated by time point (left), miRNA expression (middle), and miRNA activity (right). The expression and activity are standardized for visualization purposes.

While scRNA-Seq datasets generally contain mRNA and other poly-adenylated RNA expression, a recent low-throughput protocol enables joint mRNA and miRNA quantification for single cells. To further evaluate the performance of bayesReact, we inferred the miRNA activity in a whole-transcriptome, low-throughput scRNA-Seq dataset ([28]; Supplementary Table S4). We considered the expression and activity of 225 miRNAs at four time points for mESCs differentiating from pluripotent stem cells (day 0) into embryoid bodies (day 12), which contain three germ layers of multipotent and lineage-committed stem cells. All miRNAs, except miR-298-5p, have a mean expression $< 2$$\log _2$ TPM, and only ten cases are expressed in more than 10% of the cells ($> 91$ of 913 cells; Supplementary Fig. S12A). The high zero count in the expression data entails both limited miRNA detection and resolution of the 3’ UTR sequence ranks, potentially explaining the reduced correlation scores compared to the bulk TCGA data (Fig. 3B–D, F, and G, Fig. 6A and B). Nevertheless, the correlation is significantly higher than for randomly sampled miRNA target motifs (Fig. 6C).

We compared the miRNA inference methods for significantly differentially expressed miRNAs between cells extracted on days 0 and 12. Only miRNAs expressed in $> 1\%$ of cells ($> 9$ of 913; *n* = 209) were evaluated, and we performed two-sided Wilcoxon rank-sum tests, followed by Benjamini-Hochberg correction for multiple testing (68 miRNAs had FDR $< 0.01$; Fig. 6D). Comparing bayesReact with previous methods, we find that bayesReact produces a higher median Pearson and Spearman correlation between miRNA activity and expression (Fig. 6E). All methods recover an increase in the miR-298-5p activity over time, which correlates with the observed expression ($\rho _p = 0.36$ for bayesReact, and $\rho _p \le 0.34$ using other methods; Fig. 6A, B, and F, Supplementary Fig. S12B-C). To our knowledge, this represents the first attempt at applying Sylamer and cWords to scRNA-Seq data. We find that both methods capture some of the same miRNA patterns as bayesReact, miReact, and miTEA-HiRes, e.g., all methods have miR-298-5p and miR-92-2-5p in their top correlating results (Fig. 6F, Supplementary Fig. S12C and D).

However, only bayesReact, and to a lesser extent cWords, meaningfully recovers the decrease in miR-92-2-5p activity during embryonic stem cell differentiation ($\rho _p = 0.22$ for bayesReact, and $\rho _p \le 0.22$ with other methods; Supplementary Fig. S12D). In contrast to the other methods, bayesReact infers a positive median activity when miR-92-2-5p is expressed (day 0) and a median close to zero when it is not (days 4–12; Supplementary Fig. S12D).

Focusing on the bayesReact output (Fig. 6A, B, F, and G), several of the top correlating miRNAs are also reported by Isakova et al. (Fig. 6B). This includes miR-298-5p, found to have increasing expression and activity during cell differentiation (Fig. 6F, Supplementary Fig. S12B), and miR-92-2-5p, having decreased expression and activity over time (Fig. 6G, Supplementary Fig. S12B and D). Additionally, miR-370-5p and miR-367-3p showed agreement between expression and activity (Supplementary Fig. S12E). In all cases, we observe a dropout in miRNA expression in a subset of cells, while the expression remains moderate to high in the remaining cells at the same time point. In contrast, the activity score does not exhibit a similar zero inflation (Fig. 6F, Supplementary Fig. S12D and E). A zero count can either be biological (no transcript present) or non-biological (failure to measure the transcript) [84, 86]. Since the inferred activity is based on an aggregated motif signal across all mRNA transcripts, we suggest it may be more robust to non-biological dropout. This is consistent with bayesReact continuing to recover the miR-122-5p liver-specific activity from increasingly small library sizes (Fig. 5C, Supplementary Fig. S11).

Investigating the top correlating miRNAs further (Fig. 6B), we find that the miR-297-669 cluster (including miR-297, miR-466, and miR-669) comprises many of the top correlating miRNAs (10 of 25, where only miR-669h-3p and miR-669k-3p share a seed site). The miRNA cluster is derived from an intronic locus in the Sfmbt2 gene [87], which has previously been shown to be expressed in mouse embryonic stem cells [88, 89]. Similarly, we observe miR-669c-3p expression in several of the mESCs from day 0 and positive activity for most cells at this time point (Supplementary Fig. S12E). The miRNAs from this cluster have been implicated in developmental, apoptotic, and toxic response processes [88–91]. Our findings further support the potential involvement of miRNAs from the miR-297-669 cluster in murine developmental processes.

#### microRNA activity inference for human and mouse PSCSR-seq data

**Figure 7.**
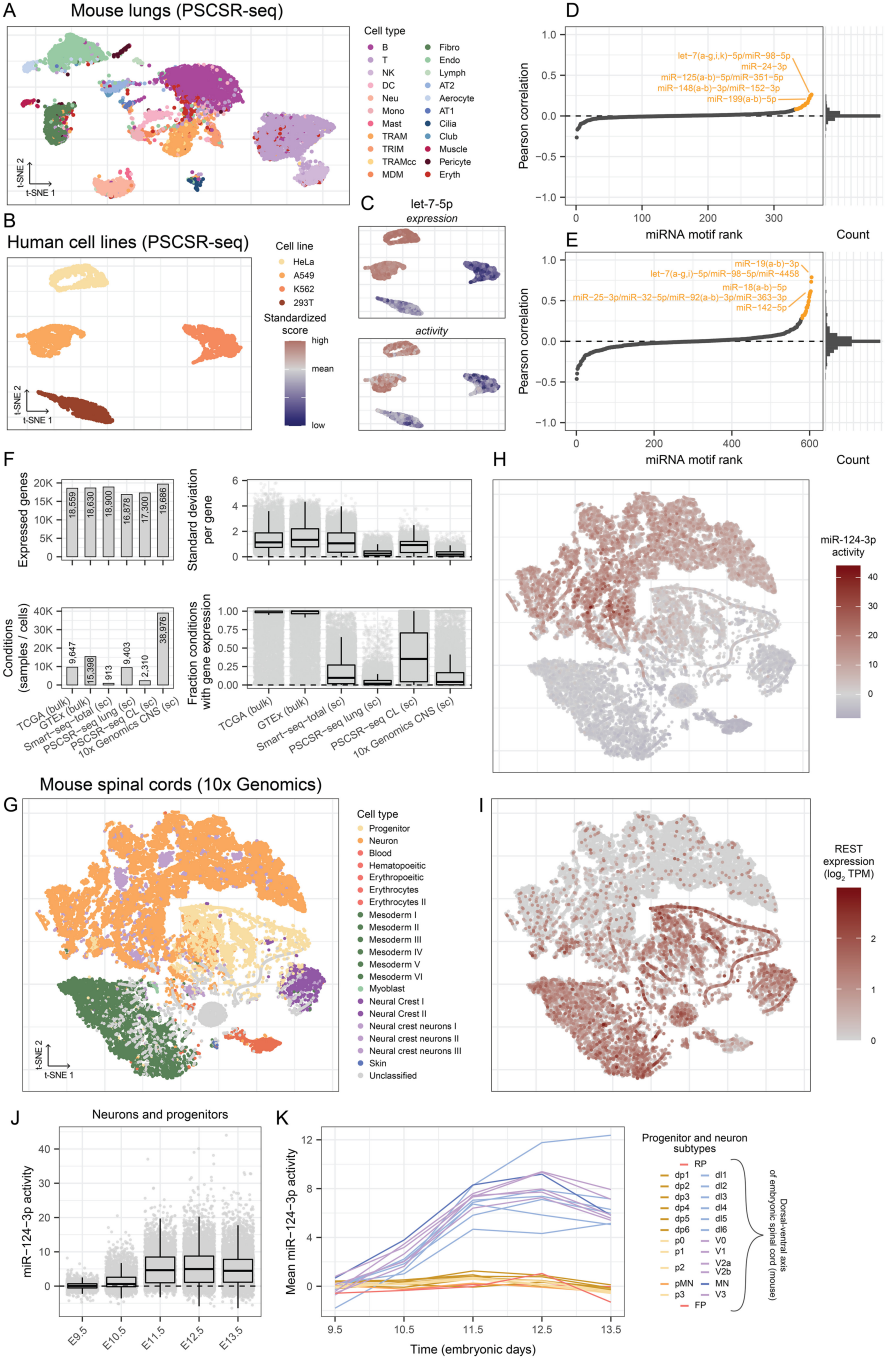
Single-cell microRNA activity inference. (**A**) Mouse lung cell clustering based on t-SNE coordinates (*n* = 9403). B = B cell; T = T cell; NK = Natural killer cell; DC = dendritic cell; Neu = Neutrophil; Mono = Monocyte; Mast = Mast cell; TRAM = Tissue-resident alveolar macrophage; TRIM = Tissue-resident interstitial macrophage; TRAMcc = TRAM in G2M cell-cycle phase; MDM = Monocyte-derived macrophage; Fibro = Fibroblast; Endo = general capillary endothelial cell; Lymph = Lymphatic endothelial cell; AT2 = Alveolar type II cell; Aerocyte = Alveolar endothelial cell subtype; AT1 = Alveolar type I cell; Cilia = Ciliated epithelial cell; Club = Nonciliated bronchiolar exocrine cell (club cell); Eryth = Erythroid cell. (**B**) t-SNE clustering of human cells (*n* = 2310) annotated by cell line. (**C**) Clustered cells from panel B annotated by let-7(a-g,i)-5p/miR-98-5p/miR-4458-5p expression (top) and inferred activity (bottom). (**D**) Ordered miRNA expression and activity correlations across all mouse lung cells (left) and corresponding 50 bin histogram (right). miRNA expression was collapsed by shared target site and then correlated to the corresponding motif activity. Top 25 miRNAs with the highest correlation are highlighted (orange). (**E**) Pearson correlation for all miRNAs expressed in the human cell lines. Plots match panel D. (**F**) Overview of gene expression variability across independent datasets. All underlying mRNA expressions are $\mathrm{log}_2$ pseudo-TPM values, which are used as input for bayesReact. Depicted for each dataset is the number of expressed protein-coding genes (top-left); the number of conditions (bottom-left); the standard deviation for the expression of each gene (top-right); and the fraction of conditions with the given gene expressed (bottom-right). sc = single-cell; CL = cell line; CNS = central nervous system. (**G**) Cell mapping based on t-SNE coordinates for cells in the developing mouse spinal cord single-cell atlas (*n* = 38 976). Cells are annotated by type, i.e., germ layer (stem cells), neural crest (derived from the ectoderm), or differentiated lineage-committed cells (hematopoietic lineage, neurons, etc.). Neuronal progenitors are termed progenitor (yellow). (**H**) miR-124-3p activity across clustered cells from embryonic mouse spinal cords, corresponding to panel A. (**I**) REST expression across differentiating mouse spinal cord cells. (**J**) miR-124-3p activity across embryonic days (E) for neuron and progenitor cells (*n* = 21 465). (**K**) Mean miR-124-3p activity for each neuron and progenitor subtype at each time point (embryonic day). The neuronal progenitors (left; legend) and neurons (right; legend) are organized based on the dorsal-ventral patterning around E9.5-13-5. Neurons are derived from eleven progenitor domains and are color-divided into dorsal interneurons (dI1-6), ventral interneurons (V0-3), and motor neurons (MN). The roof plate (RP) and floor plate (FP) cells help maintain the dorsal-ventral axis by producing signal gradients important for domain organization.

Paired miRNA and mRNA expression profiles were obtained from thousands of cells in mouse lung tissue (*n* = 9403) and human cell lines (*n* = 2310) [61], which were compared with inferred miRNA activities (Fig. 7A-E, Supplementary Figs S13–S15). The mouse lung data primarily comprise immune, endothelial, epithelial, and fibroblast cells (Fig. 7A), while the human cell lines included HeLa, A549, K562, and 293T (Fig. 7B). In both instances, the top correlating miRNAs (*n* = 25) in terms of expression and activity included several prominent and cell-type-specific cases, e.g., the let-7 family (Fig. 7C, Supplementary Fig. S13), several miRNAs from the miR-17-92 cluster implicated in lung development (Supplementary Fig. S14B; [92, 93]), as well as miR-19(a-b)-3p and miR-92(a-b)-3p, mainly expressed in K562 (Supplementary Fig. S15B and C). In addition, several miRNAs emphasized by Li et al. [61] were also among the top correlating results for both the mouse (miR-22-3p, miR-142a-5p; Supplementary Fig. S14A-C) and human (miR-92a, miR-98-5p; Supplementary Fig. S15A–C) cells.

Compared to previous methods, bayesReact performs favorably based on both median and 90th percentile correlation across all expressed mouse (*n* = 357) and human (*n* = 606) miRNAs (Supplementary Fig. S14D, Supplementary Fig. S15D and E). However, for the mouse lung cells, the correlation distribution is symmetric around zero and lacks an enriched positive tail (Fig. 7D). A cross-dataset examination of the underlying mRNA expression profiles used for activity inference reveals low expression variability in tissue-derived single-cell datasets, including the mouse lung cells. Furthermore, compared to other datasets, most genes are expressed in a small fraction of cells (Fig. 7F). Consequently, it is relevant to consider the number of expressed genes, expression variability, and fraction conditions with gene expression, as these factors influence gene (sequence) ranking and quality of the activity inference.

#### miR-124-3p activity across the developing spinal cord in mice

As previously described, miR-124-3p is primarily expressed in neurons in both humans and mice, and is important for neuronal cell fate decisions [73, 74]. In agreement, we observe that the expression and activity of miR-124-3p are highest in samples from healthy brain tissue (GTEx) and low-grade glioma (TCGA). However, the cell-level heterogeneity remains unobserved. We acquired a single-cell atlas of the developing spinal cord in mice (*n* = 38 976), with cells extracted from spinal cords dissected at embryonic day E9.5, E10.5, E11.5, E12.5, and E13.5 [62]. Using bayesReact, we inferred the miRNA activity across all cells and found the miR-124-3p activity to be the largest in differentiated neurons (Fig. 7G and H) as well as having a negative correlation with the observed REST expression ($\rho _p = -0.23$, Fig. 7I).

Similar results are observed for miR-9-5p and let-7-5p, which are also involved in cell fate decisions [94] (Supplementary Fig. S16).

Embryonic development of the CNS, including the spinal cord, is a tightly regulated spatio-temporal process, where signal gradients give rise to unique cell domains and migration patterns from which different progenitors and neuron subtypes originate [62, 95]. Considering the subset of neuronal cells (*n* = 21 465), an increase in the miR-124-3p activity is observed over time, which may be a product of the increasing number of mature neurons (Fig. 7J). Interestingly, the mean miR-124-3p activity increases over time for all differentiated neuron subclasses, but remains constantly close to zero for all corresponding progenitors (mean of 5.7 across all neurons and 0.3 for all progenitors; Fig. 7K). Our finding of increased miR-124-3p activity during neuronal differentiation is consistent with its known function. The results highlight the value of activity inference for exploring miRNA patterns at the single-cell level and across atlases, where direct measurements of miRNA expression are currently absent.

#### The impact of the microRNA reference database

A low correlation between miRNA expression and inferred activity can reflect either the failure of the inference method to capture the underlying activity or be due to other factors, such as low miRNA abundance (Supplementary Fig. S7A), modified or inactive miRNAs, a non-linear relationship, or incorrect miRNA annotations. Consequently, activity inference methods remain challenging to benchmark.

**Figure 8.**
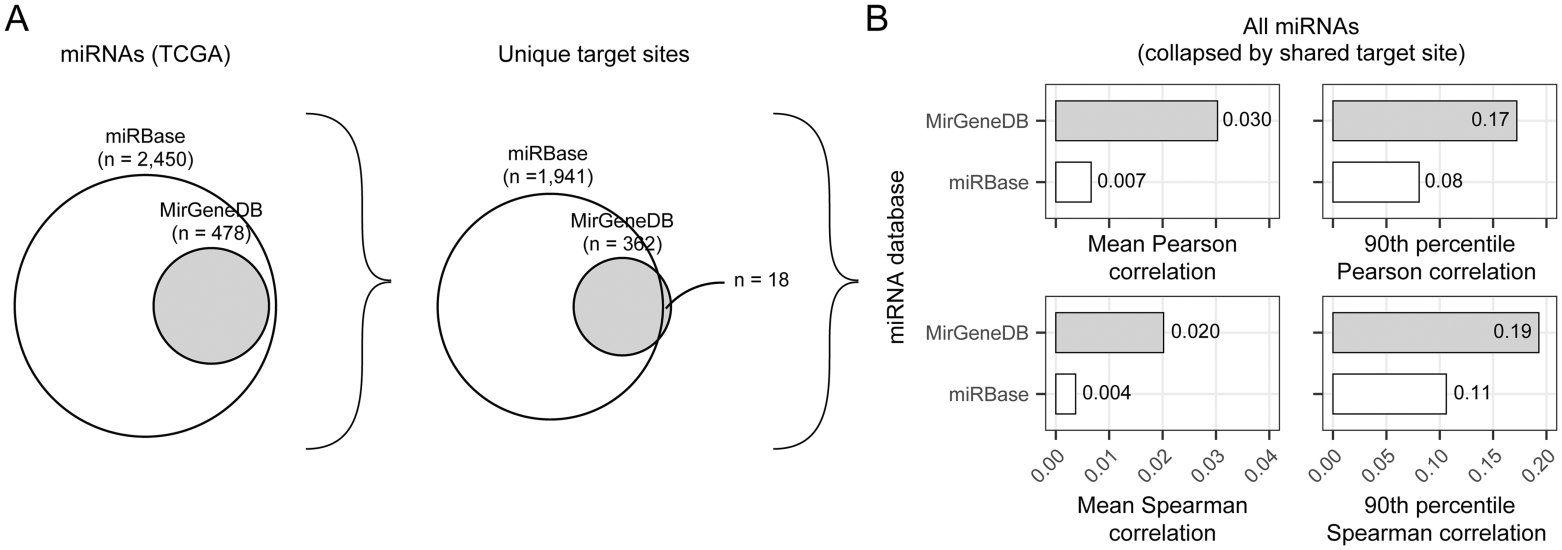
Comparison of miRBase and MirGeneDB microRNA gene annotations. (**A**) Subset of miRBase miRNAs expressed in the TCGA data also present in MirGeneDB (left), and overlap in unique target sites (right). (**B**) Mean correlation (left) and 90th percentile (right) for the miRNA Pearson and Spearman correlations across all pan-cancer TCGA samples (*n* = 9640). The collapsed miRNA expression is correlated with the activity of shared target sites for each reference database.

We have used miRBase to annotate miRNAs and their seed sites. However, it includes many weakly supported entries likely representing false positives (FPs) [96–100]. To assess the impact of these annotations on benchmarking, we compared miRBase with the manually curated MirGeneDB [64], aiming at minimizing FPs at the potential expense of excluding some genuine miRNAs. Encouragingly, miRNAs annotated in both miRBase and MirGeneDB (Fig. 8A) have a greater mean and 90th percentile correlation between expression and activity for datasets with separate 5p and 3p arm expression (Fig. 8B, Supplementary Fig. S17).

Focusing on the pan-cancer TCGA data, which has the greatest discrepancy between miRBase and MirGeneDB entries, only the MirGeneDB miRNAs (*n* = 362), and not the miRBase entries (*n* = 1941), consistently achieve significant correlation statistics greater than the 99th percentile from the null distribution of random target motif assignments (Supplementary Fig. S18A). Furthermore, miRBase miRNAs absent from MirGeneDB tend to have low correlation scores (Supplementary Fig. S18B). miRNAs with the highest mean expression (*n* = 50) tend to represent high-confidence annotations, and little difference is observed between the two databases in terms of annotations and correlations (Supplementary Fig. S18C).

## Discussion

We present a new tool for inferring regulatory motif activity, which can recover miRNA activities at both bulk and single-cell levels. We focused on the activity inference of miRNAs, as they are highly studied and well-characterized, making them ideal for model validation. However, many other regulators promote cell homeostasis through motif interaction [6, 8, 9, 101]. In a recent study, we used miReact for *de novo* motif discovery, which led to the finding of a circHIPK3/IGF2BP2 regulatory axis [102], depicting the use of activity inference for RBPs. Similarly, bayesReact presents a generic hypothesis-generating tool that can also be used for large data screens. It may help detect active motif-based regulatory mechanisms and perform *de novo* motif discovery, subsequently identifying candidates for further computational and experimental validation. We focus here on expression-coupled motif modeling using RNA-Seq data for sequence ranking.

However, other continuous experimental settings could also be used. Advancements in high-throughput proteomics [103, 104] and image-based spatial transcriptomics [105] represent anticipated novel avenues.

bayesReact implements a generative process to model motifs across ranked sequence lists. It assumes equal functional consequences, e.g., target depletion, for each motif occurrence. However, miRNAs are expected to act on a single to hundreds of mRNA targets [10, 106], yet their 7-mer target site often occurs in $> 1000$ 3’ UTRs. Many instances are subsequently false positives that occur by random chance or are inactive in a given condition. The motif prevalence is partially explained by the use of the longest 3’ UTR isoform from each gene, whose 3’ UTR length is normally unique for each tissue and cell type, allowing for heterogeneous regulation of the mRNA transcript [107, 108]. False positives are also attributed to randomly occurring target sites in genes not expressed in the same tissue as the given miRNA and subsequently have no regulatory effect. The simplified miRNA-target binding model using nt positions 2-8 of the mature miRNA as its seed site may also contribute to random motif occurrence, not accounting for extended sequence context dependencies [15]. In addition, the binding model does not account for target accessibility, e.g., through differing subcellular miRNA and mRNA localization, and secondary RNA structure of the 3’ UTR [15, 106]. Subsequently, the regulatory effect of miRNAs may differ across target sites and conditions, ranging from no effect to efficient target depletion [15, 106, 108, 109]. We still have a limited understanding of the tissue- and cell-level miRNA target efficiency. Moving forward, extending bayesReact to incorporate additional biological features could help better ascertain regulatory heterogeneity by jointly modeling miRNA activity and target efficiency.

bayesReact currently uses a pseudo-normal condition for FC-based 3’ UTR ranking, defined as the median expression of genes across all conditions, e.g., the TCGA samples. Subsequently, the 3’ UTR rank and location along the combined sequence interval are expected to be driven by transcriptional differences between tissues and cancer types. We find that bayesReact can efficiently recover the activity of miRNAs in cancer types where the corresponding miRNA is also expressed. However, when investigating regulatory perturbations during tumorigenesis, we must compare healthy and disease conditions [44], which can be challenging due to the limited access to healthy control samples. In such instances, computational methods may pave the way for 3’ UTR ranking relative to an inferred healthy control setting [110]. This may aid in investigating activity perturbation in cancer, supplementing the examination of the prognostic and therapeutic potential of miRNAs and other regulators [17, 18].

We report a strong negative association between the miR-124-3p activity and REST expression in low-grade gliomas, agreeing with the known negative feedback loop in which both miR-124-3p and the REST protein complex participate in the regulation of neuronal cell fate [75, 78]. Down-regulation of miR-124-3p and up-regulation of REST have been implicated in the development and prognosis of gliomas, including the gain of stem-like features, e.g., self-renewal, and tumor invasiveness [23, 111–117]. However, limited knowledge exists regarding the joint deregulation of the regulatory miR-124-3p/REST axis in cancer [113]. We propose that miR-124-3p down-regulation in gliomas alleviates the repression of REST, which would further implicate its tumor-suppressor capabilities and therapeutic potential. In the future, inference of healthy control samples may further help us evaluate the perturbation of miR-124-3p activity and REST expression in TCGA low-grade glioma samples.

Our results highlight that miRNA activity inference may contribute information even when the miRNA expression is observed, e.g., by elucidating differences in the degree to which a miRNA acts on its targets or by detecting potential dropout events in whole-transcriptome scRNA-Seq data. For example, Isakova et al. did not originally report on the miR-297-669 cluster, which may be due to its low expression in their data. However, the combined expression and activity of miR-669c-3p from the miRNA cluster indicate its presence in the earliest stage of murine stem-cell differentiation, concordant with the implicated role of the miR-297-669 cluster in developmental processes, particularly placental development [88, 89]. A key prospect of mRNA-based regulatory inference is the use on RNA-Seq data lacking short transcripts, which includes the majority of scRNA-Seq data to date [26]. bayesReact is an unsupervised method that allows for direct miRNA activity inference from high-throughput scRNA-Seq experiments, where miRNA expression is unavailable. For example, we demonstrate that a neuron-specific miR-124-3p activity can be recovered from an embryonic mouse spinal cord single-cell atlas, refining the CNS-specific miRNA expression observed in bulk RNA-Seq data. bayesReact can potentially be further optimized for sparse count data through a hierarchical framework, enabling the activity to be tissue or cell-type-specific.

In conclusion, we introduce the versatile hypothesis-generating tool bayesReact, which improves the ability to infer condition-specific regulatory motif activity at the single-cell level. The unsupervised method enables comprehensive data screens for regulatory activity detection and provides uncertainty evaluation for activity estimates. It predicts miRNA activities that positively correlate with the corresponding measured expressions in bulk and single-cell RNA-Seq data. We highlight the ability of activity inference to contribute additional information to miRNA expression by shedding light on the association between miRNA activity and known target expression, and potentially being more robust to dropout events in Smart-seq-total data. Furthermore, bayesReact outperforms its predecessors (Sylamer, cWords, miReact, and miTEA-HiRes) on sparse count data from multiple independent studies, and recovers significant temporal miRNA activity patterns in agreement with observed miRNA expression and current literature.

## Supplementary Material

gkag072_Supplemental_Files

## Data Availability

All data used during this study are publicly available. Matched mRNA and miRNA expression data from primary tumors were obtained from the Cancer Genome Atlas through the Recount3 project and GDC data portal [55–57]. Paired expression data from healthy tissue samples were retrieved through the Genotype-Tissue Expression project [58, 59] using the GTEx portal (https://www.gtexportal.org). Whole-transcriptome, single-cell Smart-seq-total data were obtained from Isakova et al. [28], through the Gene Expression Omnibus (GEO) using the accession number GSE151334. From Li et al. [61], using GEO and accession number GSE226714, we also acquired PSCSR-seq data from mouse lung biopsies and human cell lines. The processed murine single-cell atlas of embryonic spinal cord development, generated by Delile et al. [62], was retrieved from ArrayExpress (accession number E-MTAB-7320). Inferred miRNA activity tables from the TCGA and Smart-seq-total data can be found in Supplementary Figs S2 and S4, respectively. The bayesReact code is publicly available through Zenodo (https://doi.org/10.5281/zenodo.18236089) and the latest release at GitHub (https://github.com/JakobSkouPedersenLab/bayesReact).
