# Supplementary material for: bayesReact: expression-coupled regulatory motif analysis detects microRNA activity across cancers, tissues, and at the single-cell level
Source: Nucleic Acids Res. 2026 Feb 9;54(4):gkag072. doi: 10.1093/nar/gkag072 (PMC12884093; doi:10.1093/nar/gkag072)
Supplement: gkag072_Supplemental_Files [file gkag072_supplemental_files.zip › Supplementary_material.pdf]

Supplementary material

Asta M. Rasmussen,[1,2,*] **Alexandre Bouchard-Côté**[3,†] **and Jakob S. Pedersen**[1,2,4,*,†]

[1]Department of Clinical Medicine, Aarhus University, Palle Juul-Jensens Boulevard 11, 8200, Aarhus N, Denmark, [2]Department of Molecular Medicine, Aarhus University Hospital, Palle Juul-Jensens Boulevard 11, 8200, Aarhus N, Denmark, [3]Department of Statistics, University of British Columbia, 2207 Main Mall, V6T 1Z4, British Columbia, Canada and [4]Bioinformatics Research Center, Aarhus University, Universitetsbyen 81, 8000, Aarhus C, Denmark

*To whom correspondence should be addressed. astamr@clin.au.dk and jakob.skou@clin.au.dk.
†The authors wish it to be known that, in their opinion, the last two authors should be regarded as Joint Last Authors.

Supplementary Methods

The supplementary methods constitute a comprehensive description of the model framework and connection to null model expectations. It supplements the Materials and methods section, and the sub-section naming therefore matches the main text in the paper.

Modeling motif activity

Data representation and null expectation

Let $\{S_{1,c}, S_{2,c}, \ldots, S_{s,c}, \ldots, S_{N,c}\}$ be a set of FC-score (Fold Change) ranked sequences, where the index $s \in \{1, \ldots, N\}$ defines the rank, $c \in \{1, \ldots, C\}$ the condition, and $m \in \{1, \ldots, M\}$ the set of M independent motifs (Figure 1). The ranked sequences are arranged consecutively to represent non-overlapping intervals in a given condition c, and the number of motif occurrences in each sequence, for a motif m, are considered independently Poisson-distributed random variables $n_{s,c,m} \sim \text{Pois}(\lambda_{s,c,m})$. From the sequence-specific probability, it is possible to define the Poisson rate parameter $\lambda_{s,c,m}$ as follows: $SSP_{s,c,m} = 1 - P(n_{s,c,m} = 0) = 1 - \frac{\lambda^0_{s,c,m}}{0!} \cdot e^{-\lambda_{s,c,m}} = 1 - e^{-\lambda_{s,c,m}} \Rightarrow \lambda_{s,c,m} = -\ln(1 - SSP_{s,c,m})$. We can thus represent each $S_{s,c}$ as a sub-interval with width corresponding to the expected number of motif occurrences given by $\lambda_{s,c,m}$. This normalizes for the probability of observing m given the sequence length and nucleotide composition of $S_{s,c}$. Consequently, the total number of motif counts can also be described through a Poisson process, $n_m = \sum_{s=1}^{N} n_{s,c,m} \sim \text{Pois}(\lambda_m)$, where $\lambda_m = \sum_{s=1}^{N} \lambda_{s,c,m}$ is the length of the combined sequence interval $[0, \lambda_m]$, which represents the total expected number of motif occurrences. n_m and λ_m are motif-specific and constant across all conditions, and the c index is therefore dropped for notational convenience. Conditioning on n_m, we can describe the motif count observations jointly as $\mathbf{n_{c,m}} = (n_{1,c,m}, \ldots, n_{s,c,m}, \ldots, n_{N,c,m}) \mid n_m \sim \text{Multinom}(n_m, \mathbf{l_{c,m}} = (l_{1,c,m}, \ldots, l_{s,c,m}, \ldots, l_{N,c,m}))$, where $l_{s,c,m} = \frac{\lambda_{s,c,m}}{\sum_{j=1}^{N} \lambda_{j,c,m}}$ and $\mathbf{l_{c,m}}$ is a vector of probabilities. Here $\tilde{l}_{s,c,m}$ is the normalized and re-scaled representation of $S_{s,c}$ in relation to motif m, with length $|\tilde{l}_{s,c,m}| = l_{s,c,m}$ such that $\sum_{s=1}^{N} l_{s,c,m} = 1$ (Figure 1D). Specifically, $\tilde{l}_{s,c,m} = [L_{s-1,c,m}, L_{s,c,m}]$, where $L_{s,c,m} = \sum_{j=1}^{s} l_{j,c,m}$ is the cumulative sum of sequence lengths. Motif occurrence $k_{s,c,m}$ can be described as a stochastic process along the re-scaled sequence interval. The exact motif position on $[0, 1]$ is unobserved and considered an auxiliary latent variable. Given the data processing and representation, the null model of no motif activity is a uniform distribution of motifs across the ranked and normalized sequences, which entails no association between relative sequence abundance (rank) and motif occurrence:

$$
\begin{aligned}
k_{s,c,m} &\sim \text{Unif}(0, 1) \\
\mathbf{n_{c,m}} \mid n_m &\sim \text{Multinom}(n_m, \mathbf{l_{c,m}}).
\end{aligned} \tag{1}
$$

Consequently, a non-functional motif is expected to occur with equal probability across $[0, 1]$ when we account for sequence length and nucleotide composition bias. The null model for the number of motif occurrences $n_{s,c,m}$ in a sequence $S_{s,c,m}$ depends only on the length of its sub-interval $l_{s,c,m}$.

Modeling motif occurrence and number of motifs across ranked sequences

Deviations from the null model described above can, for example, arise when a microRNA acts on a set of target transcripts. The depleted target sequences will be systematically skewed toward the end of the ranked sequence list (Figure 1E). This signal can be captured by letting the motif occurrence be distributed according to a flexible family of beta distributions, all with support on $[0, 1]$. The null model then becomes a special case, $\text{Unif}(0, 1) \overset{d}{=} \text{Beta}(1, 1)$, and the probability density function (PDF; $f(k_{s,c,m}; a_{c,m})$) is given by:

$$
\begin{aligned}
f(k_{s,c,m}; a_{c,m}) &= \frac{{k_{s,c,m}}^{\alpha(a_{c,m})-1}(1 - k_{s,c,m})^{\beta(a_{c,m})-1}}{\text{B}(\alpha(a_{c,m}), \beta(a_{c,m}))} \\
\alpha(a_{c,m}) &= \mathbf{1}[a_{c,m} < 0] + \mathbf{1}[a_{c,m} \geq 0](1 + a_{c,m}) \\
\beta(a_{c,m}) &= \mathbf{1}[a_{c,m} < 0](1 - a_{c,m}) + \mathbf{1}[a_{c,m} \geq 0].
\end{aligned} \tag{2}
$$

The beta shape parameters are here transformations of an underlying activity parameter $a_{c,m} \in \mathbb{R}$. The activity parameter has a clean interpretation concerning motif distribution; $a_{c,m} < 0$ entails motif clustering at the beginning of the combined sequence interval constituting sequences with high relative gene expression (FC-scores); $a_{c,m} = 0$ corresponds to the null model; and $a_{c,m} > 0$ implies motif over-representation at the end of $[0, 1]$ for sequences with low relative abundance (Figure 1E).

Under the beta distribution, we can describe the probability of motif occurrence on $S_{s,c,m}$ as $p_{s,c,m} = \int_{\tilde{l}_{s,c,m}} f(k_{s,c,m}; a_{c,m}) dk_{s,c,m}$, where $\mathbf{p_{c,m}} = (p_{1,c,m}, \ldots, p_{s,c,m}, \ldots, p_{N,c,m})$ is a vector of motif probabilities. The number of motif occurrences on each sequence can subsequently be described by a conditional multinomial distribution:

$$
\mathbf{n_{c,m}} \mid n_m, a_{c,m} \sim \text{Multinom}(n_m, \mathbf{p_{c,m}}). \tag{3}
$$

While $p_{s,c,m}$ can be found by evaluating the integral $\int_{\tilde{l}_{s,c,m}} f(k_{s,c,m}; a_{c,m}) dk_{s,c,m}$, obtained through the cumulative distribution function (CDF; $F(.)$), this is computationally intensive for large data sizes. Instead, we approximate the beta distribution with a step-function, where $\int_{\tilde{l}_{s,c,m}} f(k_{s,c,m}; a_{c,m}) dk_{s,c,m} \approx l_{s,c,m} \cdot f(r_{s,c,m}; a_{c,m})$ and $r_{s,c,m}$ is the mid-point of the normalized sequence interval for $S_{s,c,m}$ (Figure 1D-E). Increasing the total number of sequences N, leads to a finer partitioning of $[0, 1]$, e.g., using all human 3' UTRs divides the combined sequence interval into ∼20K sub-intervals. For all $k_{s,c,m}$ on $\tilde{l}_{s,c,m}$, we have that $|k_{s,c,m} - r_{s,c,m}| \leq \frac{l_{s,c,m}}{2}$ and we approximate $f(k_{s,c,m}; a_{c,m}) \approx f(r_{s,c,m}; a_{c,m})$ as defined by a step-function.

The approximation may introduce a slight bias when the second derivative of the beta distribution is nonzero within a sub-interval (curvature of the beta density), leading to a slight over- or underestimation of the motif occurrence probability within a sequence. The derivative is assumed to be constant within sufficiently small intervals, and any bias may be further mitigated by using a higher-order Taylor expansion when approximating the beta density. However, we show in Supplementary Figure S5C that the results obtained using the exact and approximate densities are highly correlated. Subsequently, the joint probability of the motif counts across all sequences, conditional on the underlying activity parameter, can be approximated by the following parameterization of the multinomial probability mass function (PMF):

$$\begin{aligned} P(\mathbf{n_{c,m}} \mid a_{c,m}) &\propto \prod_{s=1}^{N} {p_{s,c,m}}^{n_{s,c,m}} \\ &\approx \prod_{s=1}^{N} \left(l_{s,c,m} \cdot f(r_{s,c,m}; a_{c,m})\right)^{n_{s,c,m}} . \end{aligned} \tag{4}$$

An additional benefit of approximating $\mathbf{p_{c,m}}$ is the ability to pre-compute part of the log-likelihood (orange underlining), allowing for further computational speed-up:

$$\begin{aligned} \log P(\mathbf{n_{c,m}} \mid a_{c,m}) &\propto \sum_{s=1}^{N} n_{s,c,m} \left(\log(l_{s,c,m}) + \log(f(r_{s,c,m}; a_{c,m}))\right) \\ &= \sum_{s=1}^{N} n_{s,c,m} \cdot \log(l_{s,c,m}) \\ &\quad + \sum_{s=1}^{N} n_{s,c,m} \cdot \log\left(\frac{{r_{s,c,m}}^{\alpha(a_{c,m})-1}(1-r_{s,c,m})^{\beta(a_{c,m})-1}}{\mathrm{B}(\alpha(a_{c,m}), \beta(a_{c,m}))}\right) \\ &= \underline{\sum_{s=1}^{N} n_{s,c,m} \cdot \log(l_{s,c,m})} \\ &\quad + (\alpha(a_{c,m}) - 1)\underline{\sum_{s=1}^{N} n_{s,c,m} \cdot \log(r_{s,c,m})} \\ &\quad + (\beta(a_{c,m}) - 1)\underline{\sum_{s=1}^{N} n_{s,c,m} \cdot \log(1 - r_{s,c,m})} \\ &\quad - \underline{n_m} \cdot \log\left(\mathrm{B}(\alpha(a_{c,m}), \beta(a_{c,m}))\right) . \end{aligned} \tag{5}$$

Finally, we place an uninformative prior on the activity parameter centered at zero: $a_{c,m} \sim \mathrm{N}(\mu = 0, \sigma^2 = 100)$.

By establishing the log-likelihood and prior distribution of $a_{c,m}$, it is possible to explore the marginal posterior density of interest, after marginalizing the latent variable $k_{s,c,m}$:

$$\begin{aligned} P(a_{c,m} \mid \mathbf{n_{c,m}}) &= \int_{k_{1:N,c,m}} P(a_{c,m}, k_{1:N,c,m} \mid \mathbf{n_{c,m}}) dk_{1:N,c,m} \\ &\propto \int_{k_{1:N,c,m}} P(a_{c,m}, k_{1:N,c,m}, \mathbf{n_{c,m}}) dk_{1:N,c,m} \\ &= P(a_{c,m}, \mathbf{n_{c,m}}) = P(\mathbf{n_{c,m}} \mid a_{c,m}) P(a_{c,m}) \Rightarrow \\ \log P(a_{c,m} \mid \mathbf{n_{c,m}}) &\propto \log P(\mathbf{n_{c,m}} \mid a_{c,m}) + \log P(a_{c,m}). \end{aligned} \tag{6}$$

MCMC sampling is used to sample from the stationary target distribution of interest (proportional to the posterior distribution; referred to interchangeably as posterior).

Flexible two-parameter beta model

A more flexible model was also implemented and evaluated, where the two beta parameters $\alpha_{c,m}$ and $\beta_{c,m}$ are freely variable instead of transformations of $a_{c,m}$. The two-parameter beta model and corresponding activity are defined as follows:

$$\begin{aligned}
\alpha_{c,m} &\sim \mathrm{Exp}(0.1) \\
\beta_{c,m} &\sim \mathrm{Exp}(0.1) \\
k_{s,c,m} \mid \alpha_{c,m}, \beta_{c,m} &\sim \mathrm{Beta}(\alpha_{c,m}, \beta_{c,m}) \\
\tau_{c,m} &= \mathbb{E}[k_{s,c,m} \mid \alpha_{c,m}, \beta_{c,m}] = \frac{\alpha_{c,m}}{\alpha_{c,m} + \beta_{c,m}}
\end{aligned}$$

$$activity_{c,m} = \begin{cases} \mathrm{sgn}(\bar{\tau}_{c,m} - 0.5) \cdot \log P(\tau_{c,m} \leq 0.5 \mid \mathbf{n_{c,m}}) + \log(2), & \bar{\tau}_{c,m} \geq 0.5 \\ \mathrm{sgn}(\bar{\tau}_{c,m} - 0.5) \cdot \log P(\tau_{c,m} \geq 0.5 \mid \mathbf{n_{c,m}}) + \log(2), & \bar{\tau}_{c,m} < 0.5, \end{cases} \tag{7}$$

where $\bar{\tau}_{c,m}$ is the mean value of the marginal posterior density $P(\tau_{c,m} \mid \mathbf{n_{c,m}})$. We refer to the two models as bayesReact and $\mathrm{bayesReact}_{\mathrm{2p}}$, respectively.

Supplementary Figures

This section contains the following supplementary figures:

- **Supplementary Figure 1.** Review of microRNA inference methods.
- **Supplementary Figure 2.** Overview of bayesReact input processing.
- **Supplementary Figure 3.** bayesReact content.
- **Supplementary Figure 4.** Model diagnostics and evaluation.
- **Supplementary Figure 5.** Evaluation of log-likelihood, posterior approximations, and computational resource comparisons.
- **Supplementary Figure 6.** Heatmaps depicting clustering of pan-cancer samples.
- **Supplementary Figure 7.** Overall pan-cancer performance of microRNA activity inference methods.
- **Supplementary Figure 8.** Cancer-type-specific miR-122-5p and miR-9-5p activity comparison between methods.
- **Supplementary Figure 9.** microRNA activity inference across healthy tissue samples.
- **Supplementary Figure 10.** Cancer-type-specific miR-122-5p and miR-124-3p activities.
- **Supplementary Figure 11.** miR-122-5p activity inference based on differing degrees of library count down-sampling.
- **Supplementary Figure 12.** Recovering microRNA activities at the single-cell level from mouse embryonic stem cells.
- **Supplementary Figure 13.** let-7 microRNA family expression and activity across PSCSR-seq datasets.
- **Supplementary Figure 14.** MicroRNA expression and activity across cells from mouse lung biopsies.
- **Supplementary Figure 15.** Human cell line microRNA expression and activity.
- **Supplementary Figure 16.** Inferred miR-9-5p and let-7-5p activities during spinal cord development in mice.
- **Supplementary Figure 17.** Comparison of miRBase and MirGeneDB microRNA annotations across multiple independent datasets.
- **Supplementary Figure 18.** miRBase and MirGeneDB comparison for pan-cancer TCGA data.

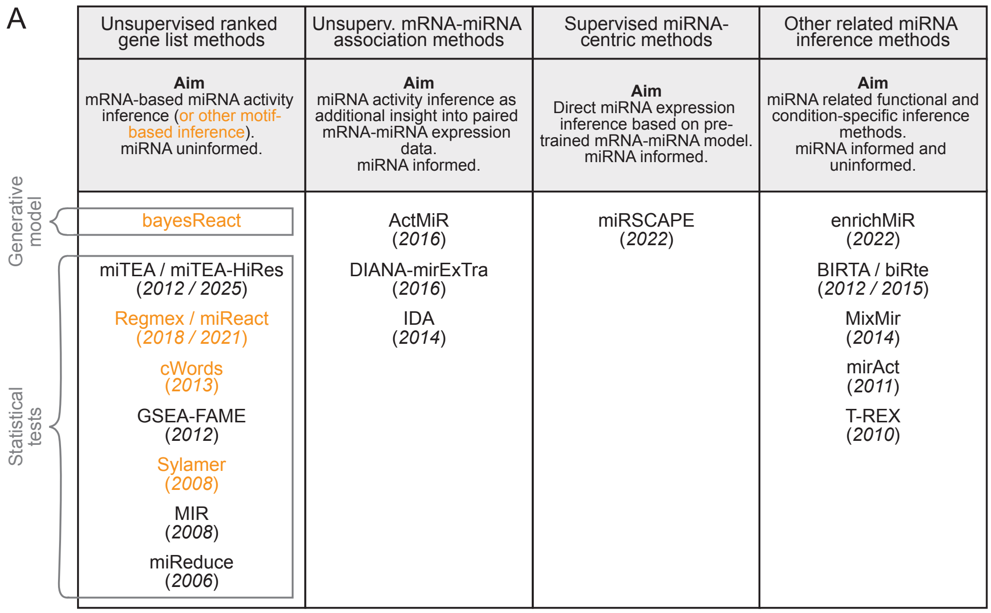

Supplementary Figure 1. Review of microRNA inference methods. (**A**) miRNA activity and expression inference methods ordered by release date and subdivided into columns by inference strategy and aim. miRNA informed and uninformed refer to whether a method directly uses miRNA expression profiles to make inferences, e.g., through model training or correlation scores. enrichMiR and mirAct are also tools for miRNA activity inference, prioritizing user-friendliness. However, they are only provided through web-based interfaces, which inhibit scalability (exploring multiple samples simultaneously) and generability (exploring a wide variety of settings, e.g., cross data types and species). The list of methods is expected to be inexhaustive, and the methods may be applicable to different data types. Some are designed for microarray data, while others are designed for the bulk or single-cell RNA-Seq settings.

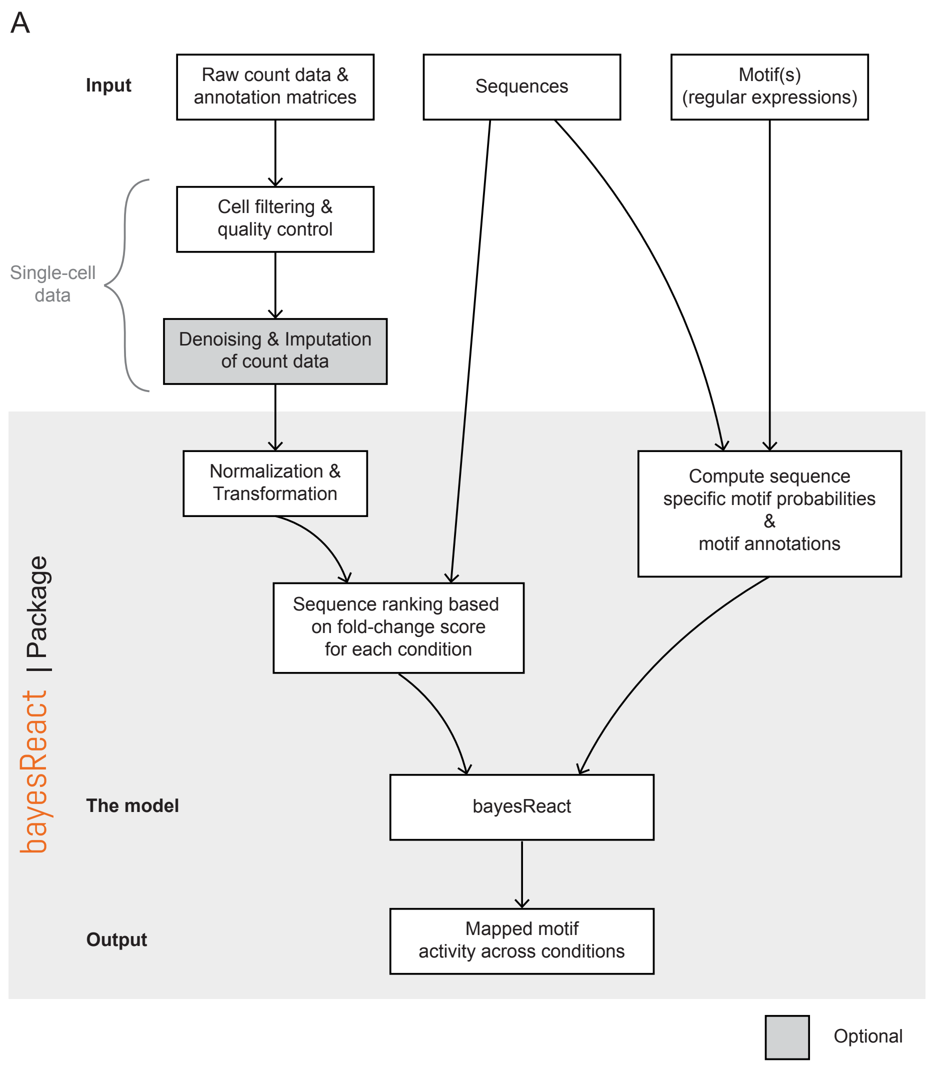

Supplementary Figure 2. Overview of bayesReact input processing. (**A**) bayesReact takes as input filtered expression data, a set of sequences, and regulatory motifs of interest. After manual preprocessing, the bayesReact package handles expression data normalization, sequence ranking, motif annotations, and modeling (highlighted with a grey background). Edges show the flow of data between each processing step.

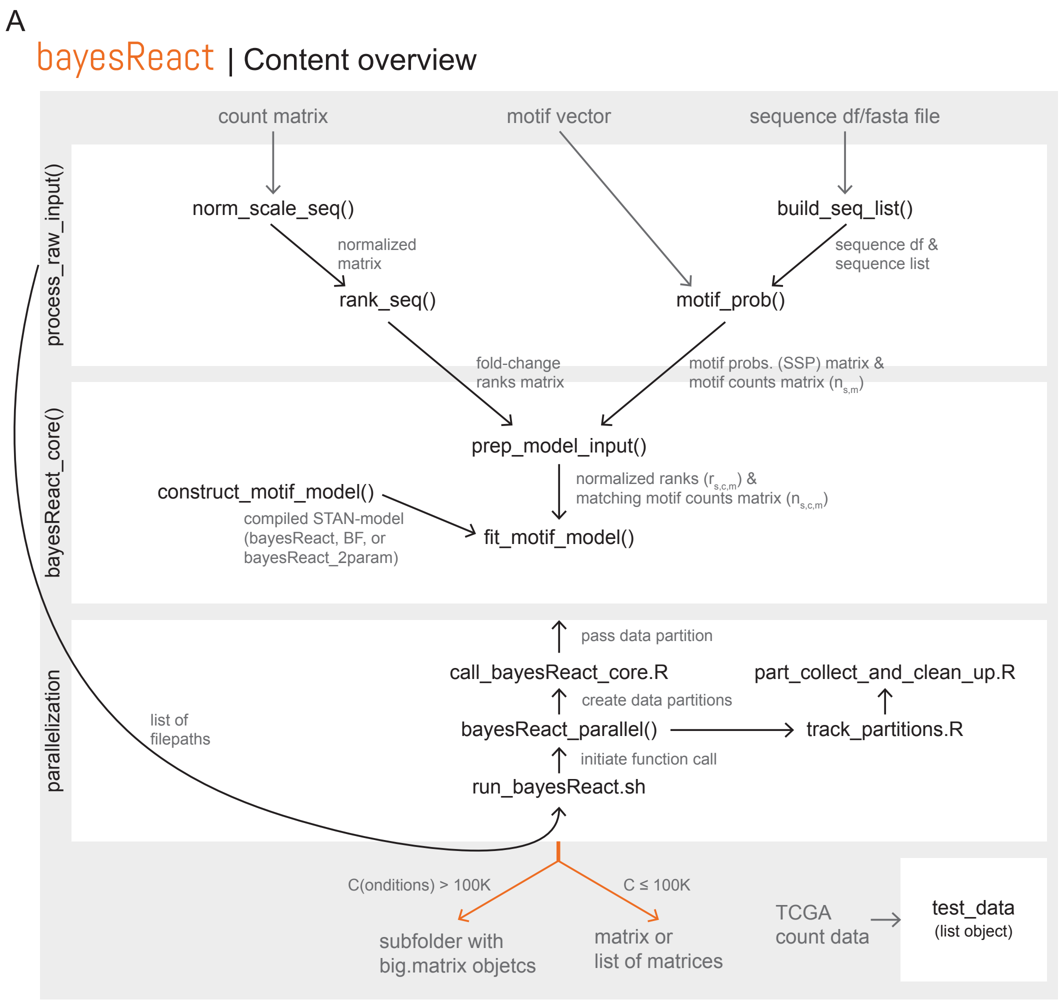

Supplementary Figure 3. bayesReact content. (**A**) Overview of functions and scripts provided in the R-package (black) and the data input (grey), with edges depicting the data flow between function calls. bayesReact contains three main components (white boxes): process_raw_input(), bayesReact_core(), and bayesReact_parallel(). 100K = 100,000.

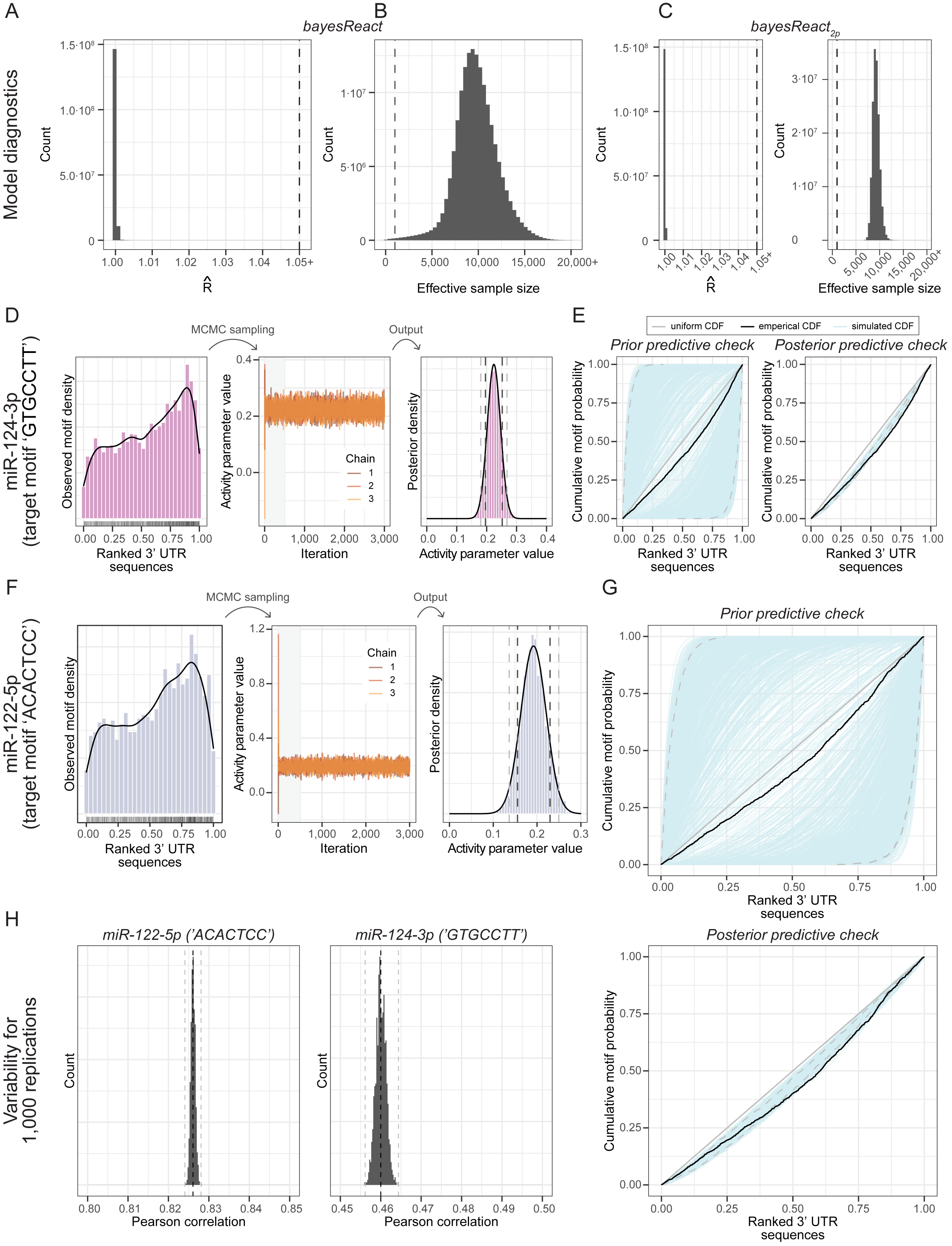
A
B
C
Model diagnostics
bayesReact
bayesReact2p
Count
R̂
Effective sample size
D
E
miR-124-3p (target motif 'GTGCCTT')
MCMC sampling
Output
Observed motif density
Activity parameter value
Posterior density
Chain
Ranked 3' UTR sequences
Iteration
uniform CDF
emperical CDF
simulated CDF
Prior predictive check
Posterior predictive check
Cumulative motif probability
F
G
miR-122-5p (target motif 'ACACTCC')
H
Variability for 1,000 replications
miR-122-5p ('ACACTCC')
miR-124-3p ('GTGCCTT')
Pearson correlation

Supplementary Figure 4. Model diagnostics and evaluation. (**A**) MCMC chain convergence diagnostic ($\hat{R}$) for all bayesReact activity parameters for the TCGA data ($n = M \times C \approx 158 \cdot 10^6$). $\hat{R}$ compares the within and between chain parameter estimates, with values close to one supporting convergence on the stationary target distribution (posterior). The dashed line highlights $\hat{R} = 1.05$, and the histogram contains 50 bins, with values capped at 1.05+. (**B**) Effective sample size (ESS) for all activity parameters from the TCGA data. ESS is an MCMC chain auto-correlation diagnostic and a small ESS indicates high uncertainty of a parameter estimate due to inefficient exploration of the posterior. The dashed line highlights $ESS = 1,000$ and the histogram contains 50 bins, with values capped at 20,000+. (**C**) Diagnostics plots for the two-parameter beta model (bayesReact$_{\mathrm{2p}}$), equivalent to panel A-B. The plots depict $\hat{R}$ and ESS for each $\tau_{c,m}$ parameter. (**D**) Overview of the marginal posterior approximation process for a single activity parameter, $a_{7278,11872}$, parameterizing the miR-124-3p target motif distribution across sequences ranked by fold-change scores from a low-grade glioma (LGG) sample. Left: The observed motif distribution across the normalized combined sequence interval of all 3' UTRs. The histogram contains 30 bins, and a corresponding density line is shown, while the rug (bottom) depicts the underlying motif observations represented by the midpoint, $r_{i,7278,11872}$, of a given sequence i. Middle: Traceplot of three MCMC chains run for 3,000 iterations, including a discarded warm-up period of 500 iterations (grey shading). Right: The MCMC approximation of the marginal posterior (histogram with 50 bins). The normal approximation (black line) is defined by the posterior mean and standard deviation and is used to find the motif activity. The dashed lines show the 80% (dark) and 95% (light) credible intervals (CI). (**E**) Cumulative motif distributions sampled from the prior (right) and posterior (left) predictive distributions. The observed empirical cumulative distribution function (CDF; black) and theoretical uniform CDF (grey) are plotted together with 1,000 simulated CDFs (blue). A thousand $a_{7278,11872}$ values were randomly sampled from the prior and posterior distributions. Predictive CDFs were then generated by simulating motif occurrences under beta distributions parameterized by each sampled activity parameter, with sample sizes equal to the total number of motif observations ($n_m = 3,067$). The dashed lines depict the predictive CDFs of the 95% CI for the sampled activity parameters. (**F**) The marginal posterior approximation process for $a_{406,1142}$, which parameterizes the miR-122-5p target motif distribution in a liver hepatocellular carcinoma (LIHC) sample. Plots corresponding to panel D are depicted, showing the observed motif distribution (left), traceplot (middle), and marginal posterior distribution (right). (**G**) Prior (top) and posterior (bottom) predictive checks for $a_{406,1142}$, equivalent to analysis shown in panel E, with $n_m = 1,692$. (**H**) Pan-cancer Pearson correlation between miRNA expression and activity for 1,000 replications of the bayesReact activity inference for the miR-122-5p activity (left) and miR-124-3p activity (right). The dashed lines highlight the median correlation (dark) as well as the minimum and maximum values (light). The histograms contain 50 bins.

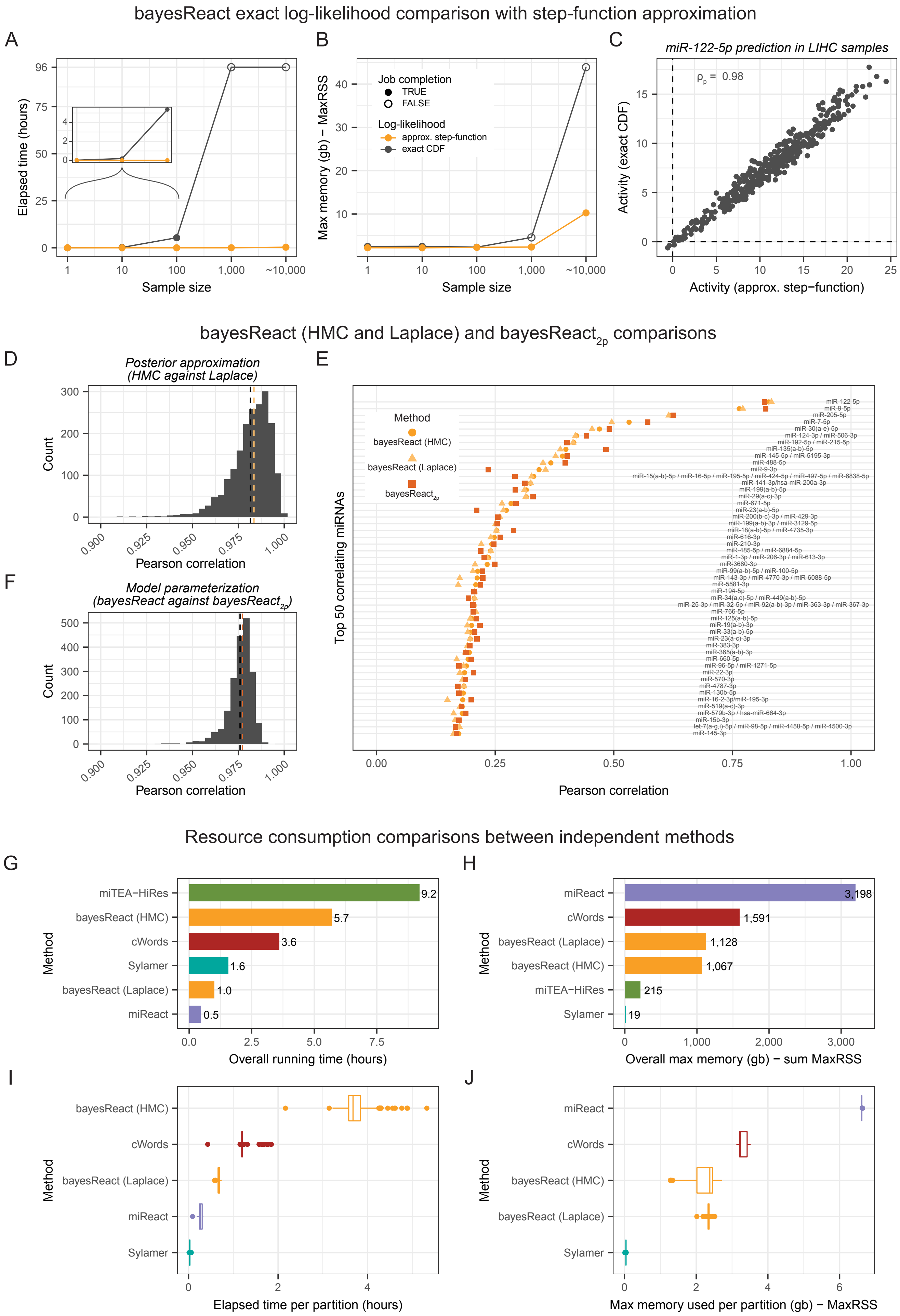
bayesReact exact log-likelihood comparison with step-function approximation
A
B
C
Elapsed time (hours)
Sample size
Max memory (gb) − MaxRSS
Job completion
TRUE
FALSE
Log-likelihood
approx. step-function
exact CDF
miR-122-5p prediction in LIHC samples
ρ_p = 0.98
Activity (exact CDF)
Activity (approx. step−function)
bayesReact (HMC and Laplace) and bayesReact$_{2p}$ comparisons
D
Posterior approximation
(HMC against Laplace)
Count
Pearson correlation
E
Method
bayesReact (HMC)
bayesReact (Laplace)
bayesReact$_{2p}$
Top 50 correlating miRNAs
Pearson correlation
F
Model parameterization
(bayesReact against bayesReact$_{2p}$)
Count
Pearson correlation
Resource consumption comparisons between independent methods
G
miTEA−HiRes 9.2
bayesReact (HMC) 5.7
cWords 3.6
Sylamer 1.6
bayesReact (Laplace) 1.0
miReact 0.5
Method
Overall running time (hours)
H
miReact 3,198
cWords 1,591
bayesReact (Laplace) 1,128
bayesReact (HMC) 1,067
miTEA−HiRes 215
Sylamer 19
Overall max memory (gb) − sum MaxRSS
I
bayesReact (HMC)
cWords
bayesReact (Laplace)
miReact
Sylamer
Elapsed time per partition (hours)
J
miReact
cWords
bayesReact (HMC)
bayesReact (Laplace)
Sylamer
Max memory used per partition (gb) − MaxRSS

Supplementary Figure 5. Evaluation of log-likelihood, posterior approximations, and computational resource comparisons. All methods were run on three CPU cores using the GenomeDK high-performance computing (HPC) facility. When performing MCMC sampling, this was done with three independent chains for 3,000 iterations with a warm-up of 500 iterations using STAN's Hamiltonian Monte Carlo (HMC) algorithm. (**A**) Comparison of elapsed running times for increasing sample sizes (number of conditions) using bayesReact with the exact likelihood (grey) and its approximation with a step-function (orange). The TCGA data was randomly sampled to create five subsets with a fixed number of samples. Please see panel B for the figure legend. Job completion indicates whether the MCMC sampling successfully finished within the allocated four-day walltime (True) or not (False). No data partitioning and parallelization were performed, and only a single motif (miR-122-5p target site) was considered due to the extreme running times using the exact log-likelihood (panel A-C). The largest sample size is n = 9,640, constituting all samples from the TCGA data (panel A-B). (**B**) Max memory usage with increasing number of samples, comparing the exact log-likelihood with that of the step-function approximation. In the two instances where bayesReact did not complete within the reserved walltime, max memory indicates usage while the job was still running. MaxRSS = Maximum resident set size (maximum amount of memory occupied by a job at any time during its execution); gb = gigabyte; CDF = cumulative distribution function. (**C**) Scatterplot of miR-122-5p activities inferred using bayesReact's exact log-likelihood and step-function approximation. All the TCGA liver hepatocellular carcinoma samples (LIHC; n = 367) are included. ρ_p = Pearson correlation coefficient. (**D**) Distribution of Pearson correlation coefficients for collapsed miRNAs sharing target sites (n = 1,941). Association across the TCGA samples (n = 9,640) was evaluated between the bayesReact activity inferred through posteriors obtained using HMC and Laplace approximation. Dashed lines highlight the mean (black) and the median (yellow). (**E**) Pearson correlation between the observed miRNA expression and inferred activity across all TCGA samples for the top 50 miRNAs matching Figure 3D. The miRNAs are ordered based on the bayesReact (HMC) results. (**F**) Pearson correlation between miRNA activities inferred using bayesReact and bayesReact$_{2p}$. Correlations were obtained for all aggregated miRNAs across all TCGA samples, and the dashed lines show the mean (black) and the median (orange). (**G**) Total run time of individual methods from initiation until completion for the full TCGA data. The methods, except miTEA-HiRes, had all 7-mers (n = 16,384) as input. Meanwhile, miTEA-HiRes only evaluates miRNAs with miRTarBase target annotations (n = 2,571) of which a subset is expressed in the TCGA data. Note that the overall run time is expected to vary with the HPC load at a given time, resulting in differing queuing times for job partitions (relevant for all methods except miTEA-HiRes, which was not parallelized). The methods are listed in decreasing order (panel G-J). (**H**) Max memory summed across all parallel partition(s). There is a trade-off between run time and memory usage: When performing data partitioning, the same motif and sequence information must be loaded for each partition. The parallelization reduces the overall run time but requires more memory allocation. (**I**) Elapsed time for each job partition across methods. The data was subdivided into partitions containing 20 samples each (n = 482), which were subsequently executed in independent job partitions. Observed discrepancies between the overall running time and the elapsed time of the subjobs are due to post-processing (merging partition output and calculating activity scores when they are not directly part of the output). (**J**) Boxplots depicting maximum memory usage for each partition (n = 482) and method.

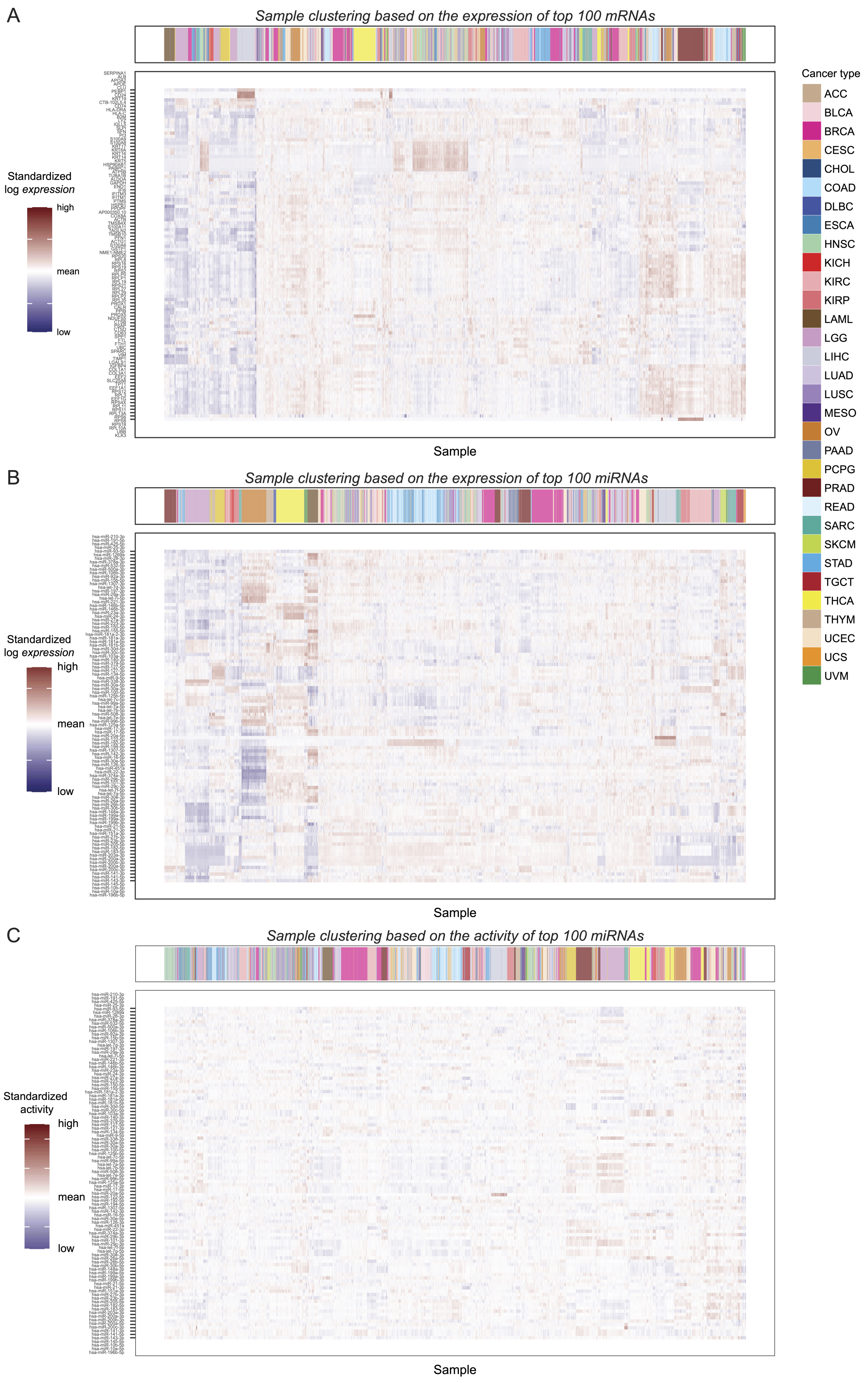
A
Sample clustering based on the expression of top 100 mRNAs
Standardized
log expression
high
mean
low
Sample
B
Sample clustering based on the expression of top 100 miRNAs
Standardized
log expression
high
mean
low
Sample
C
Sample clustering based on the activity of top 100 miRNAs
Standardized
activity
high
mean
low
Sample
Cancer type
ACC
BLCA
BRCA
CESC
CHOL
COAD
DLBC
ESCA
HNSC
KICH
KIRC
KIRP
LAML
LGG
LIHC
LUAD
LUSC
MESO
OV
PAAD
PCPG
PRAD
READ
SARC
SKCM
STAD
TGCT
THCA
THYM
UCEC
UCS
UVM

Supplementary Figure 6. Heatmaps depicting clustering of pan-cancer samples. (**A**) Heatmap of the log-transformed mRNA expression across all TCGA samples for the 100 transcripts with the highest mean RPKM values. Hierarchical agglomerative clustering (HAC) was performed on both the transcript and sample levels, with dissimilarities between observations measured using Euclidean distances and complete linkage used to evaluate distances between sets of observations. Standardized expression values are used for visualization. RPKM = reads per kilobase of transcript per million mapped reads. (**B**) Heatmap depicting the top 100 miRNAs based on the highest mean log-transformed TPM values. HAC was performed on both the miRNA transcript and sample level, and standardized values are used for visualization. TPM = transcripts per million. (**C**) Heatmap showing the standardized activity of miRNAs from panel B. Independent HAC was performed at the sample level.

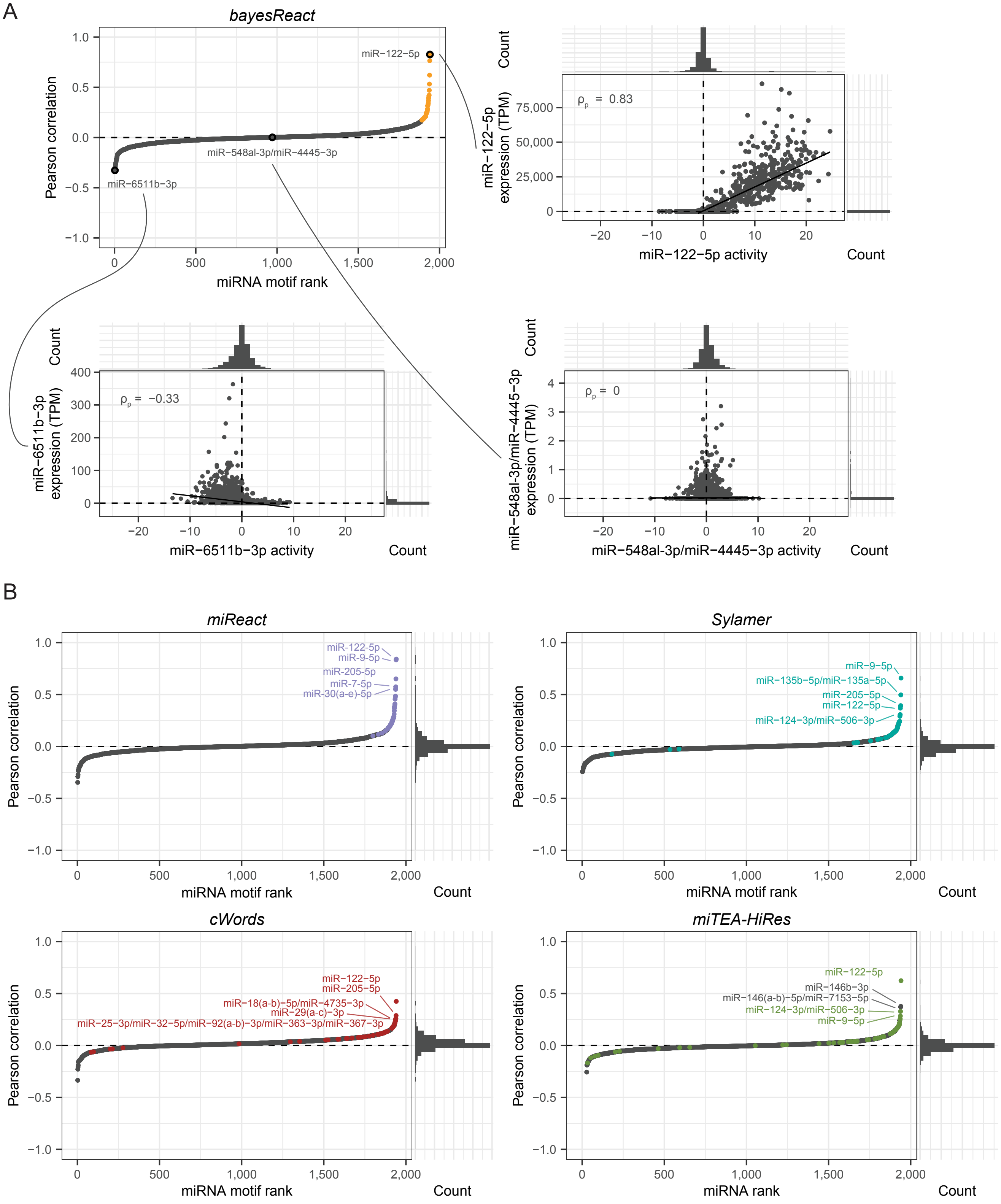
A
bayesReact
Pearson correlation
miRNA motif rank
miR−122−5p
miR−548al-3p/miR−4445−3p
miR−6511b−3p
miR−122−5p activity
miR−122−5p expression (TPM)
ρp = 0.83
Count
miR−6511b−3p activity
miR−6511b−3p expression (TPM)
ρp = −0.33
miR−548al-3p/miR−4445−3p activity
miR−548al-3p/miR−4445−3p expression (TPM)
ρp = 0
B
miReact
miR−122−5p
miR−9−5p
miR−205−5p
miR−7−5p
miR−30(a-e)−5p
Sylamer
miR−9−5p
miR−135b−5p/miR−135a−5p
miR−205−5p
miR−122−5p
miR−124−3p/miR−506−3p
cWords
miR−122−5p
miR−205−5p
miR−18(a-b)−5p/miR−4735−3p
miR−29(a-c)−3p
miR−25−3p/miR−32−5p/miR−92(a-b)−3p/miR−363−3p/miR−367−3p
miTEA-HiRes
miR−122−5p
miR−146b−3p
miR−146(a-b)−5p/miR−7153−5p
miR−124−3p/miR−506−3p
miR−9−5p
miRNA rank

Supplementary Figure 7. Overall pan-cancer performance of microRNA activity inference methods. (**A**) Pearson correlation between miRNA expression and activity across all TCGA samples (n = 9,640), equivalent to Figure 3B (top-left). miRNAs are collapsed by shared target site (n = 1,941) and ranked in increasing order. The 50 miRNAs with the largest positive correlations are highlighted in orange, while maximum, median, and minimum are shown in black. Scatterplots depict the underlying pan-cancer expression and activity for highlighted miRNAs, and corresponding frequencies are shown (histograms with 50 bins). Note that the differing distributions of no expression (point density at zero) and activity (Gaussian centered at zero) tend to produce correlation scores of approx. zero. In addition, the expression only takes values on the positive real axis while the activity score is defined on the entire real axis, prompting the two quantities to have different distributions. ρ_p = Pearson correlation coefficient. (**B**) Pearson correlation between miRNA expression and activity across all TCGA samples for different activity inference methods. Top 5 miRNAs are annotated. To show agreement between methods, the top 50 miRNAs from bayesReact (panel A) are highlighted for all methods (colored; these are also the points shown in Figure 3D).

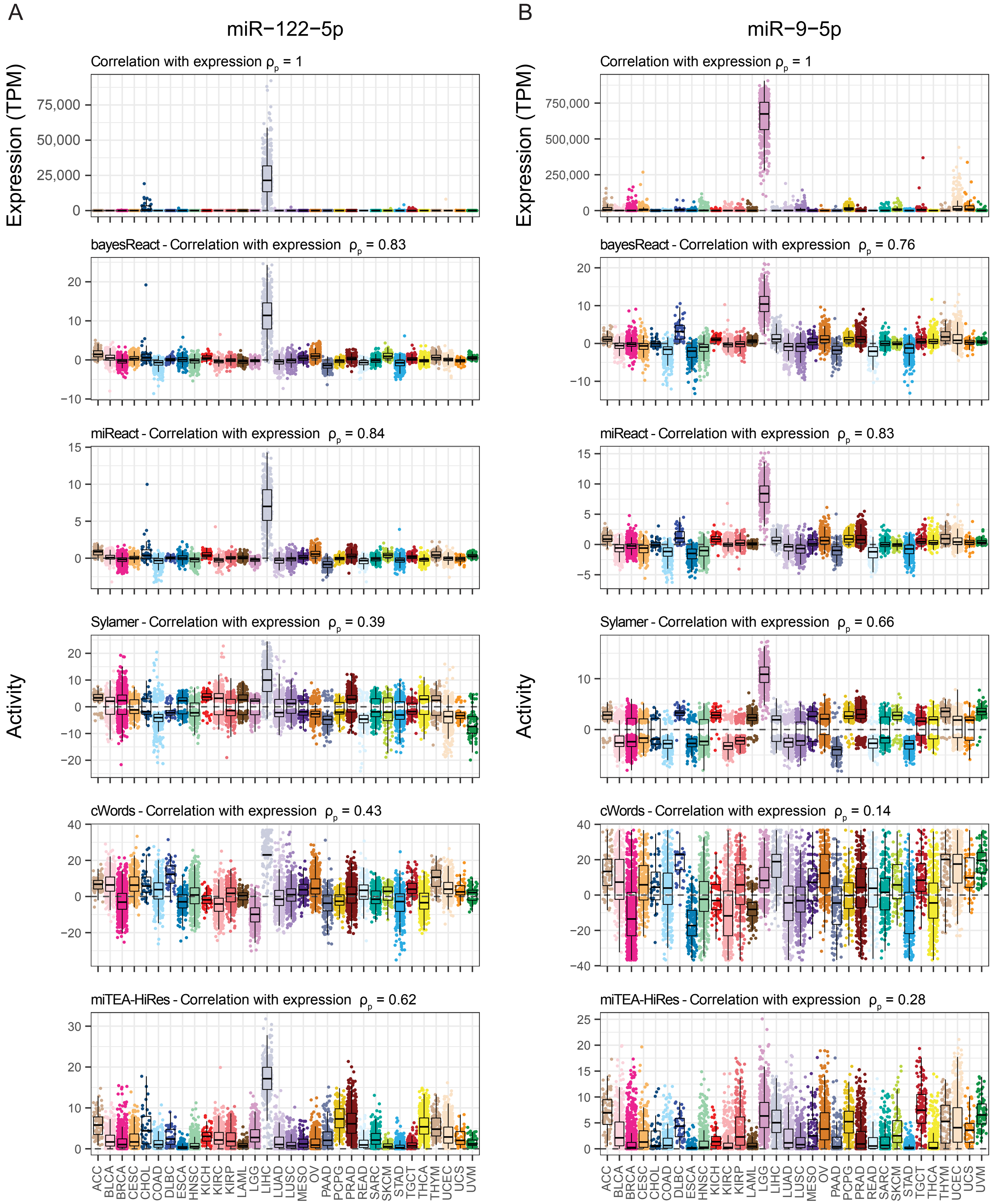
A
miR−122−5p
Correlation with expression ρp = 1
Expression (TPM)
bayesReact - Correlation with expression ρp = 0.83
miReact - Correlation with expression ρp = 0.84
Sylamer - Correlation with expression ρp = 0.39
Activity
cWords - Correlation with expression ρp = 0.43
miTEA-HiRes - Correlation with expression ρp = 0.62
B
miR−9−5p
Correlation with expression ρp = 1
bayesReact - Correlation with expression ρp = 0.76
miReact - Correlation with expression ρp = 0.83
Sylamer - Correlation with expression ρp = 0.66
cWords - Correlation with expression ρp = 0.14
miTEA-HiRes - Correlation with expression ρp = 0.28
ACC BLCA BRCA CESC CHOL COAD DLBC ESCA HNSC KICH KIRC KIRP LAML LGG LIHC LUAD LUSC MESO OV PAAD PCPG PRAD READ SARC SKCM STAD TGCT THCA THYM UCEC UCS UVM

Supplementary Figure 8. Cancer-type-specific miR-122-5p and miR-9-5p activity comparison between methods. miR-122-5p and miR-9-5p are among the miRNAs with the strongest expression and activity correlation across the independent inference methods. (**A**) Comparison of observed miR-122-5p expression (top) and inferred activities (bottom rows) across the TCGA primary tumor samples (n = 9,640). The order of cancer types (x-axis) is equivalent to Figure 4A. LIHC = liver hepatocellular carcinoma (grey); ρ_p = Pearson correlation coefficient. (**B**) Corresponding plots for miR-9-5p. LGG = low-grade glioma (light pink).

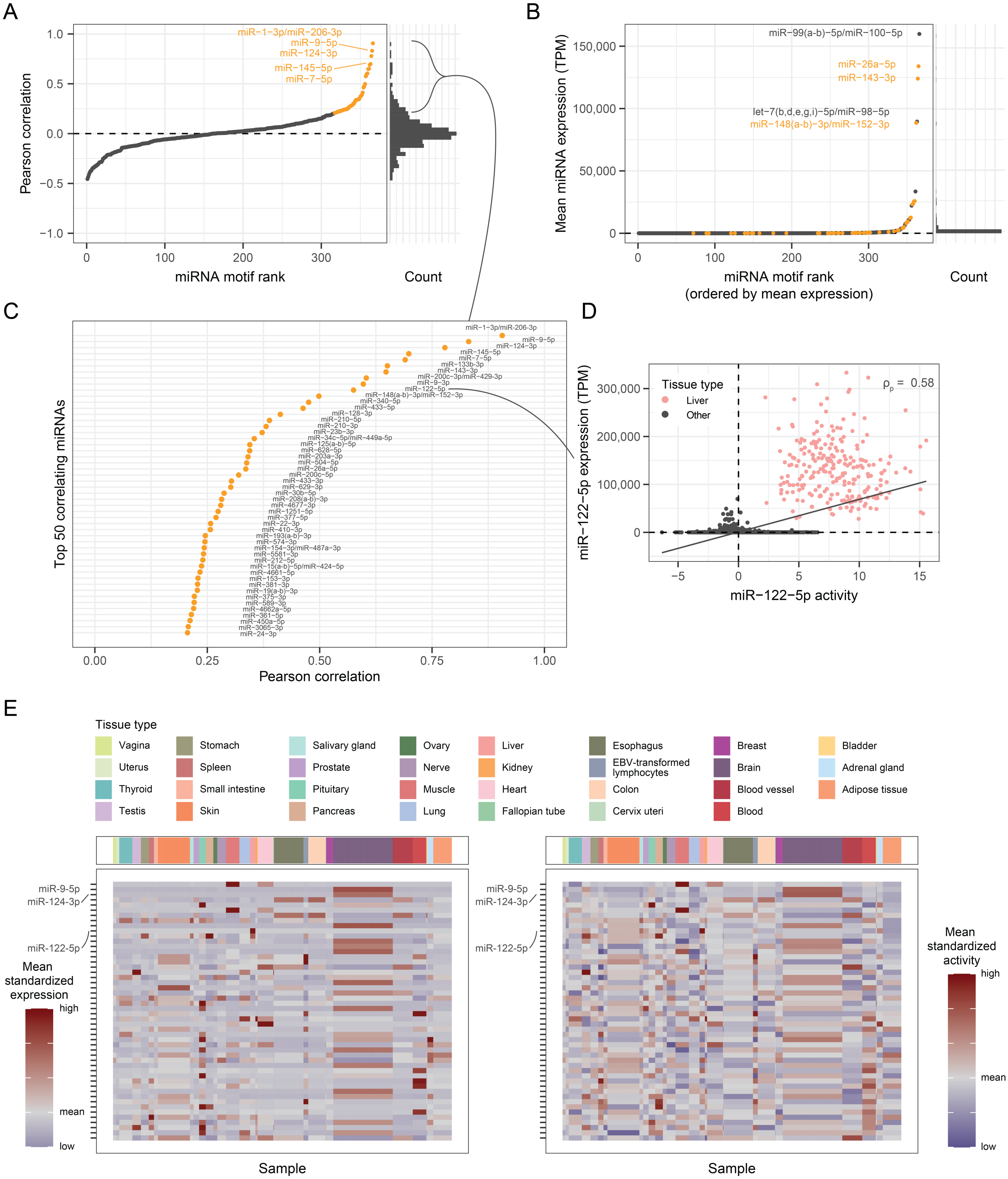
A
Pearson correlation
miRNA motif rank
Count
miR−1−3p/miR−206−3p
miR−9−5p
miR−124−3p
miR−145−5p
miR−7−5p
B
Mean miRNA expression (TPM)
miRNA motif rank
(ordered by mean expression)
Count
miR−99(a-b)−5p/miR−100−5p
miR−26a−5p
miR−143−3p
let−7(b,d,e,g,i)−5p/miR−98−5p
miR−148(a-b)−3p/miR−152−3p
C
Top 50 correlating miRNAs
Pearson correlation
D
miR−122−5p expression (TPM)
miR−122−5p activity
Tissue type
Liver
Other
ρp = 0.58
E
Tissue type
Vagina
Uterus
Thyroid
Testis
Stomach
Spleen
Small intestine
Skin
Salivary gland
Prostate
Pituitary
Pancreas
Ovary
Nerve
Muscle
Lung
Liver
Kidney
Heart
Fallopian tube
Esophagus
EBV-transformed lymphocytes
Colon
Cervix uteri
Breast
Brain
Blood vessel
Blood
Bladder
Adrenal gland
Adipose tissue
miR-9-5p
miR-124-3p
miR-122-5p
Mean standardized expression
high
mean
low
Sample
Mean standardized activity
high
mean
low
Sample

Supplementary Figure 9. microRNA activity inference across healthy tissue samples. (**A**) Expression and bayesReact activity correlation for collapsed miRNAs sharing their target site (n = 366) across the GTEx tissue samples (n = 15,398). The miRNAs are ranked by Pearson correlation, and the top 5 are annotated. On the right is depicted the corresponding histogram containing 50 bins. Corresponds to Figure 3F. (**B**) Mean miRNA expression across the GTEx samples, with top correlating miRNAs highlighted (from panel A) and the top five annotated. (**C**) The top 50 correlating miRNAs (panel A) are shown in detail. Plot corresponds to Figure 3G. (**D**) Inferred activity of the liver-specific miR-122-5p plotted against its observed expression and linear regression line shown (black). $\rho_p =$ Pearson correlation coefficient. (**E**) Heatmaps for the top correlating miRNAs (panel C) clustered by tissue type (n = 31). The mean tissue type expression (left) and bayesReact activity (right) are shown. Values are standardized for visualization purposes. Tissue labels appear in the same order as in the heatmaps.

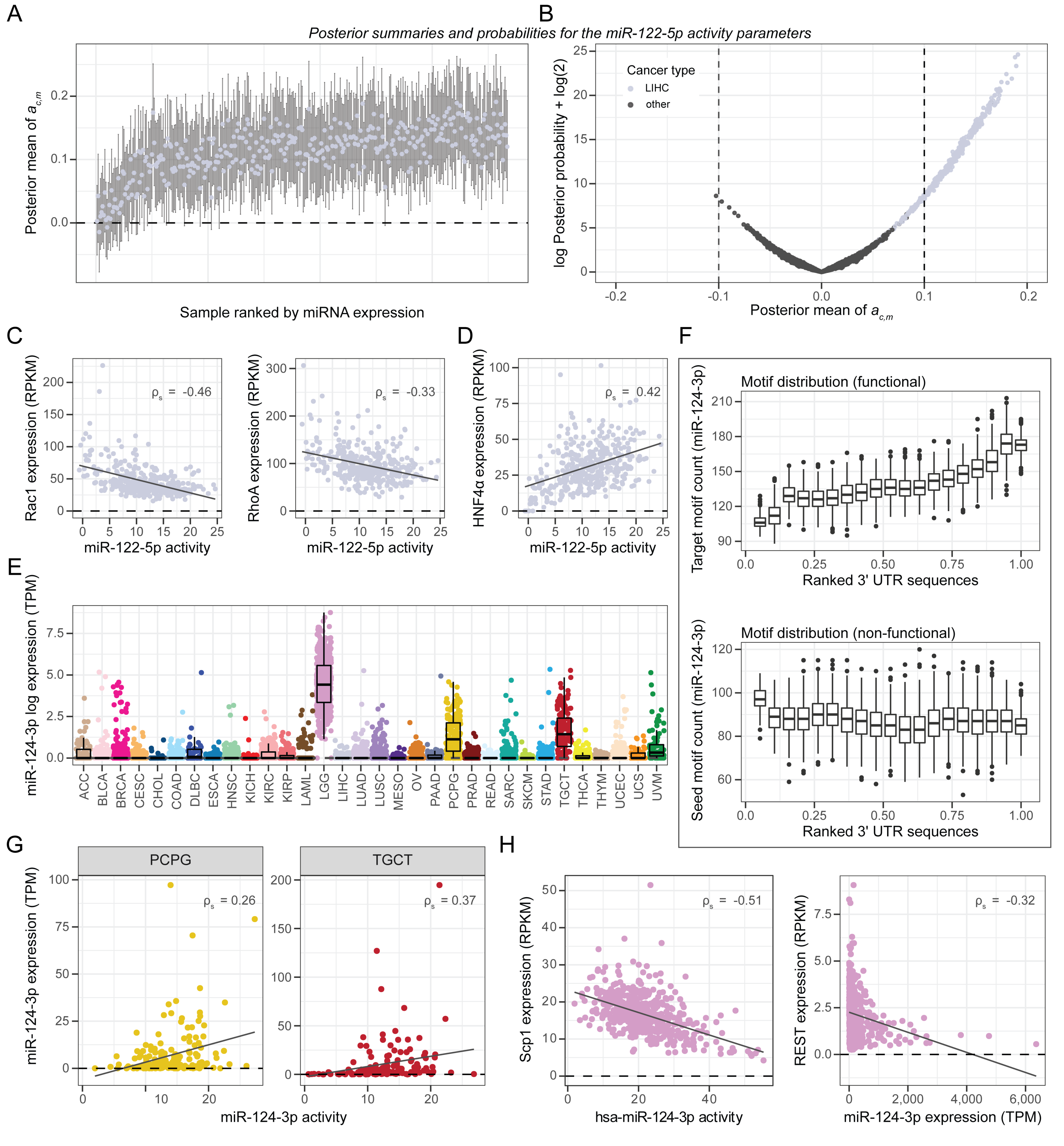

Supplementary Figure 10. Cancer-type-specific miR-122-5p and miR-124-3p activities. (**A**) The (marginal) posterior means and 99% credible intervals (CIs) of the miR-122-5p activity parameter $a_{c,m}$ for all liver hepatocellular carcinoma (LIHC) samples (n = 367). Samples are ordered by the miR-122-5p expression. (**B**) Posterior mean for the miR-122-5p activity parameter from each TCGA sample plotted against the corresponding posterior tail probability (see eq. 6). (**C**) The miR-122-5p activity plotted against the expression of two target genes, Rac1 (left) and RhoA (right), for the LIHC samples. ρ_s = Spearman correlation coefficient. RPKM = reads per kilobase of transcript per million mapped reads. (**D**) The miR-122-5p activity plotted against the expression of HNF4α; a transcription factor promoting the transcription of the miR-122 host gene. (**E**) Log-transformed miR-124-3p expression across all primary tumor samples from the TCGA data. One is added to the expression provided as transcripts per million (TPM + 1). (**F**) miR-124-3p target site (top) and seed site (bottom) distribution across the 3' UTR sequences. The combined sequence interval is divided into 20 bins, and each boxplot depicts the motif count within a bin for each low-grade glioma (LGG) sample (n = 509). The miR-124-3p target motif occurs 3,067 times across 2,624 3' UTRs, while the seed motif occurs 1,824 times in 1,661 3' UTRs. UTR = untranslated region. (**G**) The miR-124-3p activity plotted against its expression in the pheochromocytomas and paragangliomas (PCPG) samples (n = 182; left) and testicular germ-cell tumors (TGCT) samples (n = 139; right). A linear regression line and Spearman correlation are shown. TPM = transcripts per million. (**H**) The miR-124-3p activity plotted against the expression of its known target Scp1 (CTDSP1; left), and the miR-124-3p expression plotted against its downstream target REST (right) for the low-grade glioma samples. A linear regression line and Spearman correlation are depicted.

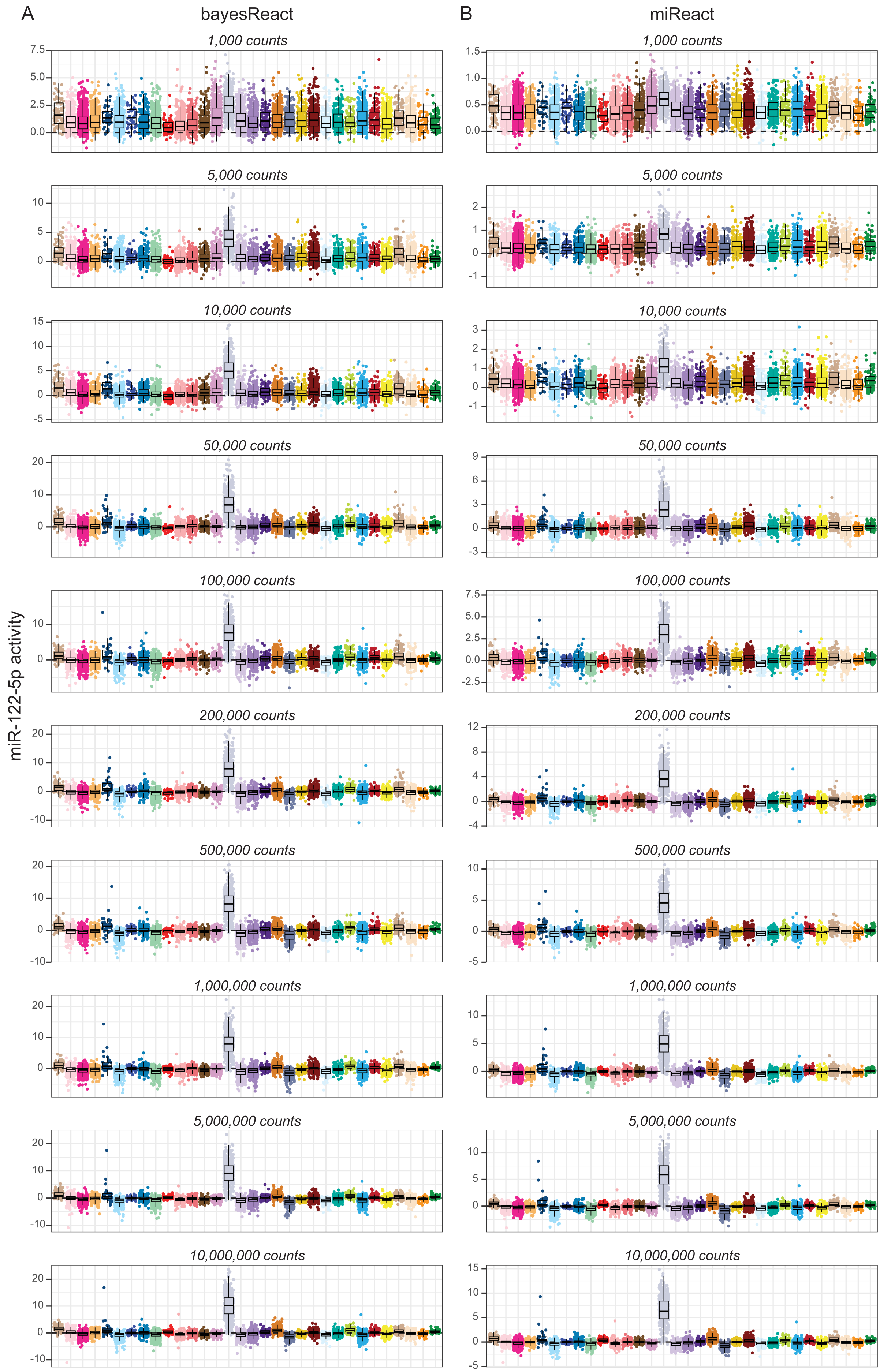
A
bayesReact
B
miReact
1,000 counts
5,000 counts
10,000 counts
50,000 counts
100,000 counts
200,000 counts
500,000 counts
1,000,000 counts
5,000,000 counts
10,000,000 counts
miR-122-5p activity

Supplementary Figure 11. miR-122-5p activity inference based on differing degrees of library count down-sampling. (**A**) miR-122-5p activity inference using bayesReact, with each row showing results for increasing library count sizes. The cancer type ordering is the same as Figure 4 panel A, with liver hepatocellular carcinoma (LIHC) samples shown in grey. (**B**) Corresponding miR-122-5p activity inference using miReact.

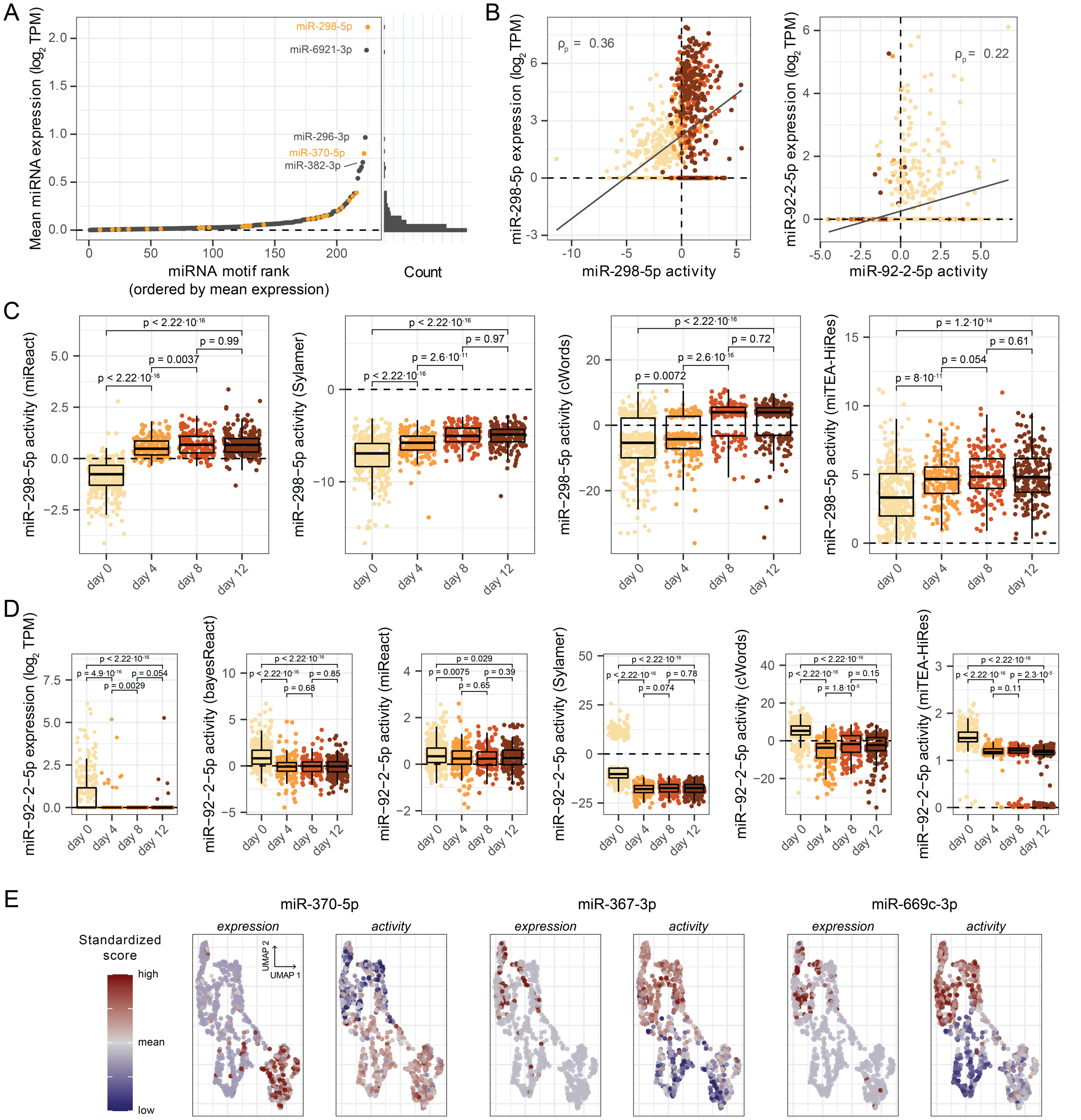

Supplementary Figure 12. Recovering microRNA activities at the single-cell level from mouse embryonic stem cells. (**A**) Mean miRNA expression across all cells ordered by the mean values (left) and corresponding histogram containing 50 bins (right). The top 25 miRNAs with the highest correlation between expression and bayesReact activity are highlighted in orange. TPM = transcripts per million. (**B**) miR-298-5p (left) and miR-92-2-5p (right) activity plotted against the expression and linear regression line depicted. Points (cells) are colored by sample extraction time. (**C**) Boxplots depicting the miR-298-5p activity over time, inferred using miReact, Sylamer, cWords, and miTEA-HiRes. P-values were obtained from two-sided Wilcoxon rank-sum tests. (**D**) miR-92-2-5p expression (left) and activity (right) obtained through bayesReact, miReact, Sylamer, cWords, and miTEA-HiRes. A two-sided Wilcoxon rank-sum test was performed and annotated for each compared time point. (**E**) Dimension reduction plots based on UMAP coordinates depicting cell clustering, with expression and activity annotated. The expression and activity are standardized to have a mean of zero and a standard deviation of one. Please see Figure 6, panel G, for time point annotations.

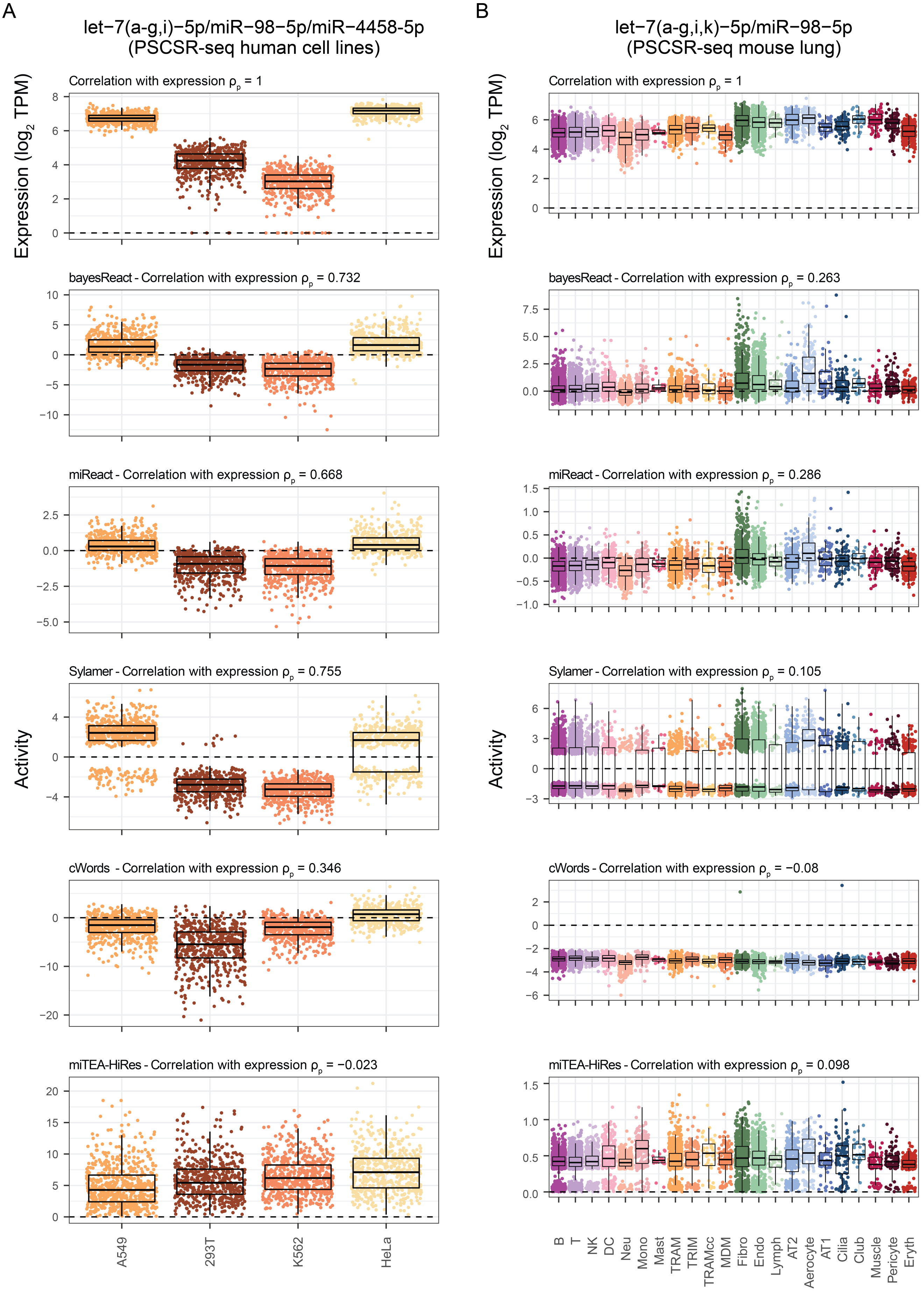
A
let−7(a-g,i)−5p/miR−98−5p/miR−4458-5p
(PSCSR-seq human cell lines)
B
let−7(a-g,i,k)−5p/miR−98−5p
(PSCSR-seq mouse lung)
Expression (log2 TPM)
Activity
Correlation with expression ρp = 1
bayesReact - Correlation with expression ρp = 0.732
bayesReact - Correlation with expression ρp = 0.263
miReact - Correlation with expression ρp = 0.668
miReact - Correlation with expression ρp = 0.286
Sylamer - Correlation with expression ρp = 0.755
Sylamer - Correlation with expression ρp = 0.105
cWords - Correlation with expression ρp = 0.346
cWords - Correlation with expression ρp = −0.08
miTEA-HiRes - Correlation with expression ρp = −0.023
miTEA-HiRes - Correlation with expression ρp = 0.098
A549
293T
K562
HeLa
B
T
NK
DC
Neu
Mono
Mast
TRAM
TRIM
TRAMcc
MDM
Fibro
Endo
Lymph
AT2
Aerocyte
AT1
Cilia
Club
Muscle
Pericyte
Eryth

Supplementary Figure 13. let-7 microRNA family expression and activity across PSCSR-seq datasets. The let-7 family is among the top correlating miRNAs in both mouse and human data. (**A**) Collapsed let-7(a-g,i)-5p/miR-98-5p/miR-4458-5p expression (top) and activity (bottom) across four different human cell lines. (**B**) Corresponding let-7(a-g,i,k)-5p/miR-98-5p expression (top) and activity (bottom) across mouse lung cells stratified by cell type. B = B cell; T = T cell; NK = Natural killer cell; DC = dendritic cell; Neu = Neutrophil; Mono = Monocyte; Mast = Mast cell; TRAM = Tissue-resident alveolar macrophage; TRIM = Tissue-resident interstitial macrophage; TRAMcc = TRAM in G2M cell-cycle phase; MDM = Monocyte-derived macrophage; Fibro = Fibroblast; Endo = general capillary endothelial cell; Lymph = Lymphatic endothelial cell; AT2 = Alveolar type II cell; Aerocyte = Alveolar endothelial cell subtype; AT1 = Alveolar type I cell; Cilia = Ciliated epithelial cell; Club = Nonciliated bronchiolar exocrine cell (club cell); Eryth = Erythroid cell.

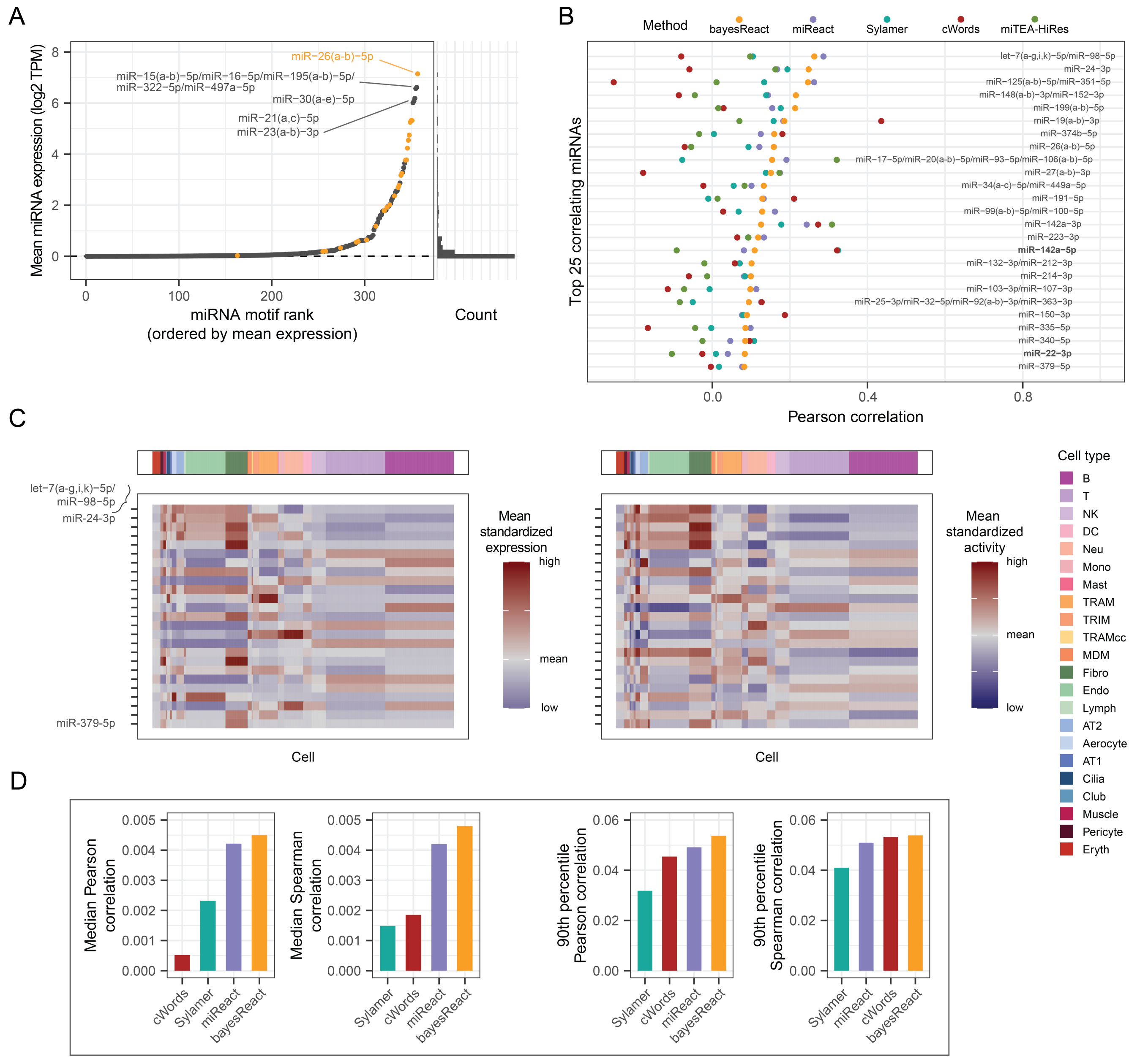

Supplementary Figure 14. MicroRNA expression and activity across cells from mouse lung biopsies. (**A**) Ordered mean miRNA expression with top five miRNAs labelled (left) and corresponding 50 bin histogram (right). miRNAs are collapsed by a shared target site. The top 25 miRNAs with the largest correlation between expression and bayesReact activity are highlighted in orange (matches Figure 7 panel E). TPM = transcripts per million. (**B**) Top correlating miRNAs based on expression and activity across all mouse lung cells (n = 9,403). miRNAs originally mentioned by Li et al. are highlighted with bold. (**C**) Heatmap of the mean cell type expression (left) and bayesReact activity (right) for the top 25 correlating miRNAs (order matches panel B). Values are standardized for visualization purposes. B = B cell; T = T cell; NK = Natural killer cell; DC = dendritic cell; Neu = Neutrophil; Mono = Monocyte; Mast = Mast cell; TRAM = Tissue-resident alveolar macrophage; TRIM = Tissue-resident interstitial macrophage; TRAMcc = TRAM in G2M cell-cycle phase; MDM = Monocyte-derived macrophage; Fibro = Fibroblast; Endo = general capillary endothelial cell; Lymph = Lymphatic endothelial cell; AT2 = Alveolar type II cell; Aerocyte = Alveolar endothelial cell subtype; AT1 = Alveolar type I cell; Cilia = Ciliated epithelial cell; Club = Nonciliated bronchiolar exocrine cell (club cell); Eryth = Erythroid cell. (**D**) Median (left) and 90th percentile (right) correlation coefficients between observed expression and inferred activity for different methods. Results are based on all collapsed miRNAs (n = 357) across all cells, with miTEA-HiRes excluded due to lacking activity profiles for a large fraction of the miRNAs (n = 139).

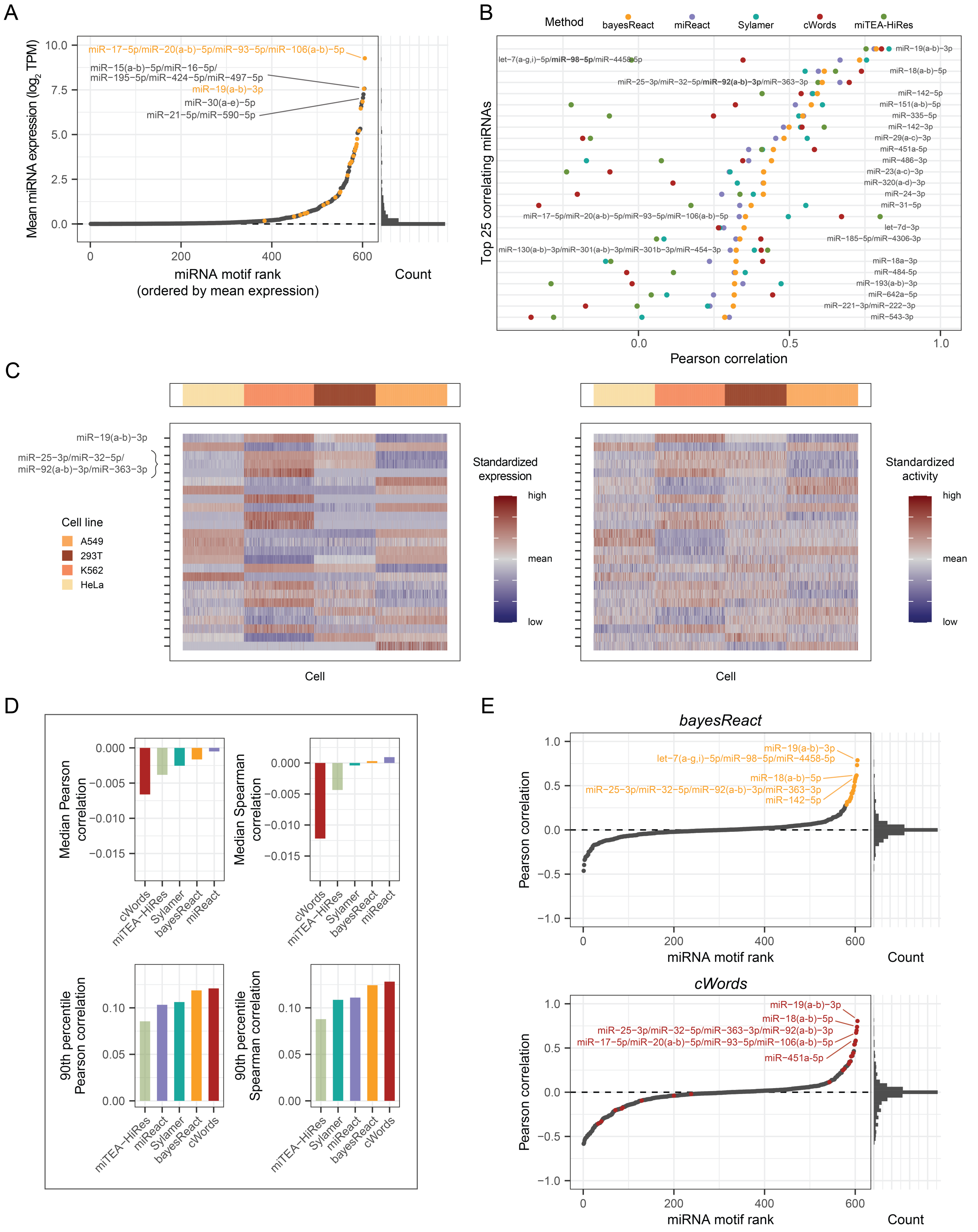
A
B
C
D
E
Mean miRNA expression ($\log_2$ TPM)
miRNA motif rank
(ordered by mean expression)
Count
miR−17−5p/miR−20(a-b)−5p/miR−93−5p/miR−106(a-b)−5p
miR−15(a-b)−5p/miR−16−5p/
miR−195−5p/miR−424−5p/miR−497−5p
miR−19(a-b)−3p
miR−30(a-e)−5p
miR−21−5p/miR−590−5p
Method
bayesReact
miReact
Sylamer
cWords
miTEA-HiRes
Top 25 correlating miRNAs
Pearson correlation
Cell line
A549
293T
K562
HeLa
Standardized expression
Standardized activity
high
mean
low
Cell
Median Pearson correlation
Median Spearman correlation
90th percentile Pearson correlation
90th percentile Spearman correlation
bayesReact
cWords
Pearson correlation
miRNA motif rank
Count

Supplementary Figure 15. Human cell line microRNA expression and activity. (**A**) Mean collapsed expression for each miRNA (n = 606) across cells from four cell lines (n = 2,310; left) and corresponding histogram (right). (**B**) miRNAs with highest Pearson correlation (n = 25) between expression and bayesReact activity (matching highlighted miRNAs in panel D). (**C**) Heatmaps of top 25 miRNAs (panel B), depicting expression (left) and bayesReact activity (right) for each cell, with scores standardized for visualization. (**D**) Median (top) and 90th percentile (bottom) correlation coefficients for all collapsed miRNAs (n = 606) between expression and activity inferred from different methods. miTEA-HiRes only has activity inference for 584 of 605 expressed miRNAs (low opacity). (**E**) Ordered Pearson correlation (left) between expression and activity across cells from all cell lines and corresponding 50 bin histogram (right). bayesReact (top) has a similar positive tail to cWords (bottom), but does not exhibit a similarly extreme negative tail, underscoring results from panel C.

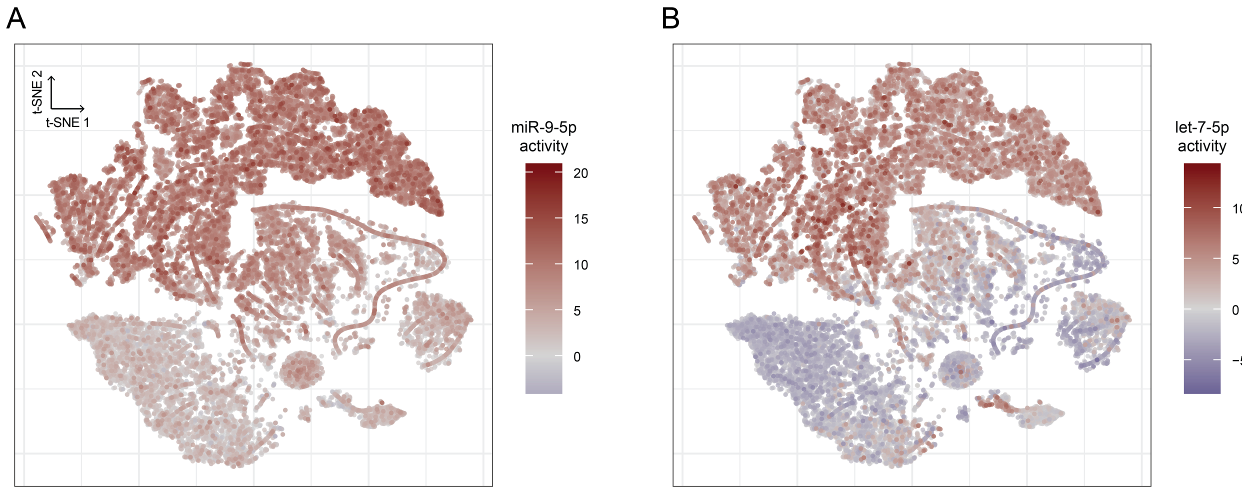

Supplementary Figure 16. Inferred miR-9-5p and let-7-5p activities during spinal cord development in mice. (**A**) t-SNE plot depicting cell clustering from the developing embryonic spinal cord in mice (n = 38,976), with the miR-9-5p activity annotated. See Figure 7G for corresponding cell type annotations. (**B**) The let-7(a-g,i,k)-5p activity across embryonic spinal cord cells.

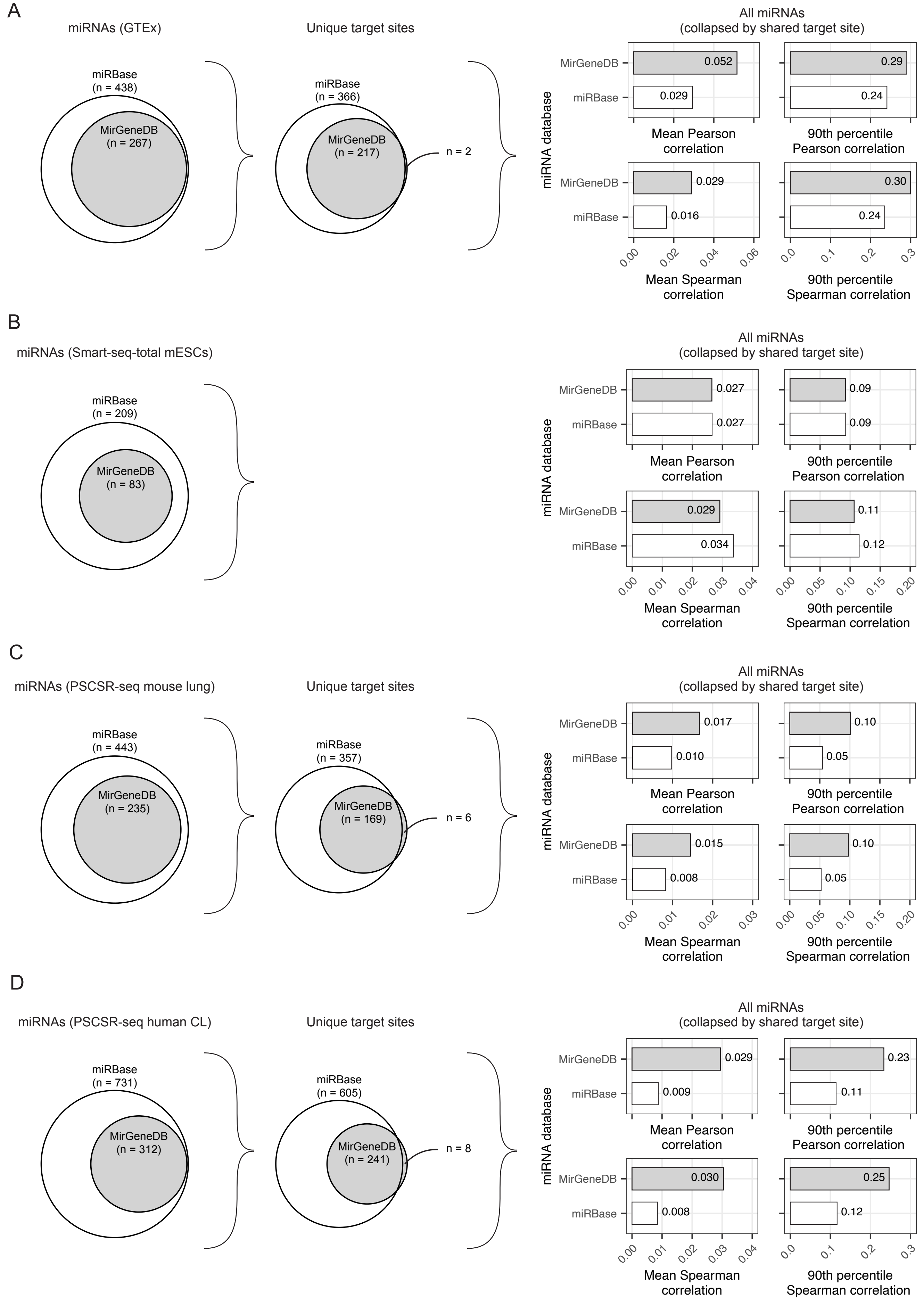
A
miRNAs (GTEx)
miRBase
(n = 438)
MirGeneDB
(n = 267)
Unique target sites
miRBase
(n = 366)
MirGeneDB
(n = 217)
n = 2
All miRNAs
(collapsed by shared target site)
miRNA database
MirGeneDB
miRBase
0.052
0.029
0.29
0.24
Mean Pearson correlation
90th percentile Pearson correlation
0.029
0.016
0.30
0.24
Mean Spearman correlation
90th percentile Spearman correlation
B
miRNAs (Smart-seq-total mESCs)
miRBase
(n = 209)
MirGeneDB
(n = 83)
All miRNAs
(collapsed by shared target site)
0.027
0.027
0.09
0.09
0.029
0.034
0.11
0.12
C
miRNAs (PSCSR-seq mouse lung)
miRBase
(n = 443)
MirGeneDB
(n = 235)
Unique target sites
miRBase
(n = 357)
MirGeneDB
(n = 169)
n = 6
All miRNAs
(collapsed by shared target site)
0.017
0.010
0.10
0.05
0.015
0.008
0.10
0.05
D
miRNAs (PSCSR-seq human CL)
miRBase
(n = 731)
MirGeneDB
(n = 312)
Unique target sites
miRBase
(n = 605)
MirGeneDB
(n = 241)
n = 8
All miRNAs
(collapsed by shared target site)
0.029
0.009
0.23
0.11
0.030
0.008
0.25
0.12

Supplementary Figure 17. Comparison of miRBase and MirGeneDB microRNA annotations across multiple independent datasets. (**A**) miRBase annotated miRNAs expressed in GTEx with subset present in MirGeneDB (left) and corresponding unique target sites (middle). Resulting mean Pearson and Spearman correlation and 90th percentile correlations for expression and activity across all samples are shown to the right. (**B**) Subset of expressed miRBase miRNAs annotated in MirGeneDB for the Smart-seq-total sequenced mouse embryonic stem cells (mESCs). Expression is combined for the mature 5p and 3p arms, and only miRNAs present in at least 1% of cells (n = 209) are included. (**C-D**) Equivalent plots as panel A for the PSCSR-seq datasets.

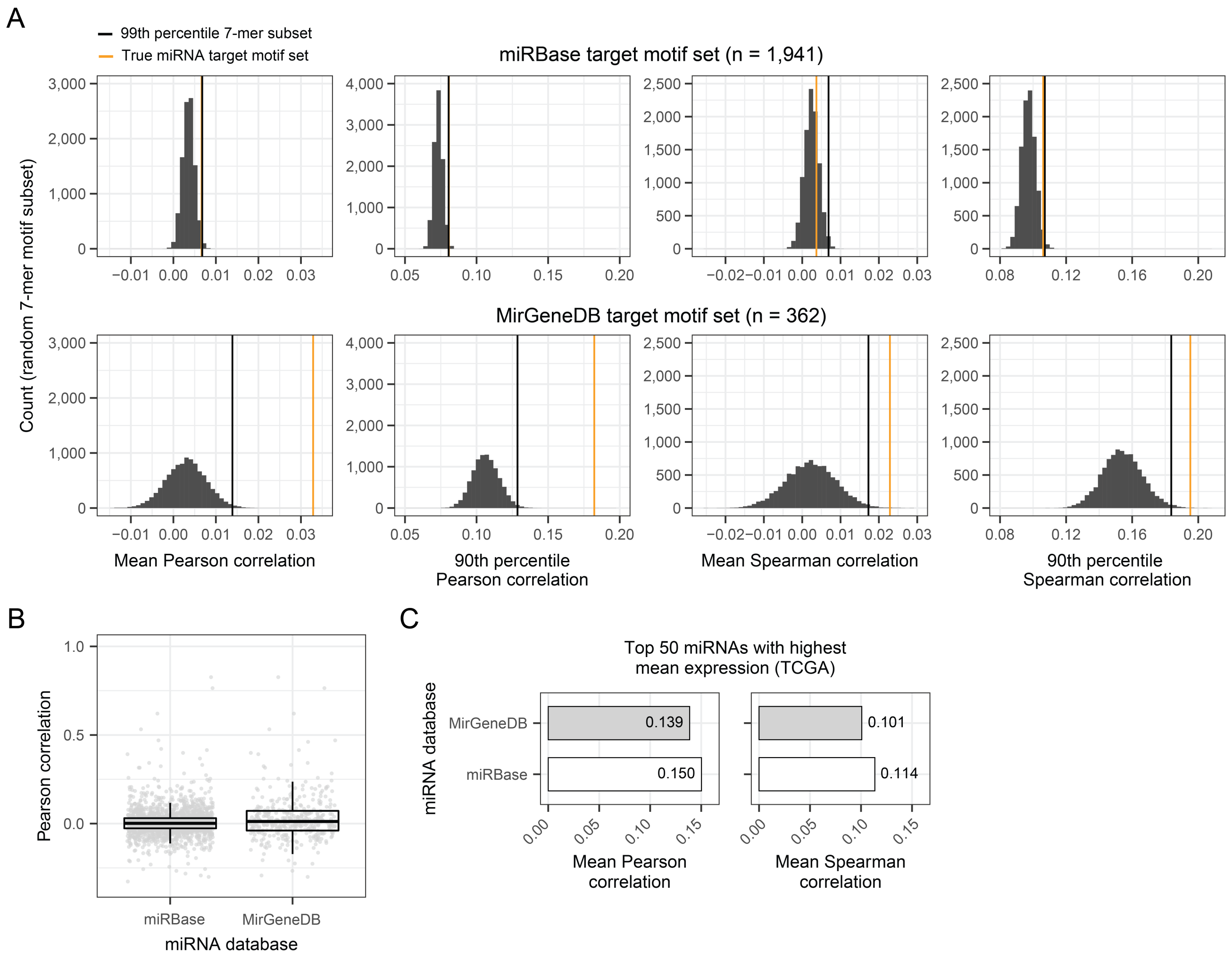

Supplementary Figure 18. miRBase and MirGeneDB comparison for pan-cancer TCGA data. (**A**) Permutation tests depicting empirical null distributions for correlation statistics based on miRBase (top) or MirGeneDB (bottom) miRNA expression profiles and randomly assigned 7-mer activities. True miRNA target motif statistics (orange) and 99th percentiles (black) are shown. (**B**) Pearson correlation for all miRNAs annotated by miRBase (n = 1,941) and MirGeneDB (n = 362). (**C**) Mean Pearson and Spearman correlation for miRNAs with the largest mean expression (n = 50).

Supplementary Tables

This section contains the following supplementary tables:

- **Supplementary Table 1.** Overview of cancer types in the TCGA data.
- **Supplementary Table 3.** Names and target sites of the top 50 correlating microRNAs from the TCGA data.

Cancer type	Cancer name	Primary site	# samples
BRCA	Breast Invasive Carcinoma	Breast	1068
UCEC	Uterine Corpus Endometrial Carcinoma	Uterus	530
LGG	Brain Lower Grade Glioma	Brain	509
THCA	Thyroid Carcinoma	Thyroid	504
LUAD	Lung Adenocarcinoma	Lung	503
KIRC	Kidney Renal Clear Cell Carcinoma	Kidney	502
HNSC	Head and Neck Squamous Cell Carcinoma	Head and Neck	497
PRAD	Prostate Adenocarcinoma	Prostate	493
LUSC	Lung Squamous Cell Carcinoma	Lung	475
COAD	Colon Adenocarcinoma	Colorectal	434
OV	Ovarian Serous Cystadenocarcinoma	Ovary	419
STAD	Stomach Adenocarcinoma	Stomach	408
BLCA	Bladder Urothelial Carcinoma	Bladder	403
LIHC	Liver Hepatocellular Carcinoma	Liver	367
CESC	Cervical Squamous Cell Carcinoma and Endocervical Adenocarcinoma	Cervix	304
KIRP	Kidney Renal Papillary Cell Carcinoma	Kidney	291
SARC	Sarcoma	Soft Tissue	257
ESCA	Esophageal Carcinoma	Esophagus	183
PCPG	Pheochromocytoma and Paraganglioma	Adrenal Gland	182
PAAD	Pancreatic Adenocarcinoma	Pancreas	178
LAML	Acute Myeloid Leukemia	Bone Marrow	173
READ	Rectum Adenocarcinoma	Colorectal	159
TGCT	Testicular Germ Cell Tumors	Testis	139
THYM	Thymoma	Thymus	120
SKCM	Skin Cutaneous Melanoma	Skin	97
MESO	Mesothelioma	Pleura	87
UVM	Uveal Melanoma	Eye	80
ACC	Adrenocortical Carcinoma	Adrenal Gland	79
KICH	Kidney Chromophobe	Kidney	66
UCS	Uterine Carcinosarcoma	Uterus	57
DLBC	Lymphoid Neoplasm Diffuse Large B−cell Lymphoma	Lymph Nodes	47
CHOL	Cholangiocarcinoma	Bile Duct	36

Supplementary Table 1. Overview of cancer types in the TCGA data. The table depicts all cancer types included from the TCGA data, and contains the abbreviated cancer type (column 1); full name of the cancer type (column 2); tissue origin of the primary tumor (column 3); and number of samples assigned to each cancer type (column 4).

miRNA	# miRNAs sharing target site	Target site
miR−122−5p	1	ACACTCC
miR−9−5p	1	ACCAAAG
miR−205−5p	1	ATGAAGG
miR−7−5p	1	GTCTTCC
miR−30a−5p/miR−30b−5p/miR−30c−5p/miR−30d−5p/miR−30e−5p	5	TGTTTAC
miR−124−3p/miR−506−3p	2	GTGCCTT
miR−192−5p/miR−215−5p	2	TAGGTCA
miR−135a−5p/miR−135b−5p	2	AAGCCAT
miR−145−5p/miR−5195−3p	2	AACTGGA
miR−488−5p	1	TATCTGG
miR−9−3p	1	AGCTTTA
miR−15a−5p/miR−15b−5p/miR−16−5p/miR−195−5p/miR−424−5p/miR−497−5p/miR−6838−5p	7	TGCTGCT
miR−141−3p/miR−200a−3p	2	CAGTGTT
miR−199a−5p/miR−199b−5p	2	ACACTGG
miR−29a−3p/miR−29b−3p/miR−29c−3p	3	TGGTGCT
miR−671−5p	1	GGCTTCC
miR−23a−5p/miR−23b−5p	2	GGAACCC
miR−200b−3p/miR−200c−3p/miR−429-3p	3	CAGTATT
miR−199a−3p/miR−199b−3p/miR−3129−5p	3	ACTACTG
miR−18a−5p/miR−18b−5p/miR−4735−3p	3	GCACCTT
miR−616−3p	1	CAATGAC
miR−210−3p	1	ACGCACA
miR−485−5p/miR−6884−5p	2	CAGCCTC
miR−1−3p/miR−206-3p/miR−613-3p	3	ACATTCC
miR−3680−3p	1	ATGCAAA
miR−99a−5p/miR−99b−5p/miR−100−5p	3	TACGGGT
miR−143−3p/miR−4770-3p/miR−6088-5p	3	TCATCTC
miR−5581−3p	1	GCATGGA
miR−194−5p	1	CTGTTAC
miR−34a−5p/miR−34c−5p/miR−449a/miR−449b−5p	4	CACTGCC
miR−25−3p/miR−32−5p/miR−92a−3p/miR−92b−3p/miR−363−3p/miR−367−3p	6	GTGCAAT
miR−766−5p	1	TTCCTCC
miR−125a−5p/miR−125b−5p	2	CTCAGGG
miR−19a−3p/miR−19b−3p	2	TTTGCAC
miR−33a−5p/miR−33b−5p	2	CAATGCA
miR−23a−3p/miR−23b−3p/miR−23c	3	AATGTGA
miR−383−3p	1	AGTGCTG
miR−365a−3p/miR−365b−3p	2	GGGCATT
miR−660−5p	1	AATGGGT
miR−96−5p/miR−1271−5p	2	GTGCCAA
miR−22−3p	1	GGCAGCT
miR−570−3p	1	TGTTTTC
miR−4787−3p	1	GGCGCAT
miR−130b−5p	1	GAAAGAG
miR−16−2−3p/miR−195−3p	2	AATATTG
miR−519a−3p/miR−519b−3p/miR−519c−3p	3	TGCACTT
miR−579−3p/miR−664b−3p	2	CAAATGA
miR−15b−3p	1	ATGATTC
let−7a−5p/let−7b−5p/let−7c−5p/let−7d−5p/let−7e−5p/let−7f−5p/let−7g−5p/let−7i−5p/miR−98−5p/miR−4458-5p/miR−4500-3p	11	CTACCTC
miR−145−3p	1	AGGAATC

Supplementary Table 3. Names and target sites of the top 50 correlating microRNAs from the TCGA data. The table includes the names (column 1) of the top 50 miRNAs with the highest Pearson correlation between expression and bayesReact activity. The miRNAs are collapsed based on shared target site (columns 2-3).
